# Supplementary material for: Rethinking the Esterquats: Synthesis, Stability, Ecotoxicity and Applications of Esterquats Incorporating Analogs of Betaine or Choline as the Cation in Their Structure
Source: Int J Mol Sci. 2024 May 25;25(11):5761. doi: 10.3390/ijms25115761 (PMC11171562; doi:10.3390/ijms25115761)
Supplement: Supplementary file 1 [file ijms-25-05761-s001.zip › ijms-2984627-supplementary.pdf]

Rethinking the Esterquats: Synthesis, Stability, Ecotoxicity and Applications of Esterquats Incorporating Analogs of Betaine or Choline as the Cation in Their Structure

Marcin Wysocki,[1] Witold Stachowiak,[2] Mikołaj Smolibowski,[2] Adriana Olejniczak,[2] Michał Niemczak*[2], Julia L. Shamshina*[3]

[1] Chair and Department of Inorganic and Analytical Chemistry, Poznan University of Medical Sciences, Rokietnicka 3, Poznan 60-806, Poland

[2] Faculty of Chemical Technology, Poznan University of Technology, Berdychowo 4, Poznan 60-965, Poland

[3] Fiber and Biopolymer Research Institute, Department of Plant and Soil Science, Texas Tech University, Lubbock, TX, USA; jshamshi@ttu.edu

*Corresponding authors at: Michał Niemczak, Poznan University of Technology, Berdychowo 4, Poznan 60-965, Poland; orcid.org/0000-0002-4364-8267; Phone: +48 616653681; Email: michal.niemczak@put.poznan.pl;

Julia L. Shamshina, Fiber and Biopolymer Research Institute, Department of Plant and Soil Science, Texas Tech University, Lubbock, TX, USA; orcid.org/ 0000-0003-0708-764X; Phone: +1 806 834-8055; Email: jshamshi@ttu.edu

ESI

Table of Contents:

1. Abbreviations and symbols **S3**

2. Synthesis of choline-type esterquats **S3-S28**
2.1. Synthesis via Fischer esterification of aminoalcohols S3-S8
2.2. Synthesis via acylation and subsequent quaternization of aminoalcohols S8-S11
2.3. Synthesis using other derivatives of carboxylic acids S11-S16
2.4. Synthesis of choline-based esterquats from biologically active compounds S17-S20
Table S1. Syntheses of choline esters S21-S28

3. Synthesis of betaine-type esterquats **S29-S49**
3.1. Synthesis from haloacyl halides - esterquats S29-S32
3.2. Synthesis from haloesters- esterquats S32-S37
3.3. Synthesis from chlorobetainyl chloride - esterquats S37-S38
3.4. Synthesis of glycine betaine by classic esterification - esterquats S38-S41
3.5. Synthesis by O-alkylation of betaines esterquats - esterquats S41-S42
3.6. Synthesis by anion exchange – esterquats S42
Table S2. Syntheses of betaine-type esterquats S43-S49

4. Structures **S50-S62**
4.1. Structures of compounds (hydrolysis) S50
4.2. Structures of compounds (biodegradation) S51-S52
4.3. Structures of compounds (toxicity) S53-S57
4.4. Structures of compounds (surface properties) S58-S62

5. Table S3. Surface properties of esterquats **S63-69**

6. Bibliography **S70-74**

1. Abbreviations and symbols

Ala – Alanine
Bn – Benzyl group
Bz – Benzoyl group
CO – Carbonyl group
DMF – *N,N*-dimethylformamide
DMSO – Dimethyl sulfoxide
Et_2O – Diethyl ether
Et_3N – Triethylamine
Gly – Glycine
iPrOH – Isopropanol
MeOH – Methanol
Nle – Norleucine
Ph – Phenyl group
Phe – Phenylalanine
PMDTA – *N,N,N',N",N"*-pentamethyldiethylenetetramine
p-TsOH – Tosylic acid
THF – Tetrahydrofuran
Val – Valine
MCPA – Anion formed from 2-methyl-4-chlorophenoxyacetic acid
MCPP – Anion formed from 2-(2-methyl-4-chlorophenoxy)propionic acid

2. Synthesis of choline-type esterquats

2.1. Synthesis *via* Fischer esterification of aminoalcohols

One of the possible methods of synthesizing choline-type esterquats relies on the utilization of aminoalcohols, such as dimethylethanolamine (deanol), which is widely known as a direct precursor of naturally occurring choline (2-hydroxyethyltrimethylammonium) cations. The route toward esterquats starting from aminoalcohols is a classic, acid-catalyzed Fischer esterification, accompanied by vacuum-supported water removal, followed by direct alkylation.[1–10]

In 1998, Chung and Park prepared esterquats through the esterification of triethanolamine **1** with palmitic acid (**Figure S1** and **Table S1**, No. 1-4). In this method, the reagents were mixed with the addition of a catalytic amount of concentrated sulfuric acid. Then, the products **E1**, **E2** and **E3** ($R_1 = C_{15}H_{31}$) were dissolved in isopropanol and mixed with dimethyl sulfate (added dropwise). After the reaction, the products **C1**, **C2** and **C3** were purified by HPLC with a mixture of *n*-propanol and water (1:1 v/v) as the eluent.[1] A few years later, Steiger and Friedli in 2002 successfully combined triethanolamine **1** with hydrogenated tallow fatty acids under a nitrogen atmosphere (**Figure S1**, **Table S1**, No. 5-8). It should be noted that in this approach, no catalyst was used, and the reaction occurred due to the high temperature, which facilitated the removal of the formed byproduct (water). Then, the products **E3** ($R_1 = C_{15}H_{31}$, $C_{17}H_{35}$) were quaternized by the slow addition of dimethyl sulfate in isopropanol to obtain the desired esterquats **C3**.[2] Gunjan and Tyagi in 2014 elaborated on a method that required the addition of triethanolamine **1** to melted fatty acids (**Figure S1** and **Table S1**, No. 9-12), showing a similarity to the method described by Steiger *et al.* However, Gunjan and Tyagi performed esterification in a vacuum, which in combination with the elevated temperature of the process resulted in more efficient removal of formed water. Analogous to previous protocols, the obtained esters **E2** ($R_1 = C_{15}H_{31}$, $C_{17}H_{33}$, $C_{17}H_{31}$) were quaternized using dimethyl sulfate to obtain appropriate esterquats **C2**.[3]

Interestingly, in 2015, Narayanan and Umapathy developed methods reported by Steiger et al. and Gunjan et al. and proposed mixing triethanolamine **1** along with respective acids (dodecannoic, tetradecanoic and hexadecanoic) and tosylic acid in a toluenic medium (**Figure S1** and **Table S1**, No. 13-16). After the reaction, the solvent was removed through vacuum distillation, and the products **E3** ($R_1 = C_{11}H_{23}$, $C_{13}H_{27}$, $C_{15}H_{31}$) were mixed with 1-bromooctane. The products **C4** were then isolated through vacuum distillation.[4]

The research was still being continued, and in 2016, Umapathy et al. proposed to conduct an esterification of triethanolamine **1** with fatty acids in the presence of tosylic acid as a catalyst

(**Figure S1** and **Table S1**, No. 17-20). The reaction was carried out in a Dean-Stark apparatus, with toluene as a solvent. Then, the solvent was evaporated to obtain ester **E3** ($R_1 = C_{11}H_{23}$, $C_{13}H_{27}$, $C_{15}H_{31}$). Alkylation was then carried out with epichlorohydrin to obtain esterquats **C5**.[5]

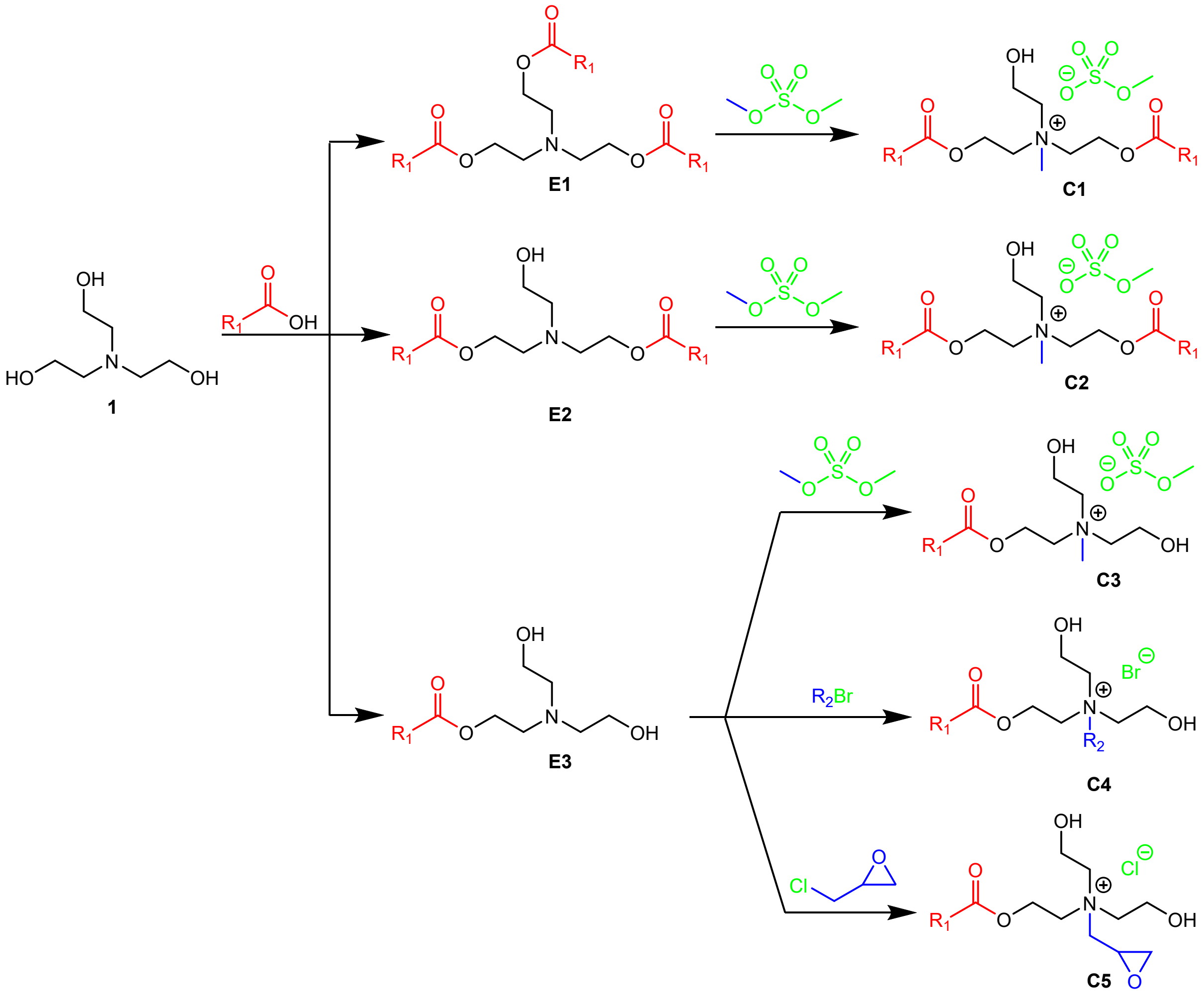

Figure S1. Synthesis of choline-type esterquats by esterification of triethanolamine and subsequent quaternization of the obtained semiproducts (more details are available in **Table S1**, No. 1-20).

In 2000, Tatsumi et al. proposed a similar method to that of Chung et al., which was based on mixing deanol and its butyl or hexyl homologues (2, n = 1, 3 or 5) with octanoic, decanoic, dodecanoic, tetradecanoic and hexadecanoic acids in xylene with catalytic amounts of tosylic acid (**Figure S2** and **Table S1**, No. 21-28). The main difference between the mentioned methodologies is in the selection of catalyst (Chung et al. used concentrated sulfuric acid). In the method described by Tatsumi et al., the solvent and excess dimethylaminoalcohol were evaporated after the reaction, and then the products **E4** ($R_1 = C_7H_{15}$, C_9H_{19}, $C_{11}H_{23}$, $C_{13}H_{27}$, $C_{15}H_{31}$) were purified by fractional distillation. Subsequently, **E4** was mixed with hydrogen

chloride and epichlorohydrin in isopropanol. The solvent was then evaporated, and the crude products **C6** (R_2 = CH-OH), X = Cl) were purified chromatographically with a mixture of chloroform and methanol (9:1 v/v). Tatsumi et al. also performed quaternization with chloromethane instead of epichlorohydrin, resulting from incorporation of its fumes into isopropanolic solution of product **E4**. Then, the solvent and remaining chloromethane were evaporated, and the crude product **C7** was recrystallized from a mixture of acetone and isopropanol (19:1 v/v).[6] Tatsumi et al. continued their research, and the next year, they proposed quaternization of **E4** with 1,4-dichlorobutane, 1,4-dichloro-2-butene (both *cis* and *trans* isomers), and 1,4-dichloro-2-butyne (**Figure S2** and **Table S1**, No. 29-39). The reagents were mixed in isopropanol, and after the reaction, the solvent was evaporated. The obtained products **C6** (R_2 = C_2H_4, *trans*-CH=CH, *cis*-CH=CH, C≡C, X = Cl) were then washed with hexane and recrystallized from a mixture of acetone and isopropanol (9:1 v/v).[7]

In 2011, Xu et al. proposed the synthesis of nonsymmetric gemini surfactants *via* esterification of deanol **2 (n = 1)** with octanoic, decanoic, dodecanoic, tetradecanoic and hexadecanoic acids in the presence of tosylic acid as a catalyst (**Figure S2** and **Table S1**, No. 40-46). The products **E4** (R_1 = C_7H_{15}, C_9H_{19}, $C_{11}H_{23}$, $C_{13}H_{27}$, $C_{15}H_{31}$) in this new method proposed by Xu et al. were then washed with diethyl ether, and the residual reagents were evaporated. The next step was to add **E4 (R_1 = $C_{11}H_{23}$)** into a methanolic solution of epichlorohydrin and hydrogen chloride. After the reaction, the solvent and unreacted epichlorohydrin were evaporated, and then the crude product **C8** was washed with a cool mixture of hexane and ethyl acetate and recrystallized multiple times from a mixture of acetone and methanol. Then, the reaction between the products of both reactions (**E4** and **C8**) in isopropanol was performed. Finally, the postreaction mixture was cooled, and the precipitate was filtered off and thoroughly washed to obtain esterquats **C6'** (R_1 = $C_{11}H_{23}$, R_2 = CH-OH, R_3 = C_7H_{15}, C_9H_{19}, $C_{11}H_{23}$, $C_{13}H_{27}$, $C_{15}H_{31}$).[8]

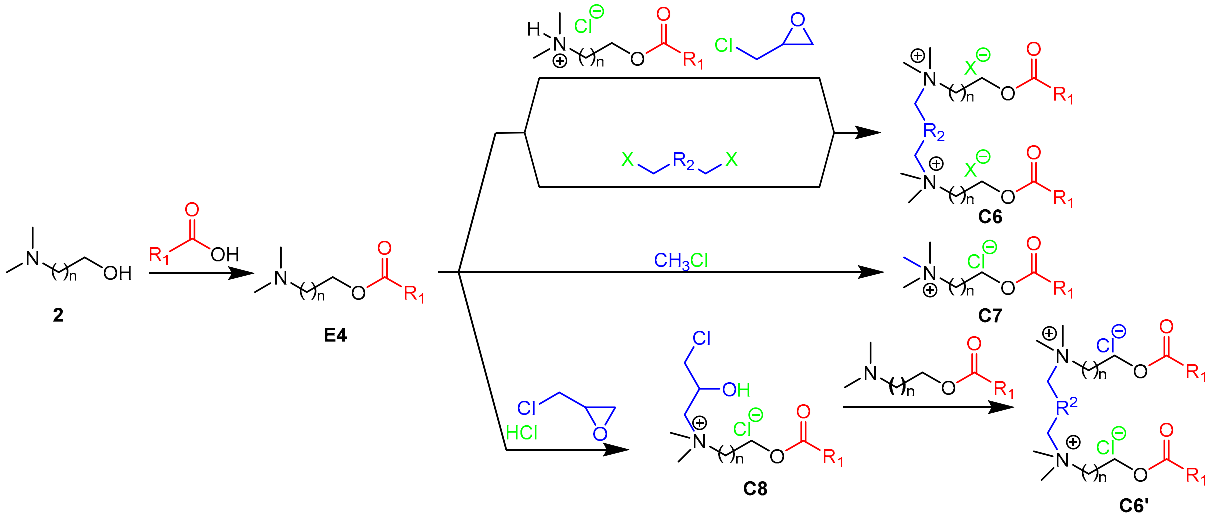

Figure S2. Synthesis of choline esters *via* esterification of deanol followed by quaternization (more details are available in **Table S1**, No. 21-46).

An interesting alternative for quaternization with the use of classic alkylating agents is the idea of carrying out the reaction with the use of oxirane.[9] This method, proposed by Han et al. in 2015, was performed starting from melted diethanolmethylamine **3** and appropriate fatty acids in the presence of catalytic amounts of phosphorous acid (**Figure S3** and **Table S1**, No. 47-51). The obtained ester **E5** (R_1 = C_7H_{15}, C_9H_{19}, $C_{11}H_{23}$) was converted into hydrochloride **E6** in isopropanol, and then oxirane was added, which led to insertion of a hydroxyethyl group (**C9**).[9]

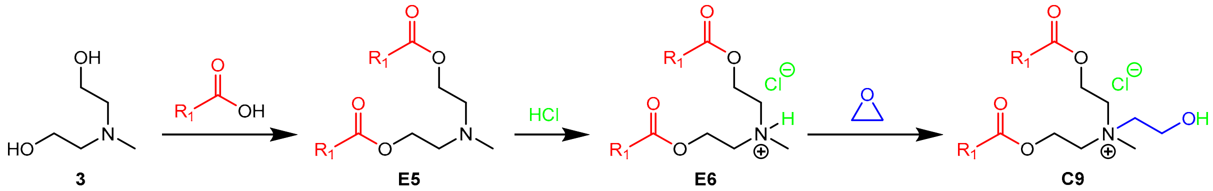

Figure S3. Synthesis of choline-type esterquats *via* quaternization with oxirane (more details are available in **Table S1**, No. 47-51).

Interestingly, ester **E5** was also used in the synthesis of diethyloxyester dimethylammonium chloride **C10** (**Figure S4**, industrial abbreviation DEEDMAC, **Table S1**, No. 52), which is one of the most commonly used surfactants from the esterquats family.[11] Unfortunately, this synthesis, despite being very important from an industrial point of view, has not been described in any scientific article thus far.

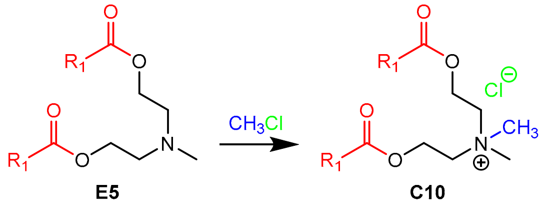

Figure S4. Industrial synthesis of choline-type esterquats via methylation of **E5** (more details are available in **Table S1**, No. 52).

In 2016, Gao et al. suggested a different idea for the synthesis of choline-type esterquats. In this approach, acetonic solutions of the respective diacids (succinic or adipic; **Figure S5** and **Table S1**, No. 53-62), ethylene chlorohydrin **4** and concentrated sulfuric acid were mixed together. After the reaction, the postreaction mixture was neutralized with sodium hydrogen carbonate solution, and the solvent was evaporated. The products **E7** ($R_1 = C_2H_4$, C_4H_8) were then washed with brine, dissolved in dichloromethane and dried. The next step was based on the dissolution of **E7** and the respective dimethylalkylamines in ethyl acetate to initiate quaternization. Afterward, the solvent was evaporated, and the crude products **C11** ($R_2 = C_{10}H_{21}$, $C_{12}H_{25}$, $C_{14}H_{29}$, $C_{16}H_{33}$) were washed with petroleum ether or diethyl ether and then recrystallized from ethyl acetate and dried.[10]

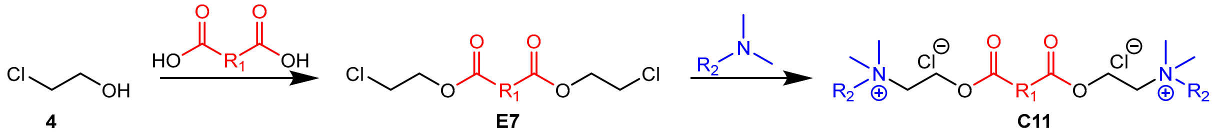

Figure S5. Synthesis of choline-type esterquats from ethylene chlorohydrin (more details are available in **Table S1**, No. 53-62).

The possibility of creating choline esters without the quaternization step is also present. Fahri et al. in 2020 described the method based on esterification of choline chloride (**5**) suspended in methanesulfonic acid, with excess hexanoic or octanoic acid (Figure S6, Table S1, No. 63-64). The reaction was performed under reduced pressure to remove by-forming water. Subsequently, the reaction mixture containing crude product **C7** was cooled under normal pressure, washed with diethyl ether and dichloromethane, and then dissolved in water to perform further anion exchange reactions.[12]

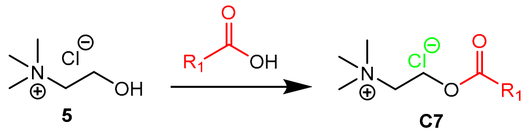

Figure S6. Synthesis of choline-type esterquats from choline hydrochloride (more details are available in **Table S1**, No. 63-64).

2.2. Synthesis *via* acylation and subsequent quaternization of aminoalcohols

Another method of synthesis of choline-based esterquats is acylation with the use of acid halides, originating mainly from fatty acids. The acylation is then followed by quaternization of the obtained esters with respective haloalkanes, such as bromomethane, iodomethane or halogenated derivatives of other compounds.[13–21]

In 1976, Bregadze et al. reported a procedure demonstrating the acylation of deanol **2** (n = 1, Y = H) dissolved in absolute ether with the addition of trimethylamine. The reaction was realized by the slow addition of an absolute etheric solution of the respective phenyl-*ortho*- or *meta*-carboranecarbonyl chloride to a solution of **2** (**Figure S7** and **Table S1**, No. 65-67). After the reaction, the precipitated byproduct was filtered off, and then the remaining filtrate was washed with water and dried over anhydrous magnesium sulfate, and the solvent was evaporated. Next, the obtained crude product **E4** (R_1 = C_6H_5-*o*-$CB_{10}H_{10}C$-, *m*-$HB_{10}H_{10}C$-) was dissolved in hexane and frozen out of solution to separate impurities. The purified product **E4** was subjected to reaction with a slight stoichiometric excess of iodomethane, which served as a quaternizing agent. Afterward, product **C12** was filtered off, washed with hexane and then with diethyl ether, and finally dried over phosphorus pentoxide.[15]

In 2007, Tehrani-Bagha et al. proposed a different method based on the dropwise addition of deanol **2** (n = 1, Y = H) into dodecanoyl chloride, both dissolved in dichloromethane (**Figure S7** and **Table S1**, No. 68-670). However, this method required removal of the byproduct of the side reaction between deanol and the solvent, which was washed away with the use of sodium hydrogen carbonate solution. The obtained aminoester **E4** (R_1 = $C_{11}H_{23}$) was then quaternized with bubbling of gaseous bromomethane or with an acetonic solution of 1,3-dibromopropane. The final products **C13** and **C6** (X = Br) were then purified by recrystallization from isopropanol or from a mixture of ethanol and diethyl ether.[13]

Węgrzyńska et al. in 2007 reported a method of acylation of deanol as well as its propyl and isopropyl homologues **2** (n = 1, 2, Y = H or CH_3) with respective acid (decanoyl, lauroyl and myristoyl) chlorides in a mixture of diethyl ether and chloroform (**Figure S7** and **Table S1**, No. 71-80). The obtained products **E4** (R_1 = C_9H_{19}, $C_{11}H_{23}$, $C_{13}H_{27}$) in the form of hydrochloride salts were then separated and purified *via* recrystallization from a mixture of chloroform and hexane. The next step involved neutralization of the hydrochloride moiety with a 10% solution of sodium hydrogen carbonate, followed by extraction with diethyl ether. The organic layer was then dried over anhydrous magnesium sulfate and evaporated. Subsequently, **E4** was quaternized with symmetrical dichlorodihydroxyalkylamine in isopropanol and then evaporated from the postreaction mixture. Similar to Tehrani-Bagha's method, the crude

product **C14** (R_2 = C_5H_{11}, C_6H_{13}, C_8H_{17}) was washed with diethyl ether or hexane and recrystallized from a mixture of acetone and isopropanol.[16]

In 2011, Pernak et al. used a method similar to that of Tehrani-Bagha et al., namely, a chloroformic solution of deanol **2** (n = 1, Y = H) was mixed with the previously obtained acyl chloride of a popular herbicide (namely, 4-chloro-2-methylphenoxyacetic acid, MCPA) (**Figure S7** and **Table S1**, No. 81-83). The reaction was carried out in an ice bath, and the byproduct (hydrogen chloride) was quenched with the addition of trimethylamine (TEA). Free aminoester **E4** (R_1 = (*4*-Cl-*2*-CH_3)-C_6H_3-OCH_2)- was then mixed with hexane and quaternized with iodomethane to obtain esterquat **C12**, which was then precipitated from the postreaction mixture with ethyl acetate, isolated and dried.[17] Interestingly, Syguda et al. in 2018 used the procedure combined with protocols elaborated by Tehrani-Bagha et al. and Pernak et al. to obtain a group of functionalized esterquats. Their synthesis is discussed further in the section titled "Introduction of biological activity".

In 2016, Migahed et al. reported a procedure based on the acylation of the sodium-activated form of the deanol homologue 3-dimethylaminopropanol **2** (n = 2, Y = H). As shown in **Figure S7** (and **Table S1**, No. 84-93), this unique procedure was based on mixing 3-dimethylaminopropanol with sodium under a nitrogen atmosphere, followed by dropwise addition of the respective acyl chloride (decanoyl or dodecanoyl). The reaction was carried out in acetone, which allowed for facile removal of precipitated products **E4** (R_1 = C_9H_{19}, $C_{11}H_{23}$) that were subsequently purified by recrystallization from diethyl ether. Then, the obtained esters were quaternized through a linker obtained by merging the respective primary amine (butyl-, pentyl-, hexyl- or octylamine) with epichlorohydrin. The reactions were carried out in acetone, which was further removed by evaporation. The products **C14** (R_2 = C_4H_9, C_5H_{11}, C_6H_{13}, C_8H_{17}) were then purified by recrystallization from acetone, followed by washing with diethyl ether and vacuum drying.[18]

In 2016, Para et al. proposed a method (**Figure S7** and **Table S1**, No. 94-96) starting from the addition of a chloroformic solution of dodecanoyl chloride into an etheric solution of deanol or *N*,*N*-dimethyl-1-methylethanol **2** (n = 1 or 2, Y = H or CH_3). After the reaction, the precipitate was separated and washed with diethyl ether. Dried crude hydrochloride of product **E4** (R_1 = $C_{11}H_{23}$) was then purified by recrystallization from a mixture of hexane and chloroform (3:2 v/v). Subsequent addition of 5% sodium hydrogen carbonate solution (alkalization), followed by extraction with diethyl ether, enabled them to obtain free amine. Then, the solvent was evaporated, and bromomethane was added. Finally, the obtained precipitate **C13** (R_2 = CH_3) was washed with diethyl ether and recrystallized from acetonitrile.[14]

The other method reported by Liu et al. in 2020 starts by mixing a dichloromethanoic solution of previously obtained palmitoyl chloride or myristoyl chloride with a mixture of deanol (2, n = 1, Y = H) and triethylamine (**Figure S7** and **Table S1**, No. 97-99). Then, product **E4** (R_1 = $C_{13}H_{27}$, $C_{15}H_{31}$) was filtered off, washed and purified *via* vacuum distillation. **E4** was then subjected to reaction with dibromobutane in isopropanol. Next, the solvent was evaporated, and the product **C6** (R_2 = C_2H_4, X = Br) was recrystallized from a mixture of ethyl acetate and isopropanol (1:1 v/v) and then dried under reduced pressure.[21]

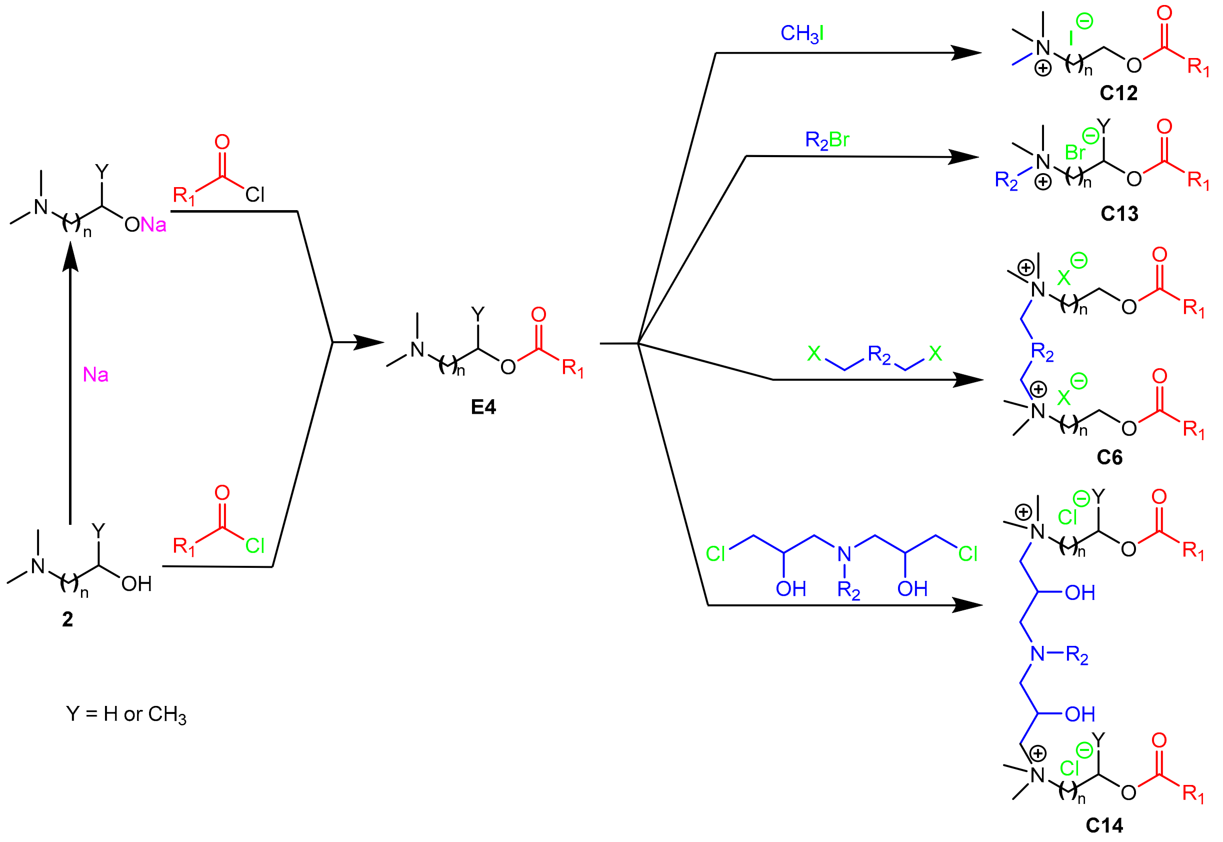

Figure S7. Synthesis of choline esters *via* acylation and subsequent quaternization of deanol (more details are available in **Table S1**, No. 65-99).

2.3. Synthesis using other derivatives of carboxylic acids

A literature survey indicates that there are also miscellaneous ways of synthesizing choline-based esterquats, which rely on the use of other carboxylic acid derivatives, such as acid anhydrides, esters, and salts, as well as enzymes.[22–31]

As presented in **Figure S8** (and **Table S1**, No. 100-103), Tammelin in 1956 described a method of esterification of deanol **2** (n = 1) with the respective acid (acetic, propionic or butyric)

anhydride. The reaction was performed without the solvent in a water bath and was followed by reduced-pressure distillation. Next, the obtained products **E4** (R_1 = CH_3, C_2H_5, C_3H_7) were dissolved in diethyl ether, and iodomethane was added to initiate quaternization. The obtained precipitates of **C12** were filtered off and then dried.[24]

Several methods, described in 1983 by Topuzyan et al., involve esterification of deanol **2** (n = 1) with imidazole amides, mixed acid anhydrides and activated esters followed by direct *O*-alkylation of salts (**Figure S8**, **Table S1**, No. 104-107). In the first method, the precursor was synthesized *via* dropwise addition of deanol **2** into a tetrahydrofuranoic solution obtained previously from *N*-benzyloxycarbonylglycine imidazolide (imidazole amide). Then, the solvent was distilled off, and the crude product **E4** (R_1 = $C_6H_5CH_2OC(=O)NHCH_2$-) was dissolved in ethyl acetate, washed with a solution containing 5% sodium hydrogen carbonate and a saturated solution of sodium chloride, and finally dried. The second method starts with the addition of deanol **2** into a tetrahydrofuranoic solution of *N*-benzyloxycarbonylglycine – dichlorophosphoric anhydride. The solvent was distilled off after the reaction, and a saturated solution of potassium hydroxide was added. The next step involved extraction of the product with ethyl acetate. Then, the organic layer was washed with a saturated solution of sodium chloride and dried to obtain ester **E4**. The third method is based on the addition of deanol **2** into a chloroformic solution of *N*-benzyloxycarbonylglycine 4-nitrophenyl ester. The postreaction mixture was diluted with another portion of chloroform, washed with 5% calcium carbonate and with water, and finally dried. Independent of the selection of the method of esterification, Topuzyan et al. performed the subsequent quaternization reaction with iodomethane. For that purpose, previously obtained ester **E4** was dissolved in dry ethanol, and then iodomethane was added. The precipitated product **C12** was isolated, washed with diethyl ether and then dried.[25]

Hellberg in 2002 proposed a process based on the esterification of deanol **2** (n = 1) with triethyl orthoformate in the presence of citric acid as a catalyst and under slight vacuum (**Figure S8** and **Table S1**, No. 108-109). Additionally, it was possible to add more citric acid along with the respective alcohol (2-ethylhexanol, octanol, decanol, dodecanol, tetradecanol, or hexadecanol) to force exchange of the remaining ethyl groups into other alkyl groups. The obtained *ortho*-esteramine **E8** (R_1 = $C_5H_{11}(C_2H_5)CH_2$, C_8H_{17}, $C_{10}H_{21}$, $C_{12}H_{25}$, $C_{14}H_{29}$, $C_{16}H_{33}$, m = 0, 1, 2, 3, o = 0, 1, 2, 3, p = 0, 1, 2, 3) was mixed with isopropanol and sodium hydrogen carbonate, and after purging the mixture with nitrogen, chloromethane was added stepwise to obtain *ortho*-esterquat **C16**, which was subsequently isolated.[26]

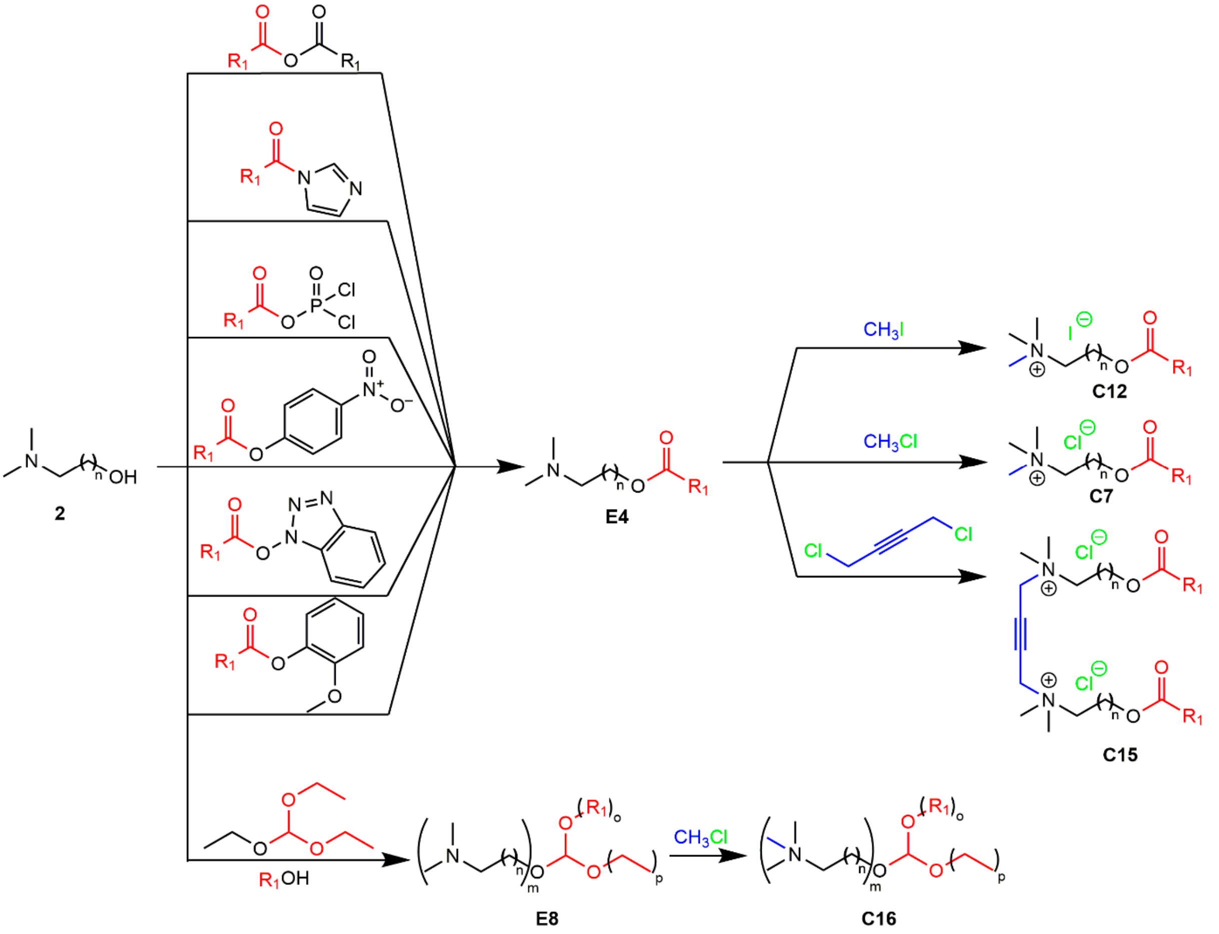

Figure S8. Synthesis of choline esters using various carboxylic acid derivatives (more details are available in **Table S1**, No. 100-138).

Banno et al. in 2007 described a method regarding the utilization of aromatic carbonate esters (**Figure S8** and **Table S1**, No. 110-119). The method was later adapted by Gilbert et al.[29] Authors added dimethylaminopropanol **2** (n = 2) or dimethylaminoisopropanol **2** (n = *iso*-2) to a mixture of trimethylamine and previously obtained solution containing carbonate esters of guaiacol and alcohols (decanol, dodecanol, tetradecanol and hexadecanol). After the reaction, the volatiles were evaporated, and the products **E4** ($R_1 = OC_9H_{19}$, $OC_{11}H_{23}$, $OC_{13}H_{27}$, $OC_{15}H_{31}$) were purified chromatographically with a mixture of chloroform and methanol (15:1 v/v). **E4** was then quaternized with iodomethane dissolved in dry chloroform. The solvent and remaining iodomethane were evaporated, and the obtained precipitate of **C12** was washed with ethyl acetate and then recrystallized from ethyl acetate.[27]

In 2014, Topuzyan et al. suggested the method of esterification of deanol or its propyl homologue **2** (n = 1 or 2) with previously obtained benzotriazole esters of amino acids (glycine, alanine, norleucine, phenylalanine and valine) substituted with benzoyl, 4-methoxybenzoyl or phthalimide (**Figure S8** and **Table S1**, No. 1209-128). First, the reagents were mixed with

acetonitrile, which was further evaporated. Crude products **E4** (R$_1$ = $C_6H_5CONHCH_2$, $C_6H_5CONHCH_2(CH_3)$, $C_6H_5CONHCH_2(C_4H_9)$, $C_6H_5CONHCH_2(CH_2C_6H_5)$, (*4*-$OCH_3$) $C_6H_5CONHCH_2(CH_2C_6H_5)$, $PhtNCH_2$, $PhtNCH_2[CH(CH_3)_2]$) were dissolved in acetonitrile and washed with a 2% solution of potassium hydroxide and then with pure water. The solution was then dried over sodium sulfate, and the solvent was evaporated. The next step involved the addition of iodomethane to acetonic solutions of product **E4**. The obtained precipitate of **C12** was then filtered off and recrystallized from ethanol.[28]

Gilbert et al. in 2021 described a method regarding the utilization of aromatic carbonate esters (**Figure S8** and **Table S1**, No. 129-138). The method was adapted from Banno et al. and appropriately modified.[27] The authors added deanol **2 (n = 1)** to a mixture of trimethylamine and previously obtained solution containing carbonate esters of guaiacol and alcohols (octanol, decanol, dodecanol, tetradecanol and hexadecanol). After the reaction, the volatiles were evaporated, and the products **E4** (R$_1$ = OC_8H_{17}, $OC_{10}H_{21}$, $OC_{12}H_{25}$, $OC_{14}H_{29}$ or $OC_{16}H_{33}$) were quaternized with 1,4-dichloro-2-butyne dissolved in isopropanol. The solvent was evaporated, and the obtained precipitate of **C15** was washed with ethyl acetate and then recrystallized.[29]

Topuzyan et al. in their work from 1983 performed alkylation with the use of an analogue of deanol - chloroethyldimethylamine **5** (**Figure S9** and **Table S1**, No. 139-144). Initially, **6** was added to a suspension of ethyl acetate and potassium 2-((4-ethoxy-4-oxobut-2-en-2-yl)amino)acetate or its propionate and butyrate homologues. The sediment was then filtered off, and the filtrate was washed with a saturated solution of sodium chloride. The next step involved drying over sodium sulfate and solvent removal. The product **E4** (R$_1$ = $C_2H_5OC(=O)CH=C(CH_3)NCH_2$, $C_2H_5OC(=O)CH=C(CH_3)NC_2H_4$, $C_2H_5OC(=O)CH=C(CH_3)NC_3H_6$) was then dissolved in diethyl ether and quaternized with iodomethane. The obtained precipitate of **C12** was filtered off, washed with diethyl ether and recrystallized from ethanol.[25]

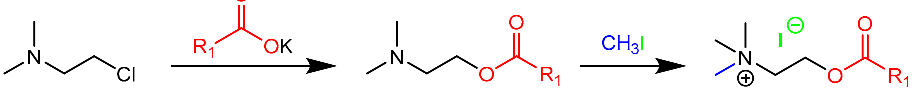

Figure S9. Synthesis of choline esters *via* *O*-alkylation of carboxylate salt and subsequent quaternization (more details are available in **Table S1**, No. 139-144).

Using enzymes is an interesting approach for performing catalytic reactions, as demonstrated by Lozano et al. in 2003, who utilized immobilized *Candida actarctica* lipase B to enhance the transesterification leading to choline and carnitine esters (**Figure S10** and **Table S1**, No. 145-

146). First, vinyl butyrate solutions in 2-methyl-2-butanol or acetonitrile were added to carnitine **7** and choline **5,** respectively, and then, after shaking, the immobilized enzyme was added. The products **C7** and **C17** (R_1 = C_3H_7) were isolated by centrifugation followed by HPLC.[22]

As is presented in **Figure S10** (and **Table S1**, No. 147), Mouterde et al. described in 2020 the ring-opening esterification of choline chloride **5** with Meldrum's acid (2,2-dimethyl-1,3-dioxane-4,6-dione), which is in fact one of the steps in the more complex synthesis of alkaloid analogs called sinapine. The goal of Mouterde et al. was to obtain a monoester of malonic acid **C18 via a simple synthesis pathway**. Due to the relatively high reactivity of both carboxylic groups, the appropriate precursor of the acid had to be used. Mouterde et al. mixed choline chloride and Meldrum's acid in acetonitrile, and after the reaction, the solvent and byproduct were evaporated to obtain monoester **C18**.[23]

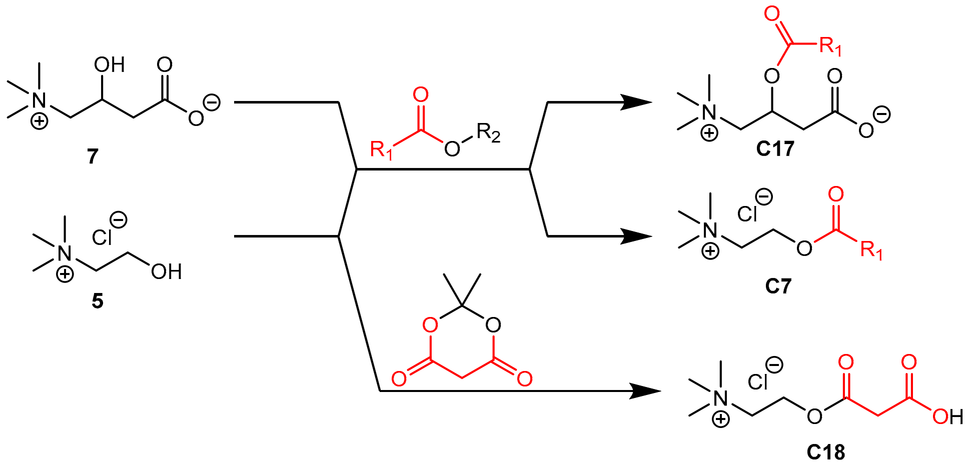

Figure S10. Synthesis of choline esters choline-type esterquats by transesterification (more details are available in **Table S1**, No. 145-147).

In 2009, Banno et al. proposed the idea of synthesis of gemini esterquats by utilization of carbonate esters instead of alkanoic esters (**Figure S11** and **Table S1**, No. 148-155) [30]. The procedure was based on mixing diphenyl carbonate, potassium carbonate as a catalyst and 2-iodoethanol or 3-iodopropanol **8** (n = 1 or 2, Y = H) in acetone or THF, respectively. In the next step, the catalyst was filtered off, and the solvent was evaporated. The obtained crude product **E9** was then purified *via* chromatographic methods with a mixture of hexane and chloroform (2:3 v/v) as the eluent. The products **E9** were then dissolved in ethyl acetate with decyl-, dodecyl- or tetradecyldimethylamine. In the final step, the solvent was evaporated, and the products **C19** (R_1 = $C_{10}H_{21}$, $C_{12}H_{25}$, $C_{14}H_{29}$) were purified by dissolution in chloroform, followed by precipitation with ethyl acetate.[30]

Banno et al. continued this research direction and in 2010 they presented another method utilizing diphenyl carbonate to obtain gemini esterquats (**Figure S11** and **Table S1**, No. 156-167), which was based on their earlier report from 2007.[27] In this manner, diphenyl carbonate was mixed with respective unsubstituted alcohols (octanol, decanol and dodecanol) and subsequently with *N*,*N*-dimethylaminoalcohols (propanol or isopropanol) **2** (n = 1, 2, Y = H or CH_3), creating asymmetric carbonate esters **E10** (R_1 = C_8H_{17}, $C_{10}H_{21}$, $C_{12}H_{25}$) in a one-pot manner. The reaction sequence was performed in the presence of trimethylamine as a catalyst, which was then removed by evaporation. The crude products **E10** were purified chromatographically using a mixture of chloroform and methanol (15:1 v/v). The products **E10** were then quaternized either with 1,3-diiodopropane, methyl iodide or **E9** (m = 1, 2) in dry acetonitrile to obtain products **C20** (R_2 = CH_2), **C21** and **C22**, respectively. The solvent was then evaporated, and the crude products were recrystallized from ethyl acetate.[31]

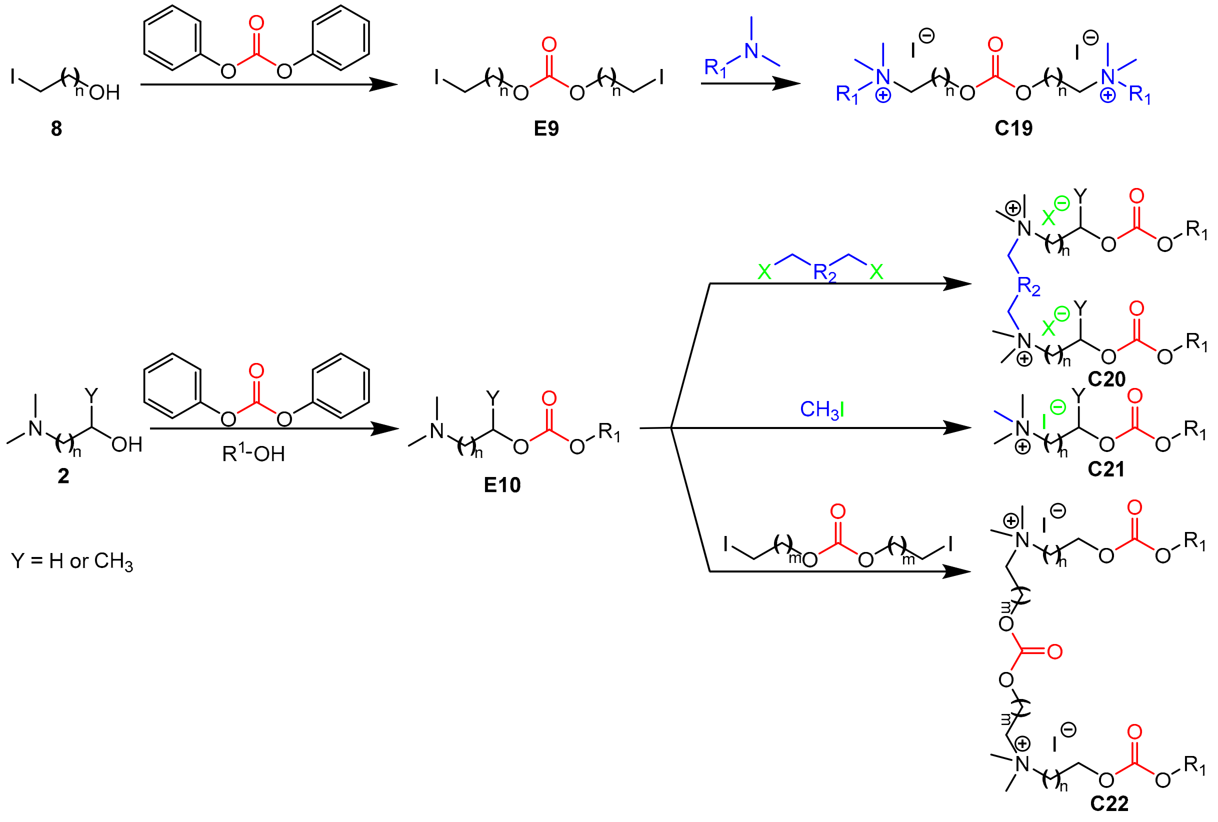

Figure S11. Synthesis of choline esters by carbonate transesterification (more details are available in **Table S1**, No. 148-167).

2.4. Synthesis of choline-based esterquats from biologically active compounds

Contrarily, the approach based on the anion exchange reaction, presented in **Figure S12**, can be efficiently carried out in short-chain alcohols, ethanol or methanol, as well as in water.[32] In the case of alcohols, being the most frequently used, fast dissolution of substrates causes a simultaneous precipitation of inorganic salts, such as sodium bromide or potassium chloride. After filtration of the byproduct, the solvent is evaporated, and the product **C24** is dried. For products exhibiting greater hydrophobicity, water can also be used as the reaction medium with satisfactory results.[20] The product **C24** is then separated from inorganic salts and water by two-phase extraction with the use of chloroform or dichloromethane. Usually, in the case of traditional QACs, these reactions are fast and can be performed within minutes; however, there are some exceptions, such as synthesis of denatonium benzoate.[32]

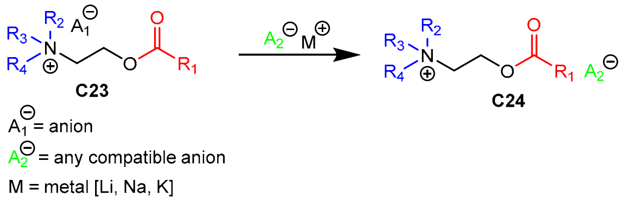

Figure S12. Synthesis of choline esterquats *via* anion exchange.

In 2018, Syguda et al. used a procedure combined from protocols by Tehrani-Bagha et al. and Pernak et al. Deanol **2** (n = 1) after dissolution in chloroform was added dropwise into acid chloride of MCPA and dissolved in chloroform as well. The rest of the procedure leading to the synthesis of esterquat **C12'** (R_1 = (*4*-Cl-*2*-CH_3)$C_6H_3OCH_2$, R_2 = $C_{10}H_{21}$) was analogous to that of Pernak et al. (**Figure S13** and **Table S1**, No. 168-1708); however, in this case, the quaternizing agent was added after dissolution in the same solvent in which the reaction was carried out (acetone).[19]

In a report from 2020, a chloroformic solution of previously obtained dicamba (3,6-dichloro-2-methoxybenzoic acid) acyl chloride was mixed with deanol **2** (n = 1), which was dissolved in the same solvent (**Figure S13** and **Table S1**, No. 171-174). When the reaction finished, the solvent was evaporated, and the residue was leached by hexane and cooled. The precipitated sediment was filtered off, washed with hexane and dried under vacuum. Then, the product was dissolved in chloroform, and a solution of triethylamine in chloroform was added to neutralize the formed hydrochloride. After the reaction and isolation of **E4"** (R_1 = (*3,6*-Cl-*2*-OCH_3), R_2 = $C_{10}H_{21}$, $C_{12}H_{25}$, $C_{14}H_{29}$), the aminoester was dissolved in acetone and mixed with excess decyl,

dodecyl or tetradecyl bromide. The crude products **C12"** were finally obtained by freeze crystallization and then purified by filtration, washed with hexane and acetone and dried.[20]

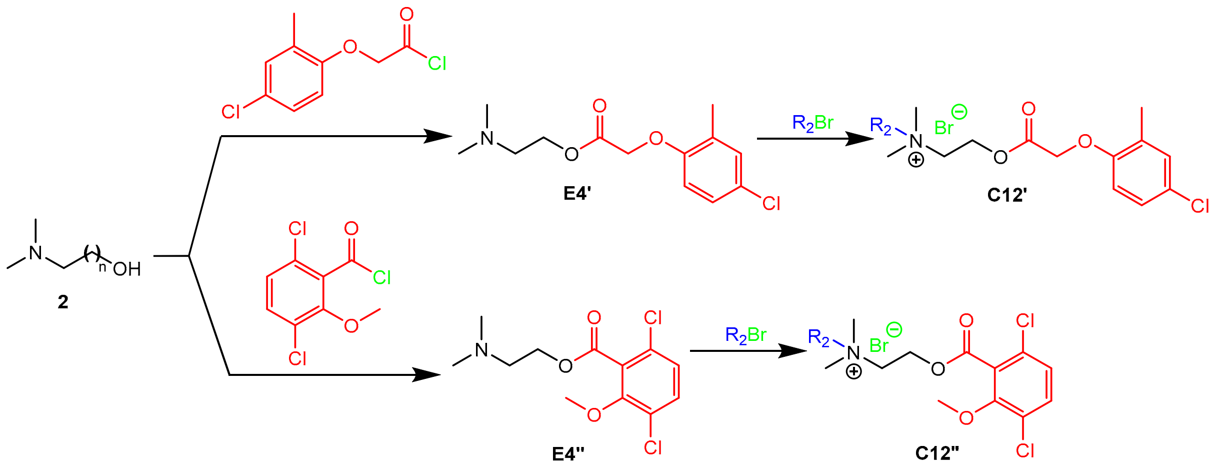

Figure S13. Synthesis of functionalized choline esterquats with biologically active acids (herbicides) in the cation (more details are available in **Table S1**, No. 168-174).

Esterquats with halogenate anions and biologically active cations (herbicides) such as **C12'** and **C12"** can be adjusted even more to their application requirement by the exchange of inert anions with herbicidally active anions, as demonstrated in **Figure S14**.[19,20]

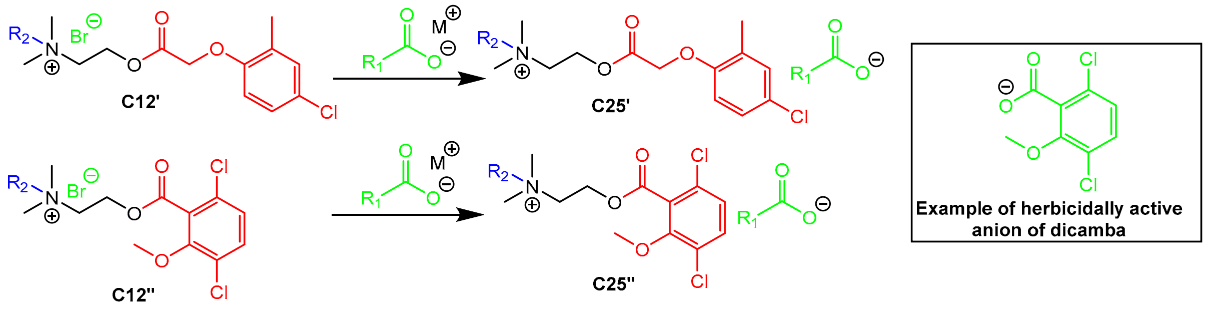

Figure S14. Synthesis of multifunctional choline esterquats *via* anion exchange.

The ability to build esterquats as the modifications of widely used drugs to enhance their pharmacological parameters has also been demonstrated recently. Incorporating amphiphilic drug moieties and specific spacer groups into gemini surfactants, often in an asymmetric way, can induce self-assembly of the molecules into bispherical structures. This property appears to ease the manner of entering the drug inside the cells, potentially giving better targeting and tissue penetration; therefore, the compound acts as a dual-functional moiety.[33]

Tang et al. proposed quite a different method based on the quaternization of lidocaine or its hydrochloride with 2-bromoethanol or 3-bromopropanol (8, n = 1 or 2) and subsequent acylation with various agents (**Figure S15** and **Table S1**, No. 175-184). The reagents were

heated for several hours and then mixed with ethyl acetate. The precipitated QASs **Q1** were filtered off, washed again with ethyl acetate, and dried under vacuum. Then, **Q1** was mixed with 1,2-dichloroethane and triphosgene as an acylating agent, and pyridine dissolved in 1,2-dichloroethane was added dropwise. The next step involved the addition of appropriate fatty alcohols (pentanol, heptanol, nonanol or dodecanol) as solutions in 1,2-dichloroethane to obtain esterquats **C26**. The reaction mixture was then washed with brine, and the solvent was evaporated. The obtained product **C26** was then purified chromatographically with a mixture of dichloromethane and methanol. Tang et al. also obtained a cyclohexyl analog of lidocaine **Q2** by alkylating 2-bromo-*N*-cyclohexylacetamide with deanol **2**, and analogous esterquat **C28** was synthesized using the same procedure. An alternative approach, proposed by Tang et al., uses acylation with classic acyl chloride, obtained from nonanoic acid and oxalyl chloride. A solution of nonanoyl chloride in dichloromethane was added dropwise into a dichloromethanoic solution of **Q1** and pyridine. The mixture was washed with brine, and dichloromethane was evaporated. Then, product **C27** was purified chromatographically with a mixture of dichloromethane and methanol (20:1 v/v).[33]

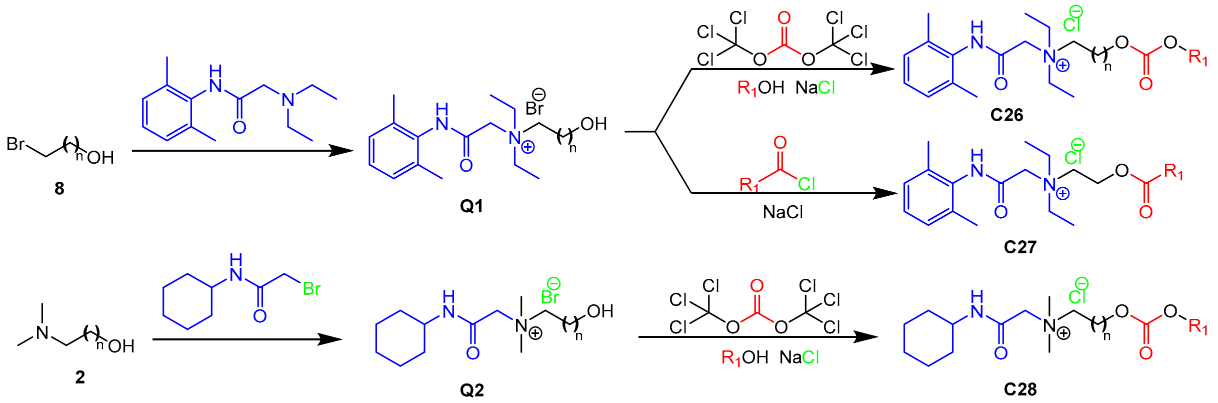

Figure S15. Synthesis of multifunctional choline esterquats *via* acylation of lidocaine drug and its analog (more details are available in **Table S1**, No. 175-184).

Interestingly, there is also a report demonstrating the utilization of esterquat **C18** in Knoevenagel-Doebner condensation in boiling ethanol to obtain sinapine and its analogues (**Figure S16** and **Table S1**, No. 185-188) with multifunctional (antimicrobial and antioxidant) properties. Mouterde et al. started with the synthesis of imine **9** using appropriate aldehydes (syringaldehyde for sinapine) and proline, which was later condensed with **C18** to obtain product **C30** with yields varying from 34 to 61%.[23]

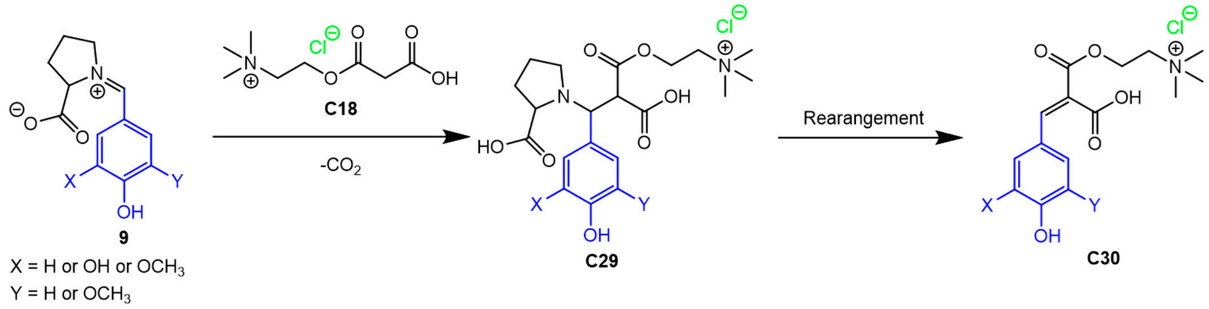

Figure S16. Synthesis of sinapine and its analogues *via* Knoevenagel-Doebner condensation (more details are available in **Table S1**, No. 185-188).

Table S1. Syntheses of choline esters.

No.	Stage	Substrate	R_1/R_2 or Reagent	Conditions	Product	Yield	Ref.
1	I	Triethanolamine (**1**)	$C_{15}H_{31}$	H_2SO_4, 190 °C, 4h	**E1, E2, E3**	NA	[1]
2	II	**E1**	Dimethyl sulfate	iPrOH, 60°C	**C1**	NA	
3	II	**E2**	Dimethyl sulfate	iPrOH, 60°	**C2**	NA	
4	II	**E3**	Dimethyl sulfate	iPrOH, 60°	**C3**	NA	
5	I	Triethanolamine (**1**)	$C_{13}H_{27}$	180 °C, N_2	**E3**	NA	[2]
6	I	Triethanolamine (**1**)	$C_{15}H_{31}$	180 °C, N_2	**E3**	NA	
7	I	Triethanolamine (**1**)	$C_{17}H_{35}$	180 °C, N_2	**E3**	NA	
8	II	**E3**	Dimethyl sulfate	iPrOH, 60-80 °C	**C3**	NA	
9	I	Triethanolamine (**1**)	$C_{15}H_{31}$	140 °C, 4 h	**E2**	NA	[3]
10	I	Triethanolamine (**1**)	$C_{17}H_{31}$	140 °C, 4 h	**E2**	NA	
11	I	Triethanolamine (**1**)	$C_{17}H_{33}$	140 °C, 4 h	**E2**	NA	
12	II	**E2**	Dimethyl sulfate	40 °C, 3h	**C2**	90%	
13	I	Triethanolamine (**1**)	$C_{11}H_{23}$	p-TsOH, Toluene, 140 °C, 24 h	**E3**	NA	[4]
14	I	Triethanolamine (**1**)	$C_{13}H_{27}$	p-TsOH, Toluene, 140 °C, 24 h	**E3**	NA	
15	I	Triethanolamine (**1**)	$C_{15}H_{31}$	p-TsOH, Toluene, 140 °C, 24 h	**E3**	NA	
16	II	**E3**	1-bromooctane	Reflux, 48 h	**C4**	NA	
17	I	Triethanolamine (**1**)	$C_{11}H_{23}$	p-TsOH, Toluene, 140 °C, 24 h	**E3**	NA	[5]
18	I	Triethanolamine (**1**)	$C_{13}H_{27}$	p-TsOH, Toluene, 140 °C, 24 h	**E3**	NA	
19	I	Triethanolamine (**1**)	$C_{15}H_{31}$	p-TsOH, Toluene, 140 °C, 24 h	**E3**	NA	
20	II	**E3**	Epichlorohydrin	35 °C, 24 h	**C5**	NA	
21	I	Deanol (**2**, n = 1)	C_9H_{19}	p-TsOH, reflux (~140 °C), 10 h	**E4**	83%	[6]
22	I	Deanol (**2**, n = 1)	$C_{11}H_{23}$	p-TsOH, reflux (~140 °C), 10 h	**E4**	86%	
23	I	Deanol (**2**, n = 1)	$C_{13}H_{27}$	p-TsOH, reflux (~140 °C), 10 h	**E4**	91%	
24	I	Deanol (**2**, n = 1)	$C_{15}H_{31}$	p-TsOH, reflux (~140 °C), 10 h	**E4**	86%	
25	I	Dimethylaminobutanol (**2**, n = 3)	C_9H_{19}	p-TsOH, reflux (~140 °C), 10 h	**E4**	82%	
26	I	Dimethylaminohexanol (**2**, n = 5)	C_7H_{15}	p-TsOH, reflux (~140 °C), 10 h	**E4**	81%	
27	II	**E4**	Epichlorohydrin	Product·HCl, reflux (82 °C), 72 h	**C6 (R_2 = CH-OH, X = Cl)**	39-68%	

28	II	**E4**	Chloromethane	iPrOH, 50 °C, 24 h	**C7**	80%	
29	II	**E4** (n = 1, $R_1 = C_{11}H_{23}$)	1,4-dichlorobutane	iPrOH, reflux (82 °C), 10 h	**C6**	30%	7
30	II	**E4** (n = 1, $R_1 = C_{11}H_{23}$)	*Cis*-1,4-dichloro-2-butene	iPrOH, (82 °C) reflux, 10 h	**C6**	90%	
31	II	**E4** (n = 1, $R_1 = C_9H_{19}$)	*Trans*-1,4-dichloro-2-butene	iPrOH, (82 °C) reflux, 10 h	**C6**	86^	
32	II	**E4** (n = 1, $R_1 = C_{11}H_{23}$)	*Trans*-1,4-dichloro-2-butene	iPrOH, (82 °C) reflux, 10 h	**C6**	84%	
33	II	**E4** (n = 1, $R_1 = C_{13}H_{27}$)	*Trans*-1,4-dichloro-2-butene	iPrOH, (82 °C) reflux, 10 h	**C6**	86%	
34	II	**E4** (n = 1, $R_1 = C_{15}H_{31}$)	*Trans*-1,4-dichloro-2-butene	iPrOH, (82 °C) reflux, 10 h	**C6**	80%	
35	II	**E4** (n = 1, $R_1 = C_{17}H_{35}$)	*Trans*-1,4-dichloro-2-butene	iPrOH, (82 °C) reflux, 10 h	**C6**	91%	
36	II	**E4** (n = 3, $R_1 = C_9H_{19}$)	*Trans*-1,4-dichloro-2-butene	iPrOH, (82 °C) reflux, 10 h	**C6**	82%	
37	II	**E4** (n = 5, $R_1 = C_7H_{15}$)	*Trans*-1,4-dichloro-2-butene	iPrOH, (82 °C) reflux, 10 h	**C6**	89%	
38	II	**E4** (n = 5, $R_1 = C_9H_{19}$)	*Trans*-1,4-dichloro-2-butene	iPrOH, (82 °C) reflux, 10 h	**C6**	88%	
39	II	**E4** (n = 1, $R_1 = C_{11}H_{23}$)	1,4-dichloro-2-butyne	iPrOH, (82 °C) reflux, 10 h	**C6**	84%	
40	I	Deanol (2, n = 1)	C_7H_{15}-	EtOH/$CHCl_3$, p-TsOH, 150 °C, 8-10 h	**E4**	NA	8
41	I	Deanol (2, n = 1)	C_9H_{19}-	EtOH/$CHCl_3$, p-TsOH, 150 °C, 8-10 h	**E4**	NA	
42	I	Deanol (2, n = 1)	$C_{11}H_{23}$-	EtOH/$CHCl_3$, p-TsOH, 150 °C, 8-10 h	**E4**	NA	
43	I	Deanol (2, n = 1)	$C_{13}H_{27}$-	EtOH/$CHCl_3$, p-TsOH, 150 °C, 8-10 h	**E4**	NA	
44	I	Deanol (2, n = 1)	$C_{15}H_{31}$-	EtOH/$CHCl_3$, p-TsOH, 150 °C, 8-10 h	**E4**	NA	
45	II	**E4**	Epichlorohydrin	MeOH, HCl, 25 °C, 28-30 h	**C8**	NA	
46	III	**C7**	**E_4**+**C_7**	iPrOH, 85 °C, 10-12 h	**C6'**	39%	
47	I	Methyldiethanolamine (**3**)	C_7H_{15}-	H_3PO_3, N_2, 190-200 °C	**E5**	NA	9
48	I	Methyldiethanolamine (**3**)	C_9H_{19}-	H_3PO_3, N_2, 190-200 °C	**E5**	NA	
49	I	Methyldiethanolamine (**3**)	$C_{11}H_{23}$-	H_3PO_3, N_2, 190-200 °C	**E5**	NA	
50	II	**E5**	HCl, H_2O	iPrOH, 20-40 °C, 30 min	**E6**	NA	
51	III	**E6**	Oxirane	40 °C, 1 h	**C9**	NA	
52	II	**E5**	CH_3Cl	NA	**C10**	NA	NA
53	I	Ethylene chlorohydrin (**4**)	-C_2H_4-	Acetone, H_2SO_4, 56 °C, 8 h	**E7**	NA	10

54	I	Ethylene chlorohydrin (**4**)	$-C_6H_{12}-$	Acetone, H_2SO_4, 56 °C, 8 h	**E7**	NA	
55	II	**E7** ($R_1 = C_2H_4$)	$C_{10}H_{21}-$	Ethyl acetate, 77 °C, 60 h	**C11**	32%	
56	II	**E7** ($R_1 = C_2H_4$)	$C_{12}H_{25}-$	Ethyl acetate, 77 °C, 60 h	**C11**	54%	
57	II	**E7** ($R_1 = C_2H_4$)	$C_{14}H_{29}-$	Ethyl acetate, 77 °C, 60 h	**C11**	67%	
58	II	**E7** ($R_1 = C_2H_4$)	$C_{16}H_{33}-$	Ethyl acetate, 77 °C, 60 h	**C11**	60%	
59	II	**E7** ($R_1 = C_4H_8$)	$C_{10}H_{21}-$	Ethyl acetate, 77 °C, 60 h	**C11**	30%	
60	II	**E7** ($R_1 = C_4H_8$)	$C_{12}H_{25}-$	Ethyl acetate, 77 °C, 60 h	**C11**	42%	
61	II	**E7** ($R_1 = C_4H_8$)	$C_{14}H_{29}-$	Ethyl acetate, 77 °C, 60 h	**C11**	62%	
62	II	**E7** ($R_1 = C_4H_8$)	$C_{16}H_{33}-$	Ethyl acetate, 77 °C, 60 h	**C11**	60%	
63	I	Choline chloride (**5**)	C_6H_{13}	Methanesulfonic acid, 90-100 °C, reduced pressure (50-100 mbar), 6 h	**C7**	80%	[12]
64	I	Choline chloride (**5**)	C_8H_{17}	Methanesulfonic acid, 90-100 °C, reduced pressure (50-100 mbar), 6 h	**C7**	88%	
65	I	Deanol (**2**, n = 1, Y = H)	$Ph-o-CB_{10}H_{10}C-$	Et_2O, Et_3N, 20 °C, 1h	**E4**	55%	[15]
66	I	Deanol (**2**, n = 1, Y = H)	$m-HB_{10}H_{10}C-$	Et_2O, Et_3N, 20 °C, 5-6h	**E4**	92%	
67	II	**E4**	Iodomethane	20 °C, 6-12 h	**C12**	97-98%	
68	I	Deanol (**2**, n = 1, Y = H)	$C_{11}H_{23}-$	CH_2Cl_2, 25 °C, 4 h, 5% $NaHCO_3$	**E4**	90%	[13]
69	II	**E4**	CH_3-	iPrOH, 50 °C, 2.5 h	**C13**	92%	
70	II	**E4**	$-CH_2-$	Acetone, 56 °C, 50 h	**C6**	92%	
71	I	Deanol (**2**, n = 1, Y = H)	$C_{11}H_{23}-$	$Et_2O/CHCl_3$, 10% $NaHCO_3$	**E4**	NA	[16]
72	I	Dimethylaminopropanol (**2,** n = 2, Y = H)	$C_9H_{19}-$	$Et_2O/CHCl_3$, 10% $NaHCO_3$	**E4**	NA	
73	I	Dimethylaminopropanol (**2,** n = 2, Y = H)	$C_{11}H_{23}-$	$Et_2O/CHCl_3$, 10% $NaHCO_3$	**E4**	NA	
74	I	Dimethylaminopropanol (**2,** n = 2, Y = H)	$C_{13}H_{27}-$	$Et_2O/CHCl_3$, 10% $NaHCO_3$	**E4**	NA	
75	I	Dimethylaminoisopropanol (**2**, n = 1, Y = CH_3)	$C_9H_{19}-$	$Et_2O/CHCl_3$, 10% $NaHCO_3$	**E4**	NA	
76	I	Dimethylaminoisopropanol (**2**, n =1, Y = CH_3)	$C_{11}H_{23}-$	$Et_2O/CHCl_3$, 10% $NaHCO_3$	**E4**	NA	
77	I	Dimethylaminoisopropanol (**2**, n =1, Y = CH_3)	$C_{13}H_{27}-$	$Et_2O/CHCl_3$, 10% $NaHCO_3$	**E4**	NA	

78	II	**E4**	C_5H_{11}-	iPrOH	**C14**	73-85%	
79	II	**E4**	C_6H_{13}-	iPrOH	**C14**	56-85%	
80	II	**E4**	C_8H_{17}-	iPrOH	**C14**	61-83%	
81	I	Deanol (**2**, n = 1, Y = H)	(*2*-CH_3-*4*-Cl)Ph-O-CH_3-	$CHCl_3$, 4 °C (ice bath), 10 min	**E4***HCl	98%	17
82	II	**E4***HCl	Et_3N	$CHCl_3$, 25 °C	**E4**	82%	
83	III	**E4** (82%)	CH_3I	Hexane, 25 °C, 1 h	**C12**	63%	
84	I	Dimethylaminopropanol (**2**, n = 2, Y = H)	C_9H_{19}-	Na, acetone, N_2, 56 °C, 6h	**E4**	86%	18
85	I	Dimethylaminopropanol (**2**, n = 2, Y = H)	$C_{11}H_{23}$-	Na, acetone, N_2, 56 °C, 6h	**E4**	89%	
86	II	**E4** (R_1 = C_9H_{19})	C_4H_9-	Acetone, 56 °C, 12 h	**C14**	65%	
87	II	**E4** (R_1 = $C_{11}H_{23}$)	C_4H_9-	Acetone, 56 °C, 12 h	**C14**	72%	
88	II	**E4** (R_1 = C_9H_{19})	C_5H_{11}-	Acetone, 56 °C, 12 h	**C14**	71%	
89	II	**E4** (R_1 = $C_{11}H_{23}$)	C_5H_{11}-	Acetone, 56 °C, 12 h	**C14**	75%	
90	II	**E4** (R_1 = C_9H_{19})	C_6H_{13}-	Acetone, 56 °C, 12 h	**C14**	75%	
91	II	**E4** (R_1 = $C_{11}H_{23}$)	C_6H_{13}-	Acetone, 56 °C, 12 h	**C14**	81%	
92	II	**E4** (R_1 = C_9H_{19})	C_8H_{17}-	Acetone, 56 °C, 12 h	**C14**	80%	
93	II	**E4** (R_1 = $C_{11}H_{23}$)	C_8H_{17}-	Acetone, 56 °C, 12 h	**C14**	84%	
94	I	Deanol (**2**, n = 1, Y = H)	$C_{11}H_{23}$-	Et_2O,$CHCl_3$, 35 °C, 2 h, 5% $NaHCO_3$	**E4**	NA	14
95	I	Dimethylaminoisopropanol (**2**, n = 1, Y = CH_3))	$C_{11}H_{23}$-	Et_2O,$CHCl_3$, 35 °C, 2 h, 5% $NaHCO_3$	**E4**	NA	
96	II	**E4**	CH_3-	5 °C, 24 h	**C13**	NA	
97	I	Deanol (**2**, n = 1, Y = H)	$C_{13}H_{27}$-	CH_2Cl_2, RT, 5 h	**E4**	NA	21
98	I	Deanol (**2**, n = 1, Y = H)	$C_{15}H_{31}$-	CH_2Cl_2, RT, 5 h	**E4**	NA	
99	II	**E4**	-C_2H_4-	iPrOH, 100 °C, 6 h	**C6**	94%	
100	I	Deanol (**2**, n = 1)	CH_3-	Reflux, 12 h (overnight)	**E4**	NA	24
101	I	Deanol (**2**, n = 1)	C_2H_5-	Reflux, 12 h (overnight)	**E4**	NA	
102	I	Deanol (**2**, n = 1)	C_3H_7-	Reflux, 12 h (overnight)	**E4**	NA	
103	II	**E4**	Iodomethane	Et_2O, 25 °C, 72 h	**C12**	92-93%	

104	I	Deanol (**2**, n = 1)	Bn-CO-Gly /Imidazole	THF, 25 °C, 12 h	**E4**	75%	[25]
105	I	Deanol (**2**, n = 1)	Bn-CO-Gly /PO_2Cl_2	THF, Et_3N, 25 °C, 4h	**E4**	52%	
106	I	Deanol (**2**, n = 1)	Bn-CO-Gly / (4-NO_2)Ph	$CHCl_3$, 25 °C, 50 h	**E4**	95%	
107	II	**E4**	Iodomethane	EtOH, 25 °C, 24 h	**C12**	82%	
108	I	Deanol (**2**, n = 1)	Triethyl orthoformate	Citric acid, 100 °C, reduced pressure 920 mbar), 20 h	**E8**	40-46%	[26]
109	II	**E8**	Chloromethane	iPrOH, $NaHCO_3$, N_2, 85 °C, 5 h	**C16**	100%	
110	I	Dimethylaminopropanol (**2**, n = 2)	$C_9H_{19}O$-	Et_3N, 80 °C, 8 h	**E4**	85%	[27]
111	I	Dimethylaminopropanol (**2**, n = 2)	$C_{11}H_{23}O$-	Et_3N, 80 °C, 8 h	**E4**	91%	
112	I	Dimethylaminopropanol (**2**, n = 2)	$C_{13}H_{27}O$-	Et_3N, 80 °C, 8 h	**E4**	87%	
113	I	Dimethylaminopropanol (**2**, n = 2)	$C_{15}H_{31}O$-	Et_3N, 80 °C, 8 h	**E4**	84%	
114	I	Dimethylaminoisopropanol (**2**, n = *iso*-2)	$C_{11}H_{23}O$-	Et_3N, 80 °C, 8 h	**E4**	55%	
115	II	**E4** (n = 2, $R_1 = C_9H_{19}$)	CH_3I	$CHCl_3$, RT, 30 min	**C12**	86%	
116	II	**E4** (n = 2, $R_1 = C_{11}H_{23}$)	CH_3I	$CHCl_3$, RT, 30 min	**C12**	85%	
117	II	**E4** (n = 2, $R_1 = C_{13}H_{27}$)	CH_3I	$CHCl_3$, RT, 30 min	**C12**	85%	
118	II	**E4** (n = 2, $R_1 = C_{15}H_{31}$)	CH_3I	$CHCl_3$, RT, 30 min	**C12**	77%	
119	II	**E4** (n = *iso*-2, $R_1 = C_{11}H_{23}$)	CH_3I	$CHCl_3$, RT, 30 min	**C12**	65%	
120	I	Deanol (**2**, n = 1)	Bz-Gly / Benzotriazole	Acetonitrile, Et_3N, 1-(o-nitrophenylsulfonyloxy)benzotriazole, 25 °C, 24 h	**E4**	49%	[28]
121	I	Deanol (**2**, n = 1)	Bz-Ala / Benzotriazole	Acetonitrile, Et_3N, 1-(o-nitrophenylsulfonyloxy)benzotriazole, 25 °C, 24 h	**E4**	65%	
122	I	Deanol (**2**, n = 1)	Bz-Nle / Benzotriazole	Acetonitrile, Et_3N, 1-(o-nitrophenylsulfonyloxy)benzotriazole, 25 °C, 24 h	**E4**	73%	
123	I	Deanol (**2**, n = 1)	Bz-Phe / Benzotriazole	Acetonitrile, Et_3N, 1-(o-nitrophenylsulfonyloxy)benzotriazole, 25 °C, 24 h	**E4**	90%	
124	I	Deanol (**2**, n = 1)	Pht-Gly / Benzotriazole	Acetonitrile, Et_3N, 1-(o-nitrophenylsulfonyloxy)benzotriazol	**E4**	58%	

				e, 25 °C, 24 h			
125	I	Deanol (**2**, n = 1)	Pht-Val / Benzotriazole	Acetonitrile, Et_3N, 1-(o-nitrophenylsulfonyloxy)benzotriazole, 25 °C, 24 h	**E4**	80%	
126	I	Deanol (**2**, n = 1)	(*4*-OCH_3)Bz-Phe / Benzotriazole	Acetonitrile, Et_3N, 1-(o-nitrophenylsulfonyloxy)benzotriazole, 25 °C, 24 h	**E4**	73%	
127	I	Dimethylaminopropanol (**2**, n = 2)	(*4*-OCH_3)Bz-Phe / Benzotriazole	Acetonitrile, Et_3N, 1-(o-nitrophenylsulfonyloxy)benzotriazole, 25 °C, 24 h	**E4**	65%	
128	II	**E4**	CH_3I	Acetone, 25 °C, 24 h	**C12**	63-89%	
129	I	Deanol (**2**, n = 1)	$C_8H_{17}O$-	Et_3N, 80 °C, 24 h	**E4**	NA	[29]
130	I	Deanol (**2**, n = 1)	$C_{10}H_{21}O$-	Et_3N, 80 °C, 24 h	**E4**	NA	
131	I	Deanol (**2**, n = 1)	$C_{12}H_{25}O$-	Et_3N, 80 °C, 24 h	**E4**	NA	
132	I	Deanol (**2**, n = 1)	$C_{14}H_{29}O$-	Et_3N, 80 °C, 24 h	**E4**	NA	
133	I	Deanol (**2**, n = 1)	$C_{16}H_{33}O$-	Et_3N, 80 °C, 24 h	**E4**	NA	
134	II	**E4** ($R_1 = C_8H_{17}O$)	1,4-dichloro-2-butyne	iPrOH, 85 °C, 3 h	**C15**	50%	
135	II	**E4** ($R_1 = C_{10}H_{21}O$)	1,4-dichloro-2-butyne	iPrOH, 85 °C, 3 h	**C15**	46%	
136	II	**E4** ($R_1 = C_{12}H_{25}O$)	1,4-dichloro-2-butyne	iPrOH, 85 °C, 3 h	**C15**	53%	
137	II	**E4** ($R_1 = C_{14}H_{29}O$)	1,4-dichloro-2-butyne	iPrOH, 85 °C, 3 h	**C15**	51%	
138	II	**E4** ($R_1 = C_{16}H_{33}O$)	1,4-dichloro-2-butyne	iPrOH, 85 °C, 3 h	**C15**	45%	
139	I	Chloroethyldimethylamine (**6**)	$C_2H_5OOCCH=C(CH_3)NHCH_2$-	Ethyl acetate, 77 °C, 6h	**E4**	61%	[25]
140	I	Chloroethyldimethylamine (**6**)	$C_2H_5OOCCH=C(CH_3)NH(CH_2)_2$-	Ethyl acetate, 77 °C, 6h	**E4**	67%	
141	I	Chloroethyldimethylamine (**6**)	$C_2H_5OOCCH=C(CH_3)NH(CH_2)_3$-	Ethyl acetate, 77 °C, 6h	**E4**	70%	
142	II	**E4** ($R_1 = C_2H_5OOCCH=C(CH_3)NH(CH_2)$)	Iodomethane	Et_2O, 25 °C, 24 h	**C12**	90%	
143	II	**E4** ($R_1 = C_2H_5OOCCH=C(CH_3)NH(CH_2)_2$)	Iodomethane	Et_2O, 25 °C, 24 h	**C12**	82%	
144	II	**E4** ($R_1 = C_2H_5OOCCH=C(CH_3)NH(CH_2)_3$)	Iodomethane	Et_2O, 25 °C, 24 h	**C12**	77%	

145	I	Carnitine (**7**)	C_3H_7- / C_2H_3-	Acetonitrile, Candida actarctica lipase B, 40 °C, 6 days	**C17**	66%	22
146	I	Choline chloride (**5**)	C_3H_7- / C_2H_3-	2-methyl-2-butanol, Candida actarctica lipase B, 40 °C, 12 h	**C7**	91%	
147	I	Choline chloride (**7**)	Meldrum's acid	Acetonitrile, 82 °C, 5 h	**C18**	100%	23
148	I	2-Iodoethanol (**8**, n = 1)	Diphenyl carbonate	Acetone, K_2CO_3, 25 °C, 48 h	**E9**	79%	30
149	I	3-Iodopropanol (**8**, n = 2)	Diphenyl carbonate	THF, K_2CO_3, 25 °C, 48 h	**E9**	71%	
150	II	**E9** (n = 1)	$C_{10}H_{21}$-	Ethyl acetate, 80 °C, 72 h	**C19**	80%	
151	II	**E9** (n = 1)	$C_{12}H_{25}$-	Ethyl acetate, 80 °C, 72 h	**C19**	87%	
152	II	**E9** (n = 1)	$C_{14}H_{29}$-	Ethyl acetate, 80 °C, 72 h	**C19**	82%	
153	II	**E9** (n = 2)	$C_{10}H_{21}$-	Ethyl acetate, 80 °C, 72 h	**C19**	82%	
154	II	**E9** (n = 2)	$C_{12}H_{25}$-	Ethyl acetate, 80 °C, 72 h	**C19**	89%	
155	II	**E9** (n = 2)	$C_{14}H_{29}$-	Ethyl acetate, 80 °C, 72 h	**C19**	88%	
156	I	Dimethylaminopropanol (**2**, n = 2, Y = H)	Diphenyl carbonate, C_8H_{17}-	Et_3N	**E10**	91%	31
157	I	Dimethylaminopropanol (**2**, n = 2, Y = H)	Diphenyl carbonate, $C_{10}H_{21}$-	Et_3N	**E10**	85%	
158	I	Dimethylaminopropanol (**2**, n = 2, Y = H)	Diphenyl carbonate, $C_{12}H_{25}$-	Et_3N	**E10**	91%	
159	I	Dimethylaminoisopropanol (**2**, n = 1, Y = CH_3)	Diphenyl carbonate, $C_{12}H_{25}$-	Et_3N	**E10**	75%	
160	II	**E10** (n = 2, Y = H, $R_1 = C_8H_{17}$)	-CH_2-	Acetonitrile, 80 °C, 24 h	**C20**	86%	
161	II	**E10** (n = 2, Y = H, $R_1 = C_{10}H_{21}$)	-CH_2-	Acetonitrile, 80 °C, 24 h	**C20**	79%	
162	II	**E10** (n = 2, Y = H, $R_1 = C_{12}H_{25}$)	-CH_2-	Acetonitrile, 80 °C, 24 h	**C20**	85%	
163	II	**E10** (n = 1, Y = CH_3, $R_1 = C_{12}H_{25}$)	-CH_2-	Acetonitrile, 80 °C, 24 h	**C20**	68%	
164	II	**E10** (n = 2, Y = H, $R_1 = C_{12}H_{25}$)	Iodomethane	Acetonitrile, RT, 30 min	**C21**	85%	
165	II	**E10** (n = 1, Y = CH_3, $R_1 = C_{12}H_{25}$)	Iodomethane	Acetonitrile, RT, 30 min	**C21**	87%	
166	II	**E10** (n = 2, Y = H, $R_1 = C_8H_{17}$)	**E9** (m = 1)	Acetonitrile, 80 °C, 72 h	**C22**	74%	
167	II	**E10** (n = 2, Y = H, $R_1 = C_8H_{17}$)	**E9** (m = 2)	Acetonitrile, 80 °C, 72 h	**C22**	68%	
168	I	Deanol (**2**, n = 1)	(2-CH_3-4-Cl)Ph-O-CH_3-	$CHCl_3$, 4 °C (ice bath), 10 min	**E4**·HCl	98%	19
169	II	**E4**·HCl	Et_3N	$CHCl_3$, 25 °C	**E4**	82%	

170	III	**E4**	C_9H_{19}-	Acetone, 55 °C, 24 h	**C10**	73%	
171	I	Deanol (**2**, n = 1)	1-(3,6-Cl-2-OCH_3)Ph-	Et_3N, $CHCl_3$, 4 °C (ice bath), 20 min	**E4**	78%	20
172	II	**E4**	$C_{10}H_{21}$-	Acetone, 55 °C, 24 h	**C11**	85%	
173	II	**E4**	$C_{12}H_{25}$-		**C11**	78%	
174	II	**E4**	$C_{14}H_{29}$-		**C11**	75%	
175	I	2-bromoethanol (**8**, n = 1)	Lidocaine	100 °C, 8 h	**Q1**	81%	33
176	I	3-bromopropanol (**8**, n = 2)	Lidocaine	100 °C, 8 h	**Q1**	80%	
177	I	Deanol (**2**, n = 1)	2-bromo-N-cyclohexylacetamide	110 °C, 8 h	**Q2**	NA	
178	II	**Q1** (n = 1)	Triphosgene, C_5H_{11}-	CH_2Cl_2, Pyridine, 50 °C, 16 h	**C26**	39%	
179	II	**Q1** (n = 1)	Triphosgene, C_7H_{15}-	CH_2Cl_2, Pyridine, 50 °C, 16 h	**C26**	43%	
180	II	**Q1** (n = 1)	Triphosgene, C_9H_{19}-	CH_2Cl_2, Pyridine, 50 °C, 16 h	**C26**	41%	
181	II	**Q1** (n = 1)	Triphosgene, $C_{12}H_{25}$-	CH_2Cl_2, Pyridine, 50 °C, 16 h	**C26**	45%	
182	II	**Q1** (n = 2)	Triphosgene, C_7H_{15}-	CH_2Cl_2, Pyridine, 50 °C, 16 h	**C26**	21%	
183	II	**Q1** (n = 1)	C_8H_{17}-	CH_2Cl_2, Pyridine, 40 °C, 12 h	**C27**	37%	
184	II	**Q2**	Triphosgene, C_7H_{15}-	CH_2Cl_2, Pyridine, 50 °C, 24 h	**C28**	38%	
185	II	**C18**	**9** (X = OCH_3, Y = OCH_3)	Ethanol, proline, reflux, 12 h	**C30**	48%	23
186	II	**C18**	**9** (X = H, Y = H)	Ethanol, proline, reflux, 12 h	**C30**	34%	
187	II	**C18**	**9** (X = OH, Y = H)	Ethanol, proline, reflux, 12 h	**C30**	61%	
188	II	**C18**	**9** (X = OCH_3, Y = H)	Ethanol, proline, reflux, 12 h	**C30**	50%	

3. Synthesis of betaine-type esterquats

3.1. Synthesis from haloacyl halides

One of the most common methods enabling the synthesis of betaine esters is based on the acylation of an appropriate alcohol with the respective haloacyl halide (chlorine- or bromine-based ones), followed by the alkylating of tertiary amines.[13,34–42]

Thompson et al. in 1989 prepared the esters of betaine by dropwise addition of dichloromethanoic solution of chloroacetyl chloride **10** (n = 1) into dichloromethanoic solution of dodecanol (**Figure S17** and **Table S2**, No. 1-3). Then, the excess acyl chloride was simply washed off with 5% sodium carbonate and water. The organic phase was dried over magnesium sulfate. The product **E11** ($R_1 = C_{10}H_{21}$) was obtained by filtration of the drying agent followed by evaporation of the applied solvent. Afterward, the product was dissolved in diethyl ether and introduced into a solution of dimethylamine in the same solvent. The precipitate was then filtered off, and the residual solvent was evaporated. The product **E12** was purified by dissolution in diethyl ether and washing with sodium hydroxide solution, followed by drying over anhydrous magnesium sulfate. The product **E12** was then precipitated with hydrogen chloride, washed with diethyl ether and dried. Then, the crude product was purified by dissolution in hydrochloric acid and subsequent extraction with diethyl ether in the presence of sodium hydroxide to obtain **E12** in the form of hydrochloride. The extracts were dried and mixed with 4-nitrobenzyl bromide. Then, the mixture was refluxed, and the solvent was evaporated. Finally, product **B1** was purified by recrystallization from a mixture of ethyl acetate and benzene (1:1 v/v).[41]

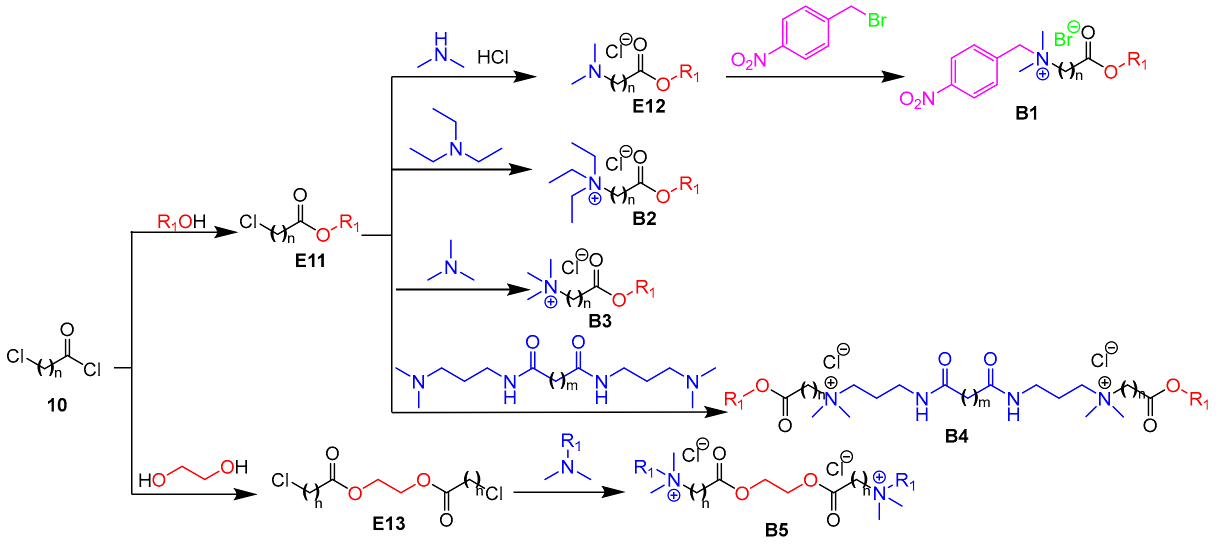

Figure S17. Synthesis of betaine esters from chloroacyl chlorides (more details are available in **Table S2**, No. 1-28).

Lindstedt et al. in 1990 proposed the use of chloroacetyl chloride **10** (n = 1) for acylating fatty alcohols (decanol, dodecanol, tetradecanol, hexadecanol and octadecanol) in dry dichloromethane (**Figure S17** and **Table S2**, No. 4-9). The acylation was carried out by slow dropwise addition of an acylating agent. After isolation of the acidic impurities, the solvent was removed. The next step involved dissolution of product **E11** (R_1 = $C_{10}H_{21}$, $C_{12}H_{25}$, $C_{14}H_{29}$, $C_{16}H_{33}$, $C_{18}H_{37}$) in acetone and subsequent dropwise addition of an acetonic solution of triethylamine, which initiated its quaternization, resulting in the formation of esterquat **B2**.[34] Nonetheless, it should be noted that this procedure is considered an expansion of the method proposed by Holden in 1984, referring to the synthesis of acyl azides *via* alkylation.[35]

In 2004, Nederberg et al. described the usage of a chloroacetyl chloride homologue, namely, 4-chlorobutyric acid **10** (n = 3). As shown in **Figure S17** (and **Table S2**, No. 10-13), **10** was added to nitrogen-purged chloroformic solutions of pyridine and two benzyl esters: benzyl-polycaprolactone (polymeric) and benzyl 6-hydroxyhexanoate (nonpolymeric). After the reaction, the polymeric product **E11** (R_1 = $C_6H_5O[C(=O)C_5H_{10}O]_n$-H, $C_6H_5OC(=O)C_5H_{10}OH$) was purified by precipitation in methanol and then filtered off and dried, whereas flash chromatography with a mixture of hexane and ethyl acetate (1:1 v/v) was performed for purification of the nonpolymeric **E11**. Both products **E11** were then dissolved in dry acetonitrile and purged with nitrogen before the addition of condensed trimethylamine. Due to increased pressure, the reactions were performed in sealed tubes. Finally, product **B3** was precipitated from methanol and dried.[36]

Another unique approach leading to the synthesis of gemini esterquats is based on alkylation of the appropriate diamine or combination of two amines with the appropriate dihalide. In 2019, Wu et al. synthesized appropriate diamines by dehydration of amines and dicarboxylic acid with phosphorus acid in xylene (**Figure S17** and **Table S2**, No. 14-25). The obtained diamines were later alkylated in ethyl acetate by **E11** (m = 2, 3, 4), n=1, R_1= C_8H_{17}, $C_{10}H_{21}$, $C_{12}H_{25}$) to form gemini esterquats **B4**.[42]

In 2013, Fatma et al. utilized choloracetyl chloride **10** (n = 1) for the acylation of ethylene glycol (**Figure S17** and **Table S2**, No. 26-28) according to the procedure described by Gao et al. in 2008. First, both reagents were mixed without a solvent in a nitrogen atmosphere. The postreaction mixture was washed with brine, and then the obtained product **E13** was dissolved in diethyl ether and dried over magnesium sulfate. After evaporation of the solvent, the product was mixed with dodecyl- or hexadecyldimethylamine dissolved in ethyl acetate. Next, ethyl acetate was evaporated, and the crude product **B5** (R_1 = $C_{12}H_{25}$, $C_{16}H_{33}$) was purified by recrystallization from a mixture of ethyl acetate and ethanol (5:1 v/v).[38]

An analogous method to that previously reported for choline-type esterquats (Thompson and Lindstedt) was proposed in 2007 by Tehrani-Bagha et al. As shown in **Figure S18** (and **Table S2**, No. 29-31), the authors used a bromine analog instead of chloroacetyl chloride – bromoacetyl bromide **11** (n = 1) to obtain ester **E14** ($R_1 = C_{12}H_{25}$). The acylating agent was added dropwise into the solution of dodecanol, both in dichloromethane, and then the excess of **11** was washed off with sodium hydrogen carbonate solution. Trimethylamine was next introduced in the form of bubbles. The precipitated sediment was filtered off and washed with ethyl acetate to obtain product **B6**. Interestingly, Tehrani-Bagha et al. also used tetramethyethylenediamine instead of trimethylamine to obtain the gemini esterquat **B7**; however, the quaternization was carried out in acetone, and after filtration of precipitate **B7**, it was purified by washing with ethanol.[13]

Zhou et al. in 2014 performed another synthesis leading to interesting polymeric esterquats. First, a solution of dodecanol and diisopropylethylamine in dichloromethane was added dropwise to bromoacetyl bromide **11** (n = 1) dissolved in the same solvent (**Figure S18** and **Table S2**, No. 32-33). Then, the precipitate was filtered off and washed with hydrochloric acid, sodium hydroxide and brine to remove residual substrates. The organic phase was further dried over magnesium sulfate, and the solvent was evaporated. The obtained product **E14** ($R_1 = C_{12}H_{25}$) was then dried in vacuum and added dropwise in the form of a solution of dimethylformamide (DMF) into a solution of permethylated hyperbranched polyethyleneimine. After quaternization, DMF was evaporated, and residual bromoester was removed by a syringe. The obtained crude product **B8** (m = *poly*) was dissolved in chloroform, precipitated with the use of diethyl ether and dried in a vacuum.[39]

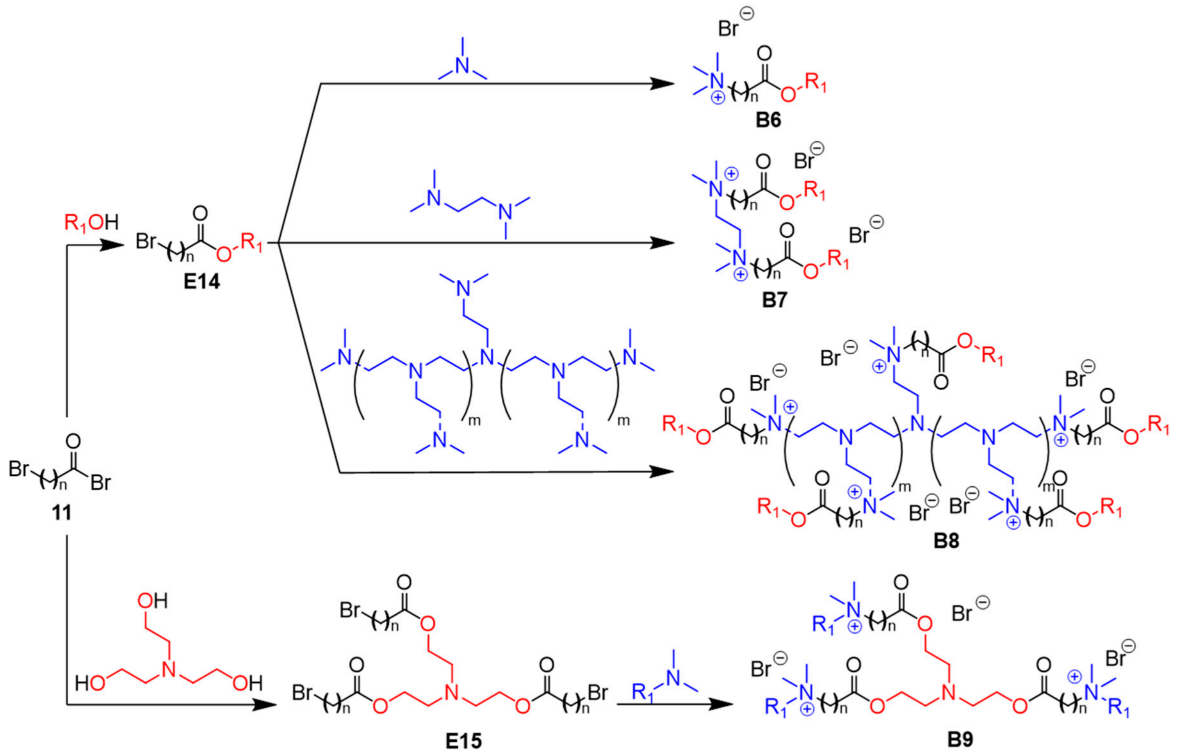

Figure S18. Synthesis of betaine esters from bromoacyl bromides (more details are available in **Table S2**, No. 29-38).

In 2014, Liu et al. proposed a method based on the dropwise addition of bromoacetyl bromide **11** (n = 1) and potassium carbonate solutions (in dichloromethane and water, respectively) into a dichloromethanoic solution of triethanolamine (**Figure S18** and **Table S2**, No. 34-38). The organic layer was subsequently washed with water and dried over magnesium sulfate, and then the solvent was evaporated. The obtained product **E15** was then purified chromatographically with petroleum ether and ethyl acetate (2:1 v/v) as the eluent. **E15** was next dissolved in acetone, and a respective amine (decyl-, dodecyl-, tetradecyl- and hexadecyldimethylamine) was added to initiate the quaternization. Then, the solvent was evaporated, and product **B9** (R_1 = $C_{10}H_{21}$, $C_{12}H_{25}$, $C_{14}H_{29}$, $C_{16}H_{33}$) was precipitated from dry diethyl ether. Finally, **B9** was isolated, washed with ether and dried.[40]

3.2. Synthesis from haloesters

Similar procedures utilizing haloesters instead of haloacetyl halides were also carried out to obtain various betaine-type esterquats.[43–50] This convenient solution allows immediate quaternization after the addition of an appropriate tertiary amine. However, the use of primary or secondary amines results in the formation of aminoesters, which have to be subsequently quaternized with the respective alkyl halide.

In 2008, Zhang et al. described the synthesis of betaine derivatives from tertiary amine and terminal bromoester. As presented in **Figure S19** (and **Table S2**, No. 39-41), the bromoesters used in the study were as follows: methyl bromoacetate **12** (n = 1, m = 0), ethyl bromobutyrate **12** (n = 3, m = 1) and ethyl bromocaproate **12** (n = 5, m = 1). The reactions of bromoesters with N-(3-dimethylaminopropyl)acrylamide were carried out in acetonitrile under an inert gas (nitrogen or argon) atmosphere. The obtained solid product **B10** ($R_1 = C_3H_6NHC(=O)CH=CH_2$) required washing with small portions of acetone to remove the remaining substrates.[43] Interestingly, a slightly modified procedure was described in 2013 by Messadi et al. The authors utilized a solution of an appropriate tertiary amine (triethyl-, tripropyl- and tributylamine, as well as N-methylpyrrolidine) in ethyl acetate (**Figure S19** and **Table S2**, No. 42-45). First, all the solutions were cooled to 4 °C, and ethyl bromoacetate **12** (n = 1, m = 1) was added dropwise under constant stirring. The obtained crude products **B11** ($R_1 = C_2H_5$, C_3H_7, C_4H_9) and **B12** ($R_1 = C_4H_8$) precipitated from the applied solvent, which after isolation were washed with a portion of ethyl acetate. The proposed method of product purification involved recrystallization from a mixture of ethanol and ethyl acetate (1:9 v/v).[44]

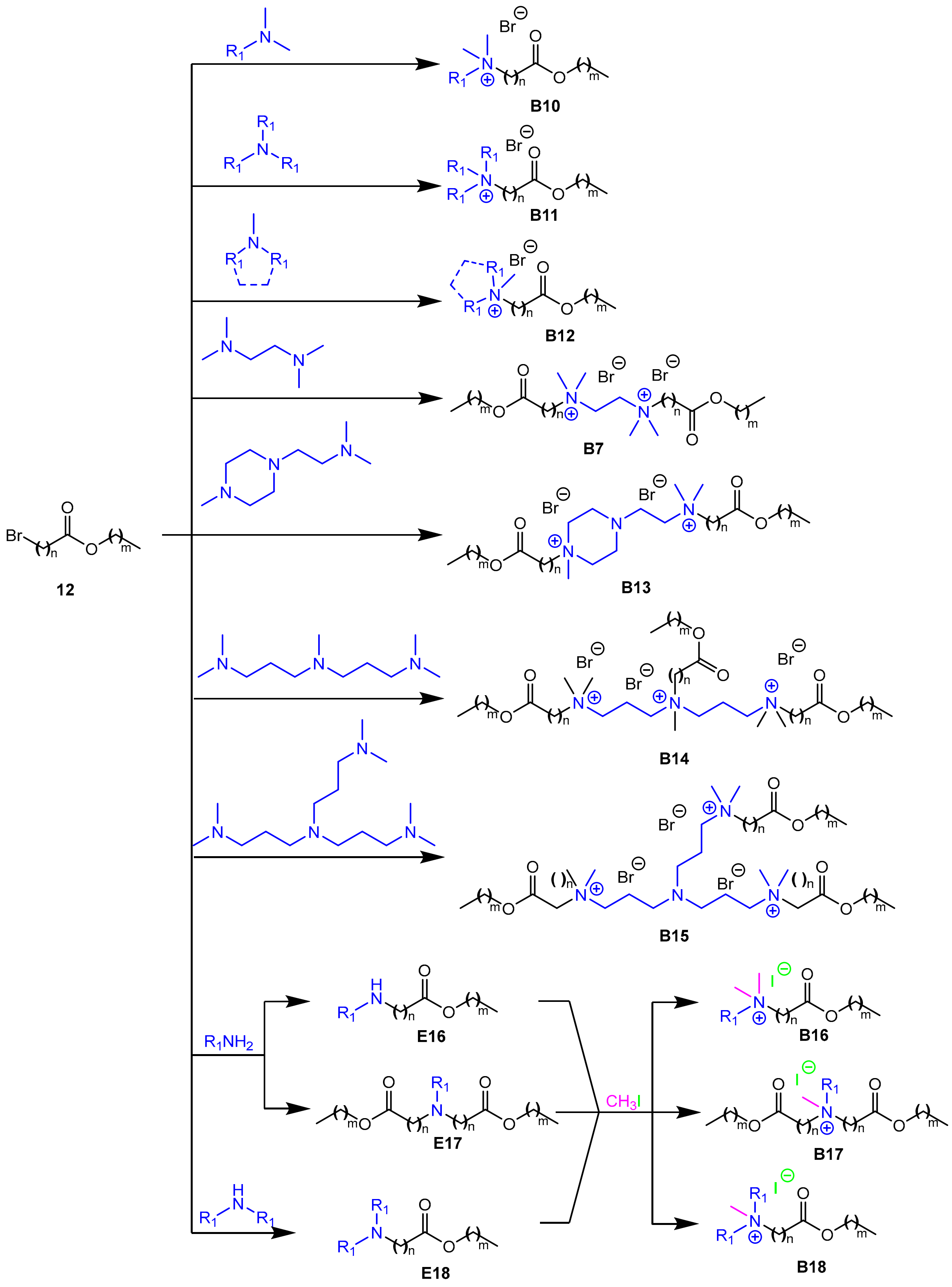

Figure S19. Synthesis of betaine esters from ω-bromoesters (more details are available in **Table S2**, No. 39-95).

In 2015, Kim et al. described a procedure analogous to the method given by Zhang et al. The synthesis was based on dropwise addition of methyl- or ethyl bromoacetate **12** (n = 1, m = 0, 1) into acetonitrile solutions of respective cyclic tertiary amines, such as methyl derivatives of pyrrolidine, piperidine, morpholine and imidazole, under an inert gas (nitrogen) atmosphere as well (**Figure S19** and **Table S2**, No. 46-49). The products **B12** (R_1 = *cyclo*-$CH_2N(CH_3)C_2H_4$, *cyclo*-$C_2H_4OC_2H_4$, *cyclo*-C_5H_{10}, *cyclo*-C_4H_8) appeared to be insoluble in acetonitrile. Therefore, they were filtered off, washed with acetone and dried.[45]

Allen et al. in 2017 proposed a procedure for obtaining various esterquats comprising multiple cationic moieties (**Figure S19** and **Table S2**, No. 50-84). The procedure was based on mixing *N*,*N*-dimethylbenzylamine, tetramethylenediamine, 1-(2-dimethylaminoethyl)-4-methylpiperazine, 2,6,10-trimethyl-2,6,10-triazaundecane or tris(*N*,*N*-dimethylaminopropyl)amine with appropriate bromoacetates **12** (hexyl, heptyl, octyl, nonyl, decyl, dodecyl, tetradecyl) in acetonitrile to obtain products **B10**, **B7**, **B13**, **B14** and **B15** (m = 5, 6, 7, 8, 9, 11, 13, n = 1), respectively. After the reaction, the products **B10**, **B14** and **B15** were concentrated in vacuum and then purified by washing with hexane (for products **B10** and **B15)** or with a mixture of hexane and acetone (2:1 v/v) (for **B14**). In the case of **B7** and **B13**, hexane was added after the reaction, and the precipitate was filtered off and rinsed again with hexane.[46]

Yasa et al. in 2016 and 2017 applied a unique procedure, which was based on alkylation of long-chain or cyclo-side chain amine with the obtained earlier methyl ester of 11-bromoundecane acid **12** (n = 10, m = 0). Alkylation was carried out in DMF in the presence of anhydrous potassium carbonate as a catalyst (**Figure S19** and **Table S2**, No. 85-95). The inorganic salt (KBr) that precipitated from the postreaction mixture was filtered off. Then, hydrophobic products **E16**, **E17** (R_1 = C_6H_{13}, $C_{12}H_{25}$, $C_{18}H_{37}$) and **E18** (R_1 = *cyclo*-C_6H_{11}, C_8H_{17}) were extracted with ethyl acetate and washed with water to remove residual DMF. The obtained extracts were dried over anhydrous sodium sulfate, and then the solvent was removed. The products **E16**, **E17** and **E18** were isolated using chromatography. A mixture of chloroform and methanol 98:2 v/v was used as the eluent for **E16** and **E18**, whereas a ratio of 99:1 was applied for **E17**. The subsequent quaternization was carried out with iodomethane in chloroformic conditions with the addition of a catalytic amount of potassium carbonate. After the reaction, crude products **B16**, **B17** and **B18** were purified by evaporation of residual solvent and iodomethane, followed by chromatographic separation using 2.5-3% methanol in chloroform.[47,51]

Bhadani et al. in 2014 described a successful quaternization of dodecyldimethylamine with previously prepared chloroformic solutions of bromoacetates of mono-, di- and triethylene glycol **13** (m = 1, 2 or 3) (**Figure S20** and **Table S2**, No. 96-98). After evaporation of the solvent from the postreaction mixture, crude product **B19** (R_1 = $C_{12}H_{25}$) was washed with hexane and precipitated from acetone or ethyl acetate. Isolated sediments were finally dried to obtain gemini esterquats **B19**.[48]

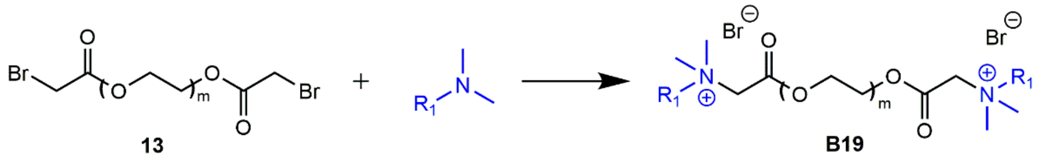

Figure S20. Synthesis of betaine esters from ethylene glycol bromoacetates (more details are available in **Table S2**, No. 96-104).

In 2020, Wen et al. applied a procedure given by Bhadani et al. to obtain structurally similar esters. After quaternization of ethylene glycol bromoacetate **13** (m = 1), dissolved in chloroform, with the respective amine (octyl-, decyl-, dodecyl-, tetradecyl-, hexadecyl- and octadecyldimethylamine), the obtained precipitates **B19** (R_1 = C_8H_{17}, $C_{10}H_{21}$, $C_{12}H_{25}$, $C_{14}H_{29}$, $C_{16}H_{33}$) (**Figure S20** and **Table S2**, No. 99-104) were filtered off, washed with hexane, and finally recrystallized from acetone.[49]

In 2019, Garcia et al. presented another method for the synthesis of oligomeric esterquats based on the initial esterification of chloroacetic acid with octanol, decanol or dodecanol (**Figure S21** and **Table S2**, No. 105-109), wherein a small amount of concentrated sulfuric acid was used as a catalyst. Then, the postreaction mixture was diluted with diethyl ether, and the residues of sulfuric acid were removed with a saturated solution of sodium carbonate. Next, the organic layer was washed with water and dried over anhydrous sodium sulfate. Evaporation of the solvent allowed the authors to obtain products **14** (m = 7, 9 or 11). The subsequent quaternization step was carried out by the addition of the obtained chloroesters **14** (m = 7, 9 or 11) into a solution of pentamethyldiethylenetriamine (PMDTA) in acetonitrile. The isolated products **B20** and **B21** were then purified by recrystallization from a mixture of acetonitrile and diethyl ether (1:1 v/v).[50]

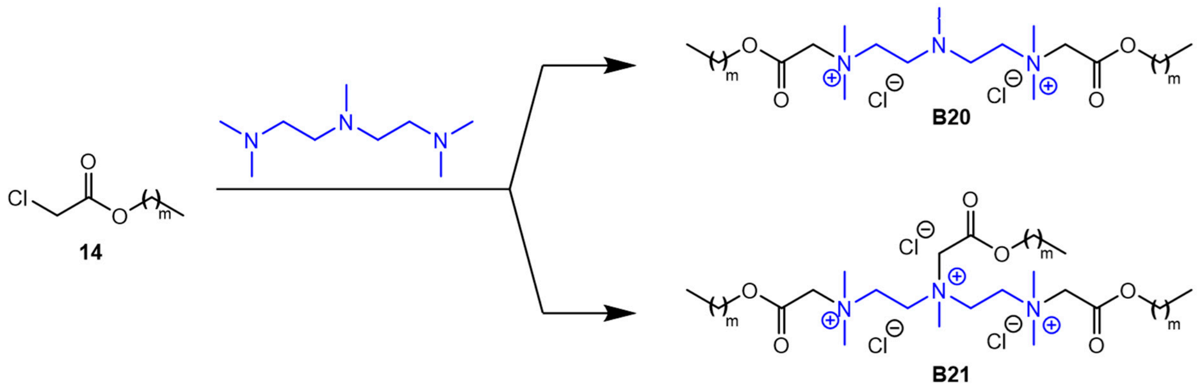

Figure S21. Synthesis of betaine esters from alkyl chloroacetate (more details are available in **Table S2**, No. 105-109).

3.3. Synthesis from chlorobetainyl chloride

A literature survey allowed us to gather a few procedures describing the use of betaine after its transformation into an acylating agent. Such a form of betaine was found to be capable of forming esters in reaction with alcohols.[52,53]

Granö et al. in 2000 proposed a method involving acylating with chlorobetainyl chloride **15** (**Figure S22** and **Table S2**, No. 110).[52] This method involved the dropwise addition of thionyl chloride into a dichloromethanoic solution of betaine, resulting not only in the substitution of carboxylate oxygen atoms but also in the occurrence of side reactions, wherein the released chloride anion became the counterion for betaine. The removal of the byproduct sulfur dioxide was achieved by bubbling the postreaction mixture in a washing solution containing potassium hydroxide. The obtained acylating agent **15** was then utilized in the reaction with starch. The acylation was carried out in dioxane with the addition of pyridine as a catalyst. Despite the fact that starch is not a typical alcohol, the product of this reaction – **B22** – can still be classified as betaine-type esterquat.[52] A slightly different procedure to obtain these esterquats was described by Sievänen et al. in 2015. The authors utilized betaine in the form of hydrochloride, which was quenched in excess thionyl chloride (according to a report of Vassel and Skelly from 1963, who described the synthesis of chlorobetainyl chloride).[53,54] The reaction was carried out under an argon atmosphere, and then chlorobetainyl chloride **15** was crystallized with the addition of toluene. The ablation step assumed dissolving the second reagent (cellulose) in dimethylacetamide with the addition of lithium chloride as a water absorber (**Figure S22** and **Table S2**, No. 111). Next, pyridine was added into the solution of cellulose, and then chlorobetainyl chloride was introduced to obtain esterquat **B23**. Utilization of pyridine allowed the authors to capture and easily isolate the side product of the acylation reaction: hydrogen chloride.[53]

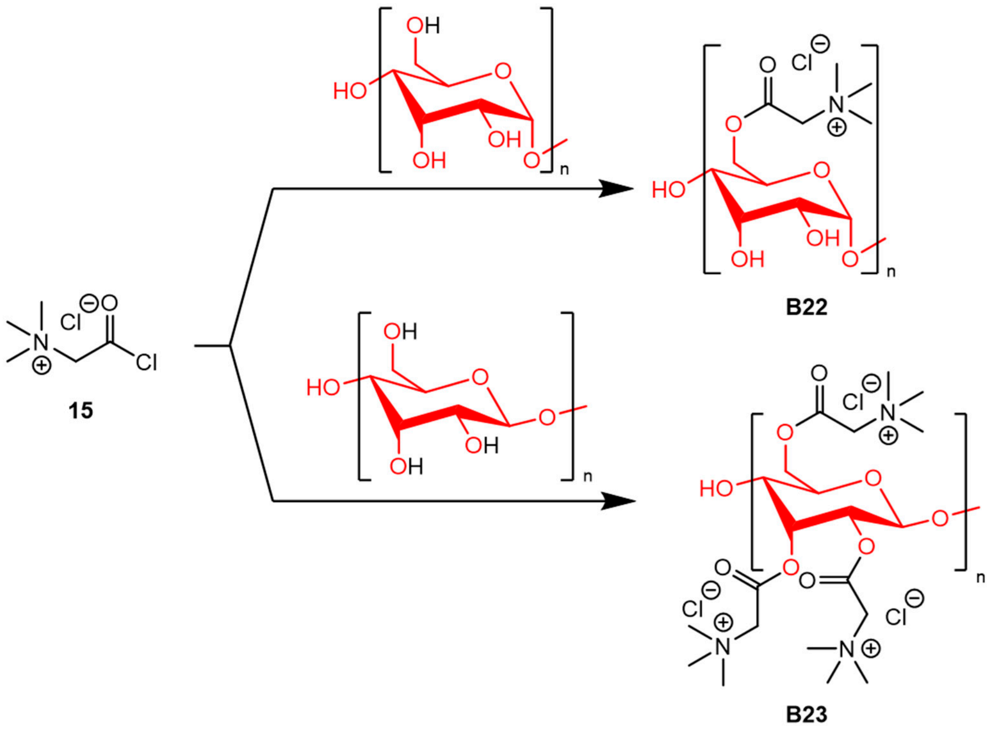

Figure S22. Synthesis of betaine esters by acylation of biopolymers with chlorobetainyl chloride (more details are available in **Table S2**, No. 110-111).

3.4. Synthesis of glycine betaine by classic esterification

Performing classic Fischer esterification is also possible in the case of glycine betaine. Nonetheless, its application in zwitterionic form often requires the addition of other chemicals to provide acidic conditions, which allows higher reaction yields to be achieved. Interestingly, due to the unique structure of glycine betaine and its ability to capture acids, utilizing its derivatives, such as glycine betaine hydrochloride, has been established to be a highly convenient and beneficial solution.[55–60]

In 2008, Goursaud et al. reported a reaction in which a suspension of zwitterionic betaine in methanesulfonic acid **16** ($R_2 = CH_3$) was mixed with dodecanol, octadecanol or octadec-9-enol (**Figure S23** and **Table S2**, No. 112-114). Then, water was removed under reduced pressure, and the crude products **B24** ($R_1 = C_{12}H_{25}$, $C_{18}H_{37}$, $C_{18}H_{35}$) were purified *via* various methods to determine their individual properties.[55]

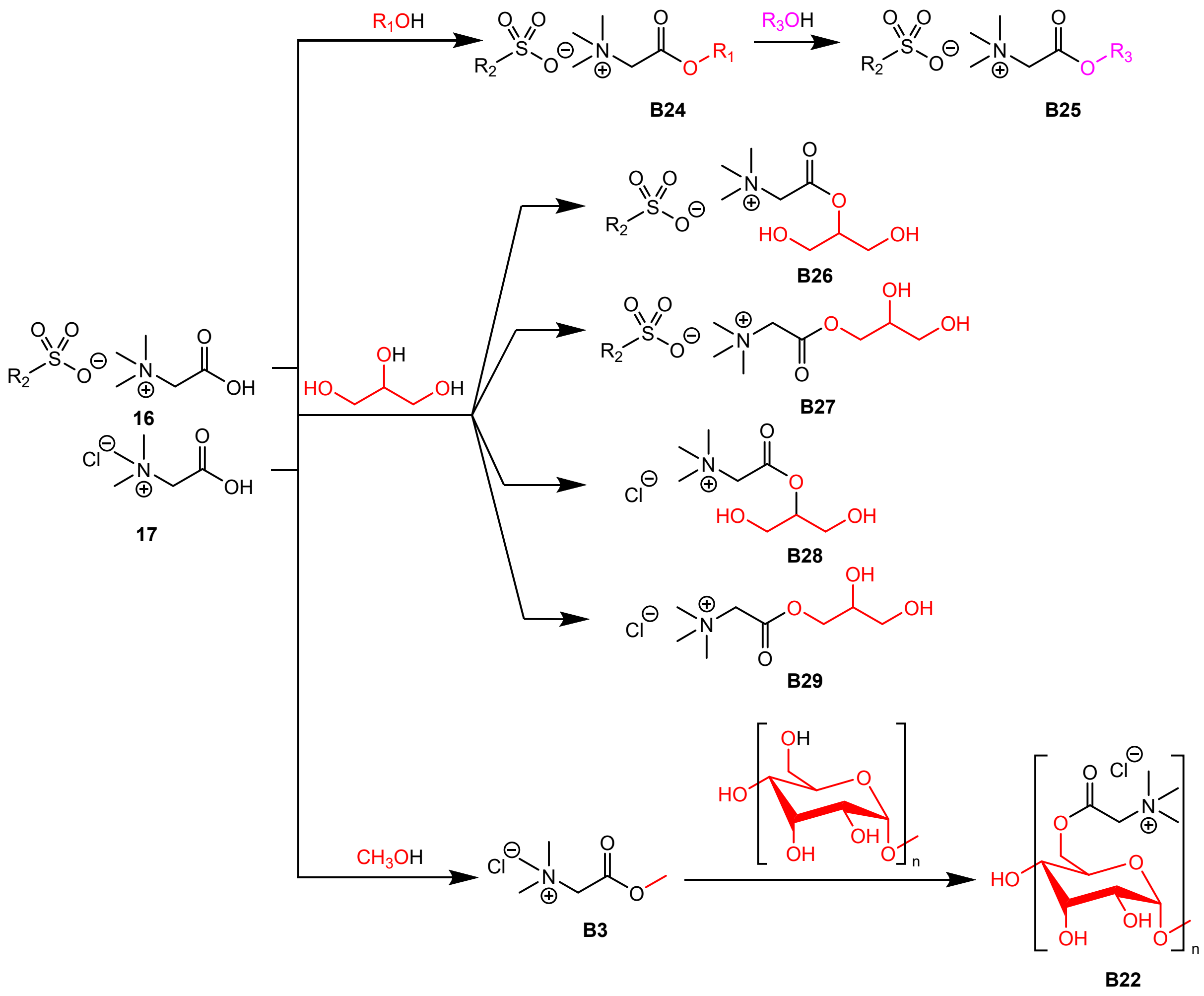

Figure S23. Direct esterification of glycine betaine in a zwitterionic or protonated form (more details are available in **Table S2**, No. 112-139).

In 2015, De Gaetano et al. proposed a method based on a dropwise addition of excess mixture of methanesulfonic acid and respective alcohol (hexanol, dodecanol, tetradecanol, hexadecanol or docosanol, (**Figure S23** and **Table S2**, No. 115-119) into a zwitterionic glycine betaine. The excess acid acted either as a catalyst or counterion for the formed product **B24** (R_1 = C_6H_{13}, $C_{12}H_{25}$, $C_{14}H_{29}$, $C_{16}H_{33}$, $C_{20}H_{41}$, R_2 = CH_3). According to the protocols, the esterification reaction was carried out in the Dean-Stark apparatus, which in combination with high temperature enabled the effective removal of water. De Gaetano et al. also showed an alternative approach using the dropwise addition of alcohol into a suspension of betaine (or its methanesulfonate) **16** (R_2 = CH_3) and methanesulfonic acid in an appropriately designed reaction system. Applying high temperature along with reduced pressure during the process was essential for effective evaporation of the formed water molecules.[56]

A similar procedure, however, demonstrating the use of catalytic amounts of methanesulfonic acid instead of its excess, was proposed by Pérusse et al. in 2015. Methanesulfonic acid was

utilized as a catalyst in the esterification of betaine **16** (R_2 = CH_3) with a short-chain alcohol (butanol), which was added in excess (**Figure S23** and **Table S2**, No. 120-122). The reduced pressure was found to be beneficial for the removal of both water and excess solvent. It should be stressed that the synthesis of product **B24** (R_1 = C_4H_9) was not the final step. According to the authors, the presence of a short alkyl chain elevated the reactivity of such betaine esterquats toward transesterification with longer chain alcohols, such as tetradecanol. Due to the necessity of removing butanol, the reaction was carried out under reduced pressure. The use of a basic catalyst (sodium hydrogen carbonate) allowed the authors to obtain the product **B25** (R_3 = $C_{14}H_{29}$) in high yield. The authors also investigated the effect of the presence of an acidic catalyst (methanesulfonic acid); however, it has not proven to be beneficial in this particular application.[57]

An analogous procedure was described by Journoux-Lapp et al. in 2017, although there was a difference in the utilization of various betaine salts **16** (R_2 = CH_3) and **17** instead of acidic catalysts as well as glycerin as a reacting alcohol. As demonstrated in **Figure S23** (and **Table S2**, No. 123-132), this procedure led to the formation of products **B26**, **B27**, **B28** and **B29** (R_2 = CH_3, OH, CF_3, *4*-$CH_3C_6H_4$). The mentioned esterquats contained various anions, such as methanesulfonate, sulfate, trifluoromethanesulfonate, *p*-toluenesulfonate and chloride.[58]

Sharma et al. in 2021 proposed another approach to the synthesis of starch betainates through a methyl ester of betaine, which was obtained according to the procedure described by Webb in 1969 (**Figure S23** and **Table S2**, No. 133-139).[61] First, betaine hydrochloride **17** was dissolved in methanol, and thionyl chloride was added dropwise. Then, the solvent was evaporated, and the crude product **B3** was washed with diethyl ether and dried under vacuum. The next step was based on transesterification with the use of starch, which was performed under basic as well as acidic conditions. In the case of basic conditions, starch was activated previously by refluxing with sodium hydroxide in ethanol, while for acidic conditions, sulfuric acid was added as a catalyst. Both types of reactions were performed in DMF and DMSO, and then the product **B22** was precipitated by the addition of ethanol, followed by filtration. Sharma et al. also utilized solventless synthesis using a ball mill as a reactor; in the case of basic conditions, the same type of activated starch was used, while for acidic conditions, sulfamic acid was added as a solid catalyst. The obtained mixtures were dissolved in water, and then ethanol was added to precipitate and isolate product **B22**.[59]

It was established that glycine betaine monohydrate can be used in esterification reactions as well. This method was proposed in 2020 by Duan et al., who successfully esterified zwitterions of betaine **18** with the use of cellulose from cotton fabric (**Figure S24** and **Table S2**, No. 140).

In this method, a few pieces of cotton fabric were introduced into an aqueous solution of **18,** and then hydrochloric acid was added to decrease the pH of the obtained solution. After vigorous stirring, the fabric pieces were removed, dried and heated at high temperature. In effect, a fabric with an immobilized betainate moiety **B30** was formed with high efficiency.[60]

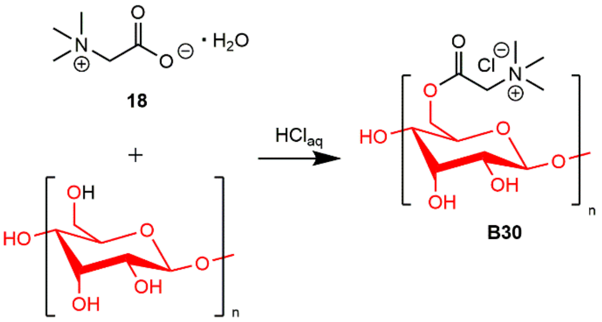

Figure S24. Direct esterification of zwitterion of betaine (more details are available in **Table S2**, No. 140).

3.5. Synthesis by *O*-alkylation of betaines

Another relatively simple solution leading to esters of betaines is the *O*-alkylation of zwitterions of betaines with the use of alkyl halides. This method perfectly adheres to the concept of *green chemistry* because esterquats are obtained without the generation of any byproducts (100% atom economy). Additionally, without the necessity of utilizing catalysts, such reactions proceed with high yields at relatively low temperatures.[62,63]

In 2017, Häckl et al. alkylated the derivative of glycine betaine called carnitine **19**, which naturally occurs in the environment. As demonstrated in **Figure S25** (and **Table S2**, No. 141-147), the *O*-alkylation of **19** was performed with the use of various bromoalkanes in acetonitrile under an argon atmosphere. After the reaction, the solvent was removed, and then products **B31** (R_1 = C_2H_5, C_4H_9, C_6H_{13}, C_8H_{17}, $C_{10}H_{21}$, $C_{12}H_{25}$, $C_{14}H_{29}$) were washed with diethyl ether to remove residual substrates and then dried under vacuum.[62]

A similar method was utilized in 2020 by Niemczak et al., who alkylated glycine betaine **20** with a homologous series of bromoalkanes in acetonitrile at elevated temperature (**Figure S25** and **Table S2**, No. 148). As a consequence of the elongation of the chain in the obtained esterquats, it was necessary to conduct the reaction at higher temperatures as well as for a longer period of time. After evaporation of solvent from postreaction mixtures, products **B6** (R_1 = C_2H_5, C_3H_7, C_4H_9, C_5H_{11}, C_6H_{13}, C_7H_{15}, C_8H_{17}, C_9H_{19}, $C_{10}H_{21}$, $C_{12}H_{25}$, $C_{14}H_{29}$, $C_{16}H_{33}$, $C_{18}H_{37}$) were thoroughly washed with ethyl acetate and dried.[63]

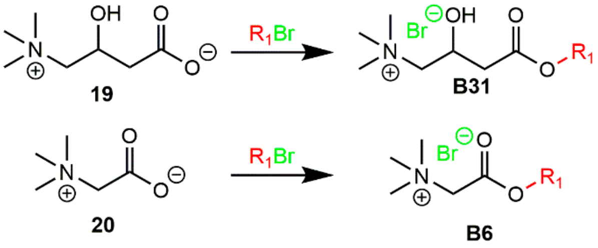

Figure S25. Direct *O*-alkylation of betaines in zwitterionic form (more details are available in **Table S2**, No. 141-148).

3.6. Synthesis of betaine-based esterquats from biologically active compounds

In this case, a direct acid-base reaction can also lead to undesired hydrolysis of the ester bond. Therefore, to avoid such a risk, an anion exchange reaction (**Figure S26**) was established as a relevant, efficient solution, but this approach suffers from the risk of unfavorable reactions, *e.g.,* transesterification.

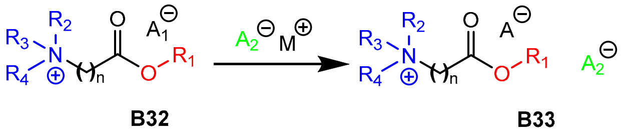

$A_1^{\ominus}$ = anion

$A_2^{\ominus}$ = any compatible anion

M = metal [Li, Na, K]

Figure S26. Synthesis of multifunctional betaine esterquats by anion exchange.

Recently, it was found that the structure of the anion utilized for ion exchange can significantly affect transesterification of esterquat with short chain alcohol used as a solvent. For example, in the case of alkyl betainate bromides (**B6**, n=1, R_1=alkyl chain from ethyl to octadecyl), anion exchange to herbicidally active sulfonylurea can be carried out in methanol,[63], while in the case of benzoate derivative (dicamba herbicide) ethanol had to be used instead of methanol to avoid transesterification.[64]

Table S2. Syntheses of betaine-type esterquats

No.	Stage	Substrate	R_1/R_2 or Reagent	Conditions	Product	Yield	Ref.
1	I	Chloroacetyl chloride (**10**, n = 1)	$C_{10}H_{21}$-	CH_2Cl_2, RT, 6 h	**E11**	97%	[41]
2	II	**E11**	Dimethylamine	Et_2O, RT, 72 h	**E12**	91%	
3	III	**E12**	4-nitrobenzyl bromide	Et_2O. NaOH, RT, 24 h	**B1**	53%	
4	I	Chloroacetyl chloride (**10**, n = 1)	$C_{10}H_{21}$-	CH_2Cl_2, 30 °C, 1 h	**E11**	NA	[34]
5	I	Chloroacetyl chloride (**10**, n = 1)	$C_{12}H_{25}$-	CH_2Cl_2, 30 °C, 1 h	**E11**	NA	
6	I	Chloroacetyl chloride (**10**, n = 1)	$C_{14}H_{29}$-	CH_2Cl_2, 30 °C, 1 h	**E11**	NA	
7	I	Chloroacetyl chloride (**10**, n = 1)	$C_{16}H_{33}$-	CH_2Cl_2, 30 °C, 1 h	**E11**	NA	
8	I	Chloroacetyl chloride (**10**, n = 1)	$C_{18}H_{37}$-	CH_2Cl_2, 30 °C, 1 h	**E11**	NA	
9	II	**E11**	Triethylamine	Acetone, 50 °C, 3 h	**B2**	NA	
10	I	Chlorobutanoyl chloride (**10**, n = 3)	Bn-($OOCC_5H_{10}$)n-	$CHCl_3$, Pyridine, N_2, 25 °C, 3h	**E11**	91%	[36]
11	I	Chlorobutanoyl chloride (**10**, n = 3)	$BnOOCC_5H_{10}$-	$CHCl_3$, Pyridine, N_2, 25 °C, 5h	**E11**	70%	
12	II	**E11** ($R_1 = C_6H_5O[C(=O)C_5H_{10}O]_n$-H)	Trimethylaminie	Acetonitrile, N_2, 60 °C, 45 h	**B3**	80%	
13	II	**E11** ($R_1 = C_6H_5OC(=O)C_5H_{10}OH$)	Trimethylaminie	Acetonitrile, N_2, 60 °C, 45 h	**B3**	95%	
14	I	Chloroacetyl chloride (**10**, n = 1)	$C_8H_{17}OH$	CH_2Cl_2, 50 °C, 8 h	**E11**	96%	[42]
15	I	Chloroacetyl chloride (**10**, n = 1)	$C_{10}H_{21}OH$	CH_2Cl_2, 50 °C, 8 h	**E11**	96%	
16	I	Chloroacetyl chloride (**10**, n = 1)	$C_{12}H_{25}OH$	CH_2Cl_2, 50 °C, 8 h	**E11**	97%	
17	II	**E11** ($R_1 = C_8H_{17}$)	Bis(3-(dimethylamino)propyl)succinamide	Ethyl acetate, 80 °C, 24 h	**B4**	75%	
18	II	**E11** ($R_1 = C_{10}H_{21}$)	Bis(3-(dimethylamino)propyl)succinamide	Ethyl acetate, 80 °C, 24 h	**B4**	81%	
19	II	**E11** ($R_1 = C_{12}H_{25}$)	Bis(3-(dimethylamino)propyl)succinamide	Ethyl acetate, 80 °C, 24 h	**B4**	82%	
20	II	**E11** ($R_1 = C_8H_{17}$)	Bis(3-(dimethylamino)propyl)glutaramide	Ethyl acetate, 80 °C, 24 h	**B4**	80%	
21	II	**E11** ($R_1 = C_{10}H_{21}$)	Bis(3-(dimethylamino)propyl)glutaramide	Ethyl acetate, 80 °C, 24 h	**B4**	82%	
22	II	**E11** ($R_1 = C_{12}H_{25}$)	Bis(3-(dimethylamino)propyl)glutaramide	Ethyl acetate, 80 °C, 24 h	**B4**	85%	
23	II	**E11** ($R_1 = C_8H_{17}$)	Bis(3-(dimethylamino)propyl)adipamide	Ethyl acetate, 80 °C, 24 h	**B4**	83%	
24	II	**E11** ($R_1 = C_{10}H_{21}$)	Bis(3-(dimethylamino)propyl)adipamide	Ethyl acetate, 80 °C, 24 h	**B4**	85%	
25	II	**E11** ($R_1 = C_{12}H_{25}$)	Bis(3-(dimethylamino)propyl)adipamide	Ethyl acetate, 80 °C, 24 h	**B4**	88%	

26	I	Chloroacetyl chloride (**10**, n = 1)	Ethylene glycol	N_2, 50 °C, 8 h	**E13**	65%	38
27	II	**E13**	$C_{12}H_{25}$-	Ethyl acetate, 77 °C, 10 h	**B5**	79%	
28	II	**E13**	$C_{16}H_{33}$-	Ethyl acetate, 77 °C, 10 h	**B5**	79%	
29	I	Bromoacetyl bromide (**11**, n = 1)	$C_{12}H_{25}$-	CH_2Cl_2, 21 °C, 4 h	**E14**	94%	13
30	II	**E14**	Trimethylamine	Acetone, 21 °C, 5 h	**B6**	85%	
31	II	**E14**	Tetramethyl ethylenediamine	Acetone, 56 °C, 20 h	**B7**	83%	
32	I	Bromoacetyl bromide (**11**, n = 1)	$C_{12}H_{25}$	CH_2Cl_2, N,N-diisopropylethylamine, 25 °C, 20 h	**E14**	70%	39
33	II	**E14**	Methyl hyperbranched polyethylenediamine	DMF, 25 °C, 48 h	**B8**	74%	
34	I	Bromoacetyl bromide (**11**, n = 1)	Triethanolamine	CH_2Cl_2, K_2CO_3, 25 °C, 5 h	**E15**	55%	40
35	II	**E15**	$C_{10}H_{21}$-	Acetone, 56 °C, 6 h	**B9**	50%	
36	II	**E15**	$C_{12}H_{25}$-	Acetone, 56 °C, 6 h	**B9**	58%	
37	II	**E15**	$C_{14}H_{29}$-	Acetone, 56 °C, 6 h	**B9**	67%	
38	II	**E15**	$C_{16}H_{33}$-	Acetone, 56 °C, 6 h	**B9**	80%	
39	I	Methyl bromoacetate (**12**, n = 1, m = 0)	CH_2=CHCONH$(CH_2)_3$N+$(CH_3)_2CH_2COOCH_3$	Acetonitrile, N_2 or Ar, 25 °C, 6 h	**B10**	96%	43
40	I	Ethyl bromobutyrate (**12**, n = 3, m = 1)	CH_2=CHCONH$(CH_2)_3$N+$(CH_3)_2CH_2COOCH_3$	Acetonitrile, N_2 or Ar, 25 °C, 72 h	**B10**	92%	
41	I	Ethyl bromocapronate (**12**, n = 5, m = 1)	CH_2=CHCONH$(CH_2)_3$N+$(CH_3)_2CH_2COOCH_3$	Acetonitrile, N_2 or Ar, 25 °C, 120 h	**B10**	87%	
42	I	Ethyl bromoacetate (**12**, n = 1, m = 1)	C_2H_5-	Ethyl acetate, 21 °C, 24 h	**B11**	86%	44
43	I	Ethyl bromoacetate (**12**, n = 1, m = 1)	C_3H_7-	Ethyl acetate, 21 °C, 24 h	**B11**	80%	
44	I	Ethyl bromoacetate (**12**, n = 1, m = 1)	C_4H_9-	Ethyl acetate, 21 °C, 24 h	**B11**	85%	
45	I	Ethyl bromoacetate (**12**, n = 1, m = 1)	*cyclo*-C_4H_8-	Ethyl acetate, 21 °C, 24 h	**B12**	80%	
46	I	Methyl bromoacetate (**12**, n = 1, m = 0)	*cyclo*-$CH_2N(CH_3)C_2H_4$-	Acetonitrile, N_2, 40 °C, 2 h	**B12**	NA	45
47	I	Ethyl bromoacetate (**12**, n = 1, m = 1)	*cyclo*-$C_2H_4OC_2H_4$-	Acetonitrile, N_2, 40 °C, 2 h	**B12**	NA	
48	I	Ethyl bromoacetate (**12**, n = 1, m = 1)	*cyclo*-C_5H_{10}-	Acetonitrile, N_2, 40 °C, 2 h	**B12**	NA	
49	I	Ethyl bromoacetate (**12**, n = 1, m = 1)	*cyclo*-C_4H_8-	Acetonitrile, N_2, 40 °C, 2 h	**B12**	93%	
50	I	Hexyl bromoacetate (**12**, n = 1, m = 5)	$C_6H_5CH_2$	Acetonitrile, reflux, 2 h	**B10**	99%	46

51	I	Hexyl bromoacetate (**12**, n = 1, m = 5)	Tetramethylenediamine	Acetonitrile, reflux, 1 h	**B7**	75%
52	I	Hexyl bromoacetate (**12**, n = 1, m = 5)	1-(2-dimethhylaminoethyl)-4-methylpiperazine	Acetonitrile, RT, 12 h	**B13**	63%
53	I	Hexyl bromoacetate (**12**, n = 1, m = 5)	2,6,10-trimethyl-2,6,10-triazaundecane	Acetonitrile, RT, 72 h	**B14**	76%
54	I	Hexyl bromoacetate (**12**, n = 1, m = 5)	tris(N,N-dimethylaminopropyl)amine	Acetonitrile, reflux, 3 h	**B15**	99%
55	I	Heptyl bromoacetate (**12**, n = 1, m = 6)	$C_6H_5CH_2$	Acetonitrile, reflux, 2 h	**B10**	92%
56	I	Heptyl bromoacetate (**12**, n = 1, m = 6)	Tetramethylenediamine	Acetonitrile, reflux, 1 h	**B7**	77%
57	I	Heptyl bromoacetate (**12**, n = 1, m = 6)	1-(2-dimethhylaminoethyl)-4-methylpiperazine	Acetonitrile, RT, 12 h	**B13**	74%
58	I	Heptyl bromoacetate (**12**, n = 1, m = 6)	2,6,10-trimethyl-2,6,10-triazaundecane	Acetonitrile, RT, 72 h	**B14**	92%
59	I	Heptyl bromoacetate (**12**, n = 1, m = 6)	tris(N,N-dimethylaminopropyl)amine	Acetonitrile, reflux, 3 h	**B15**	99%
60	I	Octyl bromoacetate (**12**, n = 1, m = 7)	$C_6H_5CH_2$	Acetonitrile, reflux, 2 h	**B10**	99%
61	I	Octyl bromoacetate (**12**, n = 1, m = 7)	Tetramethylenediamine	Acetonitrile, reflux, 1 h	**B7**	68%
62	I	Octyl bromoacetate (**12**, n = 1, m = 7)	1-(2-dimethhylaminoethyl)-4-methylpiperazine	Acetonitrile, RT, 12 h	**B13**	92%
63	I	Octyl bromoacetate (**12**, n = 1, m = 7)	2,6,10-trimethyl-2,6,10-triazaundecane	Acetonitrile, RT, 72 h	**B14**	81%
64	I	Octyl bromoacetate (**12**, n = 1, m = 7)	tris(N,N-dimethylaminopropyl)amine	Acetonitrile, reflux, 3 h	**B15**	99%
65	I	Nonyl bromoacetate (**12**, n = 1, m = 8)	$C_6H_5CH_2$	Acetonitrile, reflux, 2 h	**B10**	95%
66	I	Nonyl bromoacetate (**12**, n = 1, m = 8)	Tetramethylenediamine	Acetonitrile, reflux, 1 h	**B7**	77%
67	I	Nonyl bromoacetate (**12**, n = 1, m = 8)	1-(2-dimethhylaminoethyl)-4-methylpiperazine	Acetonitrile, RT, 12 h	**B13**	96%
68	I	Nonyl bromoacetate (**12**, n = 1, m = 8)	2,6,10-trimethyl-2,6,10-triazaundecane	Acetonitrile, RT, 72 h	**B14**	85%
69	I	Nonyl bromoacetate (**12**, n = 1, m = 8)	tris(N,N-dimethylaminopropyl)amine	Acetonitrile, reflux, 3 h	**B15**	99%
70	I	Decyl bromoacetate (**12**, n = 1, m = 9)	$C_6H_5CH_2$	Acetonitrile, reflux, 2 h	**B10**	99%
71	I	Decyl bromoacetate (**12**, n = 1, m = 9)	Tetramethylenediamine	Acetonitrile, reflux, 1 h	**B7**	81%
72	I	Decyl bromoacetate (**12**, n = 1, m = 9)	1-(2-dimethhylaminoethyl)-4-methylpiperazine	Acetonitrile, RT, 12 h	**B13**	82%
73	I	Decyl bromoacetate (**12**, n = 1, m = 9)	2,6,10-trimethyl-2,6,10-triazaundecane	Acetonitrile, RT, 72 h	**B14**	90%
74	I	Decyl bromoacetate (**12**, n = 1, m = 9)	tris(N,N-dimethylaminopropyl)amine	Acetonitrile, reflux, 3 h	**B15**	93%
75	I	Dodecyl bromoacetate (**12**, n = 1, m = 11)	$C_6H_5CH_2$	Acetonitrile, reflux, 2 h	**B10**	91%
76	I	Dodecyl bromoacetate (**12**, n = 1, m = 11)	Tetramethylenediamine	Acetonitrile, reflux, 1 h	**B7**	78%

77	I	Dodecyl bromoacetate (**12**, n = 1, m = 11)	1-(2-dimethhylaminoethyl)-4-methylpiperazine	Acetonitrile, RT, 12 h	**B13**	90%	
78	I	Dodecyl bromoacetate (**12**, n = 1, m = 11)	2,6,10-trimethyl-2,6,10-triazaundecane	Acetonitrile, RT, 72 h	**B14**	91%	
79	I	Dodecyl bromoacetate (**12**, n = 1, m = 11)	tris(N,N-dimethylaminopropyl)amine	Acetonitrile, reflux, 3 h	**B15**	83%	
80	I	Tetradecyl bromoacetate (**12**, n = 1, m = 13)	$C_6H_5CH_2$	Acetonitrile, reflux, 2 h	**B10**	98%	
81	I	Tetradecyl bromoacetate (**12**, n = 1, m = 13)	Tetramethylenediamine	Acetonitrile, reflux, 1 h	**B7**	88%	
82	I	Tetradecyl bromoacetate (**12**, n = 1, m = 13)	1-(2-dimethhylaminoethyl)-4-methylpiperazine	Acetonitrile, RT, 12 h	**B13**	97%	
83	I	Tetradecyl bromoacetate (**12**, n = 1, m = 13)	2,6,10-trimethyl-2,6,10-triazaundecane	Acetonitrile, RT, 72 h	**B14**	95%	
84	I	Tetradecyl bromoacetate (**12**, n = 1, m = 13)	tris(N,N-dimethylaminopropyl)amine	Acetonitrile, reflux, 3 h	**B15**	92%	
85	I	Methyl bromoundecanoate (**12**, n = 10, m = 0)	C_6H_{13}-	DMF, K_2CO_3, 90-110 °C, 12 h	**E16**	NA	47
86	I	Methyl bromoundecanoate (**12**, n = 10, m = 0)	C_6H_{13}-	DMF, K_2CO_3, 90-110 °C, 12 h	**E17**	NA	
87	I	Methyl bromoundecanoate (**12**, n = 10, m = 0)	$C_{12}H_{25}$-	DMF, K_2CO_3, 90-110 °C, 12 h	**E16**	NA	
88	I	Methyl bromoundecanoate (**12**, n = 10, m = 0)	$C_{12}H_{25}$-	DMF, K_2CO_3, 90-110 °C, 12 h	**E17**	NA	
89	I	Methyl bromoundecanoate (**12**, n = 10, m = 0)	$C_{18}H_{37}$-	DMF, K_2CO_3, 90-110 °C, 12 h	**E16**	NA	
90	I	Methyl bromoundecanoate (**12**, n = 10, m = 0)	$C_{18}H_{37}$-	DMF, K_2CO_3, 90-110 °C, 12 h	**E17**	NA	
91	I	Methyl bromoundecanoate (**12**, n = 10, m = 0)	C_8H_{17}-	DMF, K_2CO_3, 90-110 °C, 12 h	**E18**	NA	
92	I	Methyl bromoundecanoate (**12**, n = 10, m = 0)	c-C_6H_{11}-<br>c-C_6H_{11}-	DMF, K_2CO_3, 90-110 °C, 12 h	**E18**	NA	
93	II	**E16**	CH_3I	$CHCl_3$, K_2CO_3, 25 °C, 12 h	**B16**	NA	
94	II	**E17**	CH_3I	$CHCl_3$, K_2CO_3, 25 °C, 12 h	**B17**	NA	
95	II	**E18**	CH_3I	$CHCl_3$, K_2CO_3, 25 °C, 12 h	**B18**	NA	

96	I	Monoethylene glycol bromoacetate (**13**, m = 1)	$C_{12}H_{25}$-	$CHCl_3$, 60 °C, 3 h	**B19**	77%	48
97	I	Diethylene glycol bromoacetate (**13**, m = 2)	$C_{12}H_{25}$-	$CHCl_3$, 60 °C, 3 h	**B19**	80%	
98	I	Triethylene glycol bromoacetate (**13**, m = 3)	$C_{12}H_{25}$-	$CHCl_3$, 60 °C, 3 h	**B19**	82%	
99	I	Monoethylene glycol bromoacetate (**13**, m = 1)	C_8H_{17}-	$CHCl_3$, 60 °C, 3 h	**B19**	35%	49
100	I	Monoethylene glycol bromoacetate (**13**, m = 1)	$C_{10}H_{21}$-	$CHCl_3$, 60 °C, 3 h	**B19**	49%	
101	I	Monoethylene glycol bromoacetate (**13**, m = 1)	$C_{12}H_{25}$-	$CHCl_3$, 60 °C, 3 h	**B19**	66%	
102	I	Monoethylene glycol bromoacetate (**13**, m = 1)	$C_{14}H_{29}$-	$CHCl_3$, 60 °C, 3 h	**B19**	74%	
103	I	Monoethylene glycol bromoacetate (**13**, m = 1)	$C_{16}H_{33}$-	$CHCl_3$, 60 °C, 3 h	**B19**	65%	
104	I	Monoethylene glycol bromoacetate (**13**, m = 1)	$C_{18}H_{37}$-	$CHCl_3$, 60 °C, 3 h	**B19**	57%	
105	I	Octyl chloroacetate (**14**, m = 7)	PMDTA	Acetonitrile, 82 °C, 60 h	**B20**	85%	50
106	I	Octyl chloroacetate (**14**, m = 7)	PMDTA	Acetonitrile, 82 °C, 60 h	**B21**	89%	
107	I	Decyl chloroacetate (**14**, m = 9)	PMDTA	Acetonitrile, 82 °C, 60 h	**B20**	86%	
108	I	Decyl chloroacetate (**14**, m = 9)	PMDTA	Acetonitrile, 82 °C, 60 h	**B21**	90%	
109	I	Dodecyl chloroacetate (**14**, m = 11)	PMDTA	Acetonitrile, 82 °C, 60 h	**B21**	85%	
110	I	Chlorobetainyl chloride (**15**)	Starch	Pyridine, dioxane, 110 °C, 4 h	**B22**	NA	52
111	I	Chlorobetainyl chloride (**15**)	Cellulose	Dimethylacetamide, LiCl, Pyridine, 40 °C, 48 h	**B23**	NA	53
112	I	Betaine (**16**, $R_2 = CH_3$)	$C_{12}H_{25}$-	Methanesulfonic acid, 130 °C, reduced pressure (10 mbar), 3 h	**B24**	95%	55
113	I	Betaine (**16**, $R_2 = CH_3$)	$C_{18}H_{37}$-	Methanesulfonic acid, 130 °C, reduced pressure (50 mbar), 7 h	**B24**	70%	

114	I	Betaine (**16**, R_2 = CH_3)	$C_{18}H_{35}$-	Methanesulfonic acid, 130 °C, reduced pressure (50 mbar), 7 h	**B24**	85%	
115	I	Betaine (**16**, R_2 = CH_3)	C_6H_{13}-	Methanesulfonic acid, 160 °C, 24 h	**B24**	95%	56
116	I	Betaine (**16**, R_2 = CH_3)	$C_{12}H_{25}$-	Methanesulfonic acid, 140 °C, reduced pressure (50-100 mbar), 7 h	**B24**	86%	
117	I	Betaine (**16**, R_2 = CH_3)	$C_{14}H_{29}$-	Methanesulfonic acid, 140 °C, reduced pressure (50-100 mbar), 7 h	**B24**	93%	
118	I	Betaine (**16**, R_2 = CH_3)	$C_{16}H_{33}$-	Methanesulfonic acid, 140 °C, reduced pressure (50-100 mbar), 7 h	**B24**	61%	
119	I	Betaine (**16**, R_2 = CH_3)	$C_{20}H_{41}$-	Methanesulfonic acid, 140 °C, reduced pressure (50 mbar), 17 h	**B24**	83%	
120	I	Betaine (**16**, R_2 = CH_3)	C_4H_9-	Methanesulfonic acid, 140 °C, 500 mbar, 3 h	**B24**	55%	57
121	II	**B24**	$C_{14}H_{29}$-	$NaHCO_3$, 85 °C, 10 mbar, 3 h	**B25**	100%	
122	II	**B24**	$C_{14}H_{29}$-	Methanesulfonic acid, 85 °C, 10 mbar, 3 h	**B25**	36%	
123	I	Betaine methanesulfonate (**16**, R_1 = CH_3)	Glycerol	150 °C, 1.5 h	**B26**	NA	58
124	I	Betaine methanesulfonate (**16**, R_1 = CH_3)	Glycerol	150 °C, 1.5 h	**B27**	NA	
125	I	Betaine sulfate (**16**, R_1 = OH)	Glycerol	150 °C, 2.5 h	**B26**	NA	
126	I	Betaine sulfate (**16**, R_1 = OH)	Glycerol	150 °C, 2.5 h	**B27**	NA	
127	I	Betaine trifluoromethanesulfonate (**16**, R_1 = CF_3)	Glycerol	150 °C, 2 h	**B26**	NA	
128	I	Betaine trifluoromethanesulfonate (**16**, R_1 = CF_3)	Glycerol	150 °C, 2 h	**B27**	NA	

129	I	Betaine p-toluenesulfonate (**16**, R_1 = *4*-$CH_3C_6H_4$)	Glycerol	150 °C, 2 h	**B26**	NA	
130	I	Betaine p-toluenesulfonate (**16**, R_1 = *4*-$CH_3C_6H_4$)	Glycerol	150 °C, 2 h	**B27**	NA	
131	I	Betaine hydrochloride (**17**)	Glycerol	150 °C, 10 h	**B28**	NA	
132	I	Betaine hydrochloride (**17**)	Glycerol	150 °C, 10 h	**B29**	NA	
133	I	Betaine hydrochloride (**17**)	Methanol	$SOCl_2$, 70 °C, 4 h	**B3**	NA	[59]
134	II	**B3**	Starch	NaOH, DMF, 70 °C, 24 h	**B22**	NA	
135	II	**B3**	Starch	NaOH, DMSO, 70 °C, 24 h	**B22**	NA	
136	II	**B3**	Starch	H_2SO_4, DMF, 70 °C, 24 h	**B22**	NA	
137	II	**B3**	Starch	H_2SO_4, DMSO, 70 °C, 24 h	**B22**	NA	
138	II	**B3**	Starch	NaOH, ball mill, 2h	**B22**	NA	
139	II	**B3**	Starch	Sulfamic acid, ball mill, 2 h	**B22**	NA	
140	I	Betaine (**18**)	Cellulose (cotton fiber)	H_2O, HCl, 180 °C, 5 min	**B30**	NA	[65]
141	I	Carnitine (**19**)	C_2H_5-	Acetonitrile, Ar, 81 °C, 12 h	**B25**	NA	[62]
142	I	Carnitine (**19**)	C_4H_9-	Acetonitrile, Ar, 81 °C, 12 h	**B25**	NA	
143	I	Carnitine (**19**)	C_6H_{13}-	Acetonitrile, Ar, 81 °C, 12 h	**B25**	NA	
144	I	Carnitine (**19**)	C_8H_{17}-	Acetonitrile, Ar, 81 °C, 12 h	**B25**	NA	
145	I	Carnitine (**19**)	$C_{10}H_{21}$-	Acetonitrile, Ar, 81 °C, 12 h	**B25**	NA	
146	I	Carnitine (**19**)	$C_{12}H_{25}$-	Acetonitrile, Ar, 81 °C, 12 h	**B25**	NA	
147	I	Carnitine (**19**)	$C_{14}H_{29}$-	Acetonitrile, Ar, 81 °C, 12 h	**B25**	NA	
148	I	Betaine (**19**)	Alkyl from C_2H_5- to $C_{14}H_{29}$-	Acetonitrile, 40 °C, 24-72 h	**B4**	87-95%	[63]

4.1. Structures of compounds (hydrolysis)

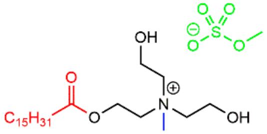

1

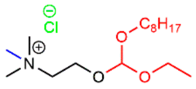

2

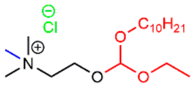

3 - 5

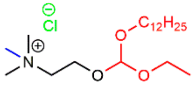

6 - 8

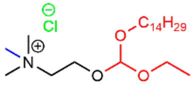

9

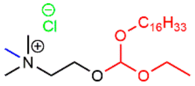

10

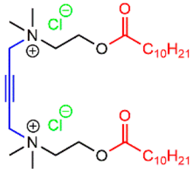

11, 12

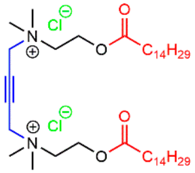

13 - 16

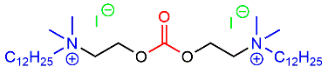

17 - 22

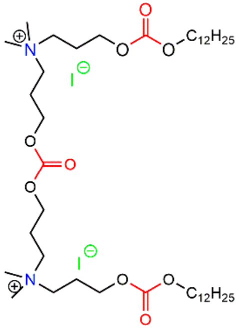

23-25

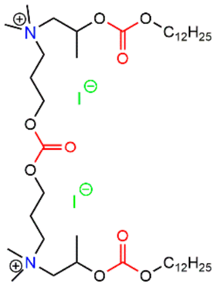

26-28

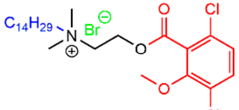

29. 30

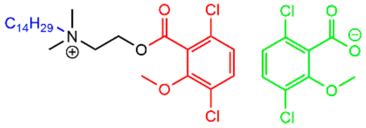

31, 32

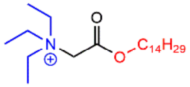

33-42

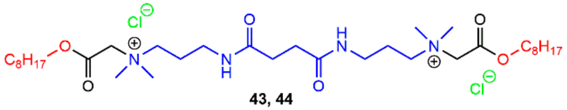

43, 44

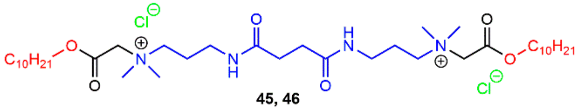

45, 46

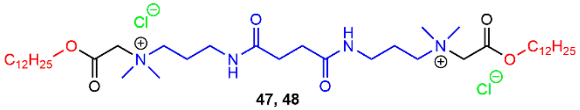

47, 48

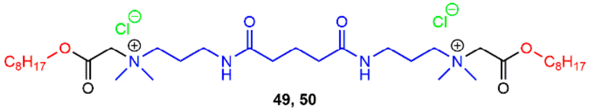

49, 50

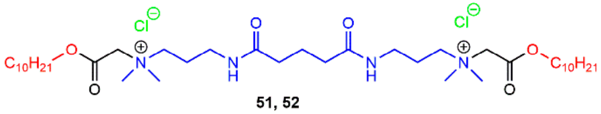

51, 52

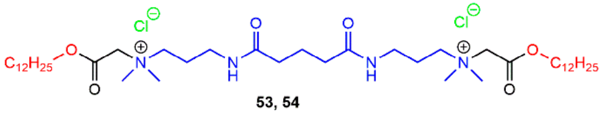

53, 54

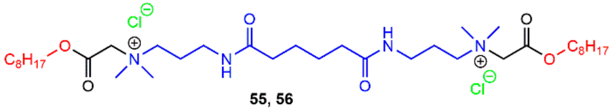

55, 56

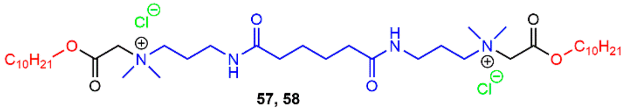

57, 58

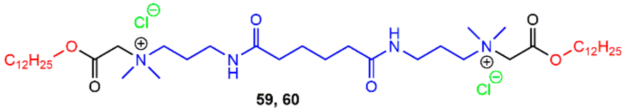

59, 60

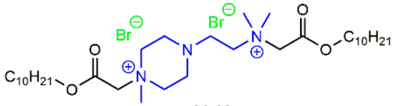

61-63

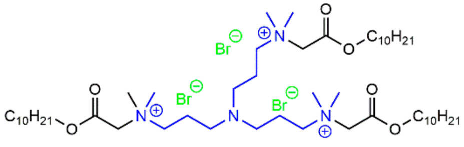

64-66

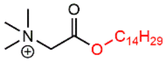

67-72

4.2. Structures of compounds (biodegradation)

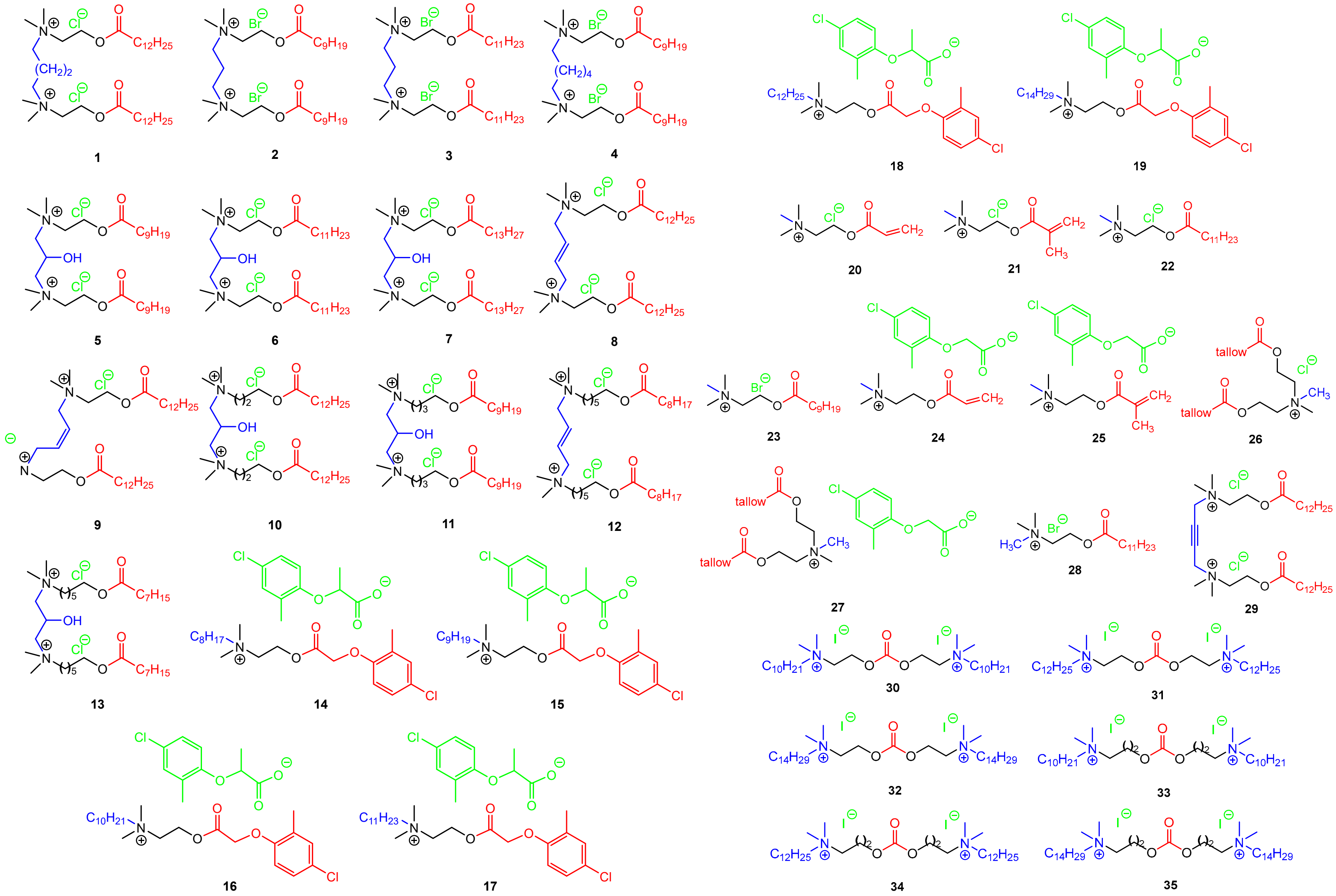

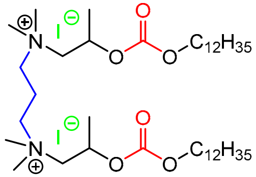

36

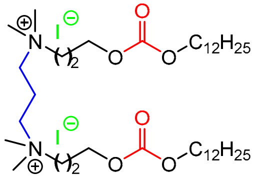

37

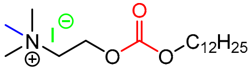

38

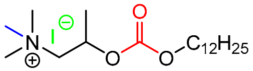

39

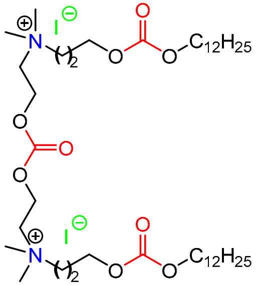

40

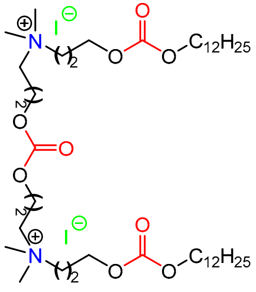

41

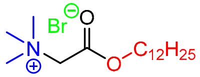

36

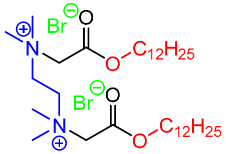

42

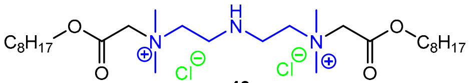

43

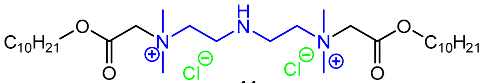

44

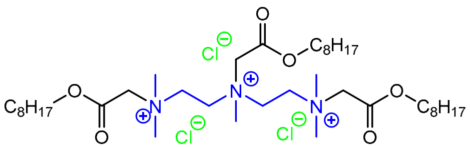

45

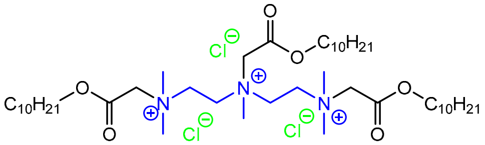

46

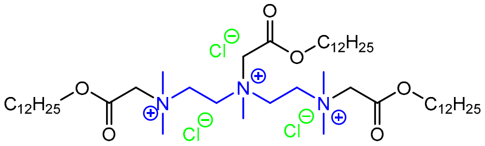

47

4.3. Structures of compounds (antimicrobial properties)

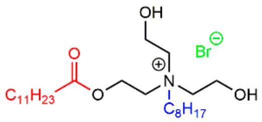

1

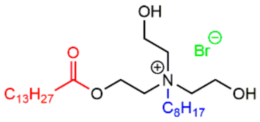

2

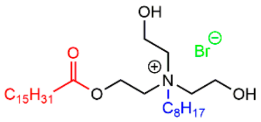

3

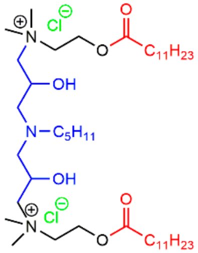

4

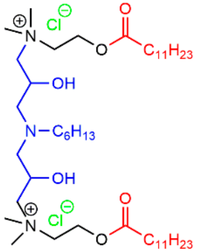

5

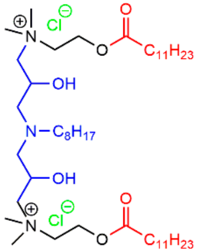

6

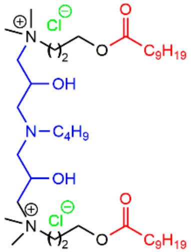

7

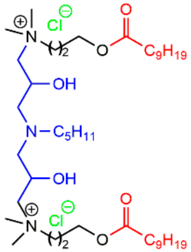

8

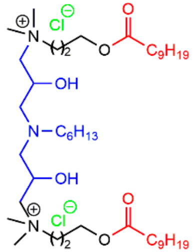

9

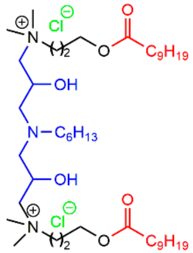

10

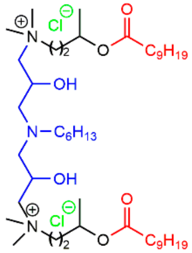

11

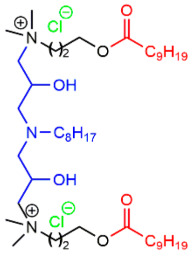

12

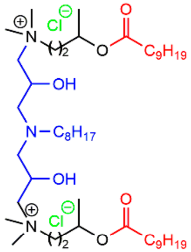

13

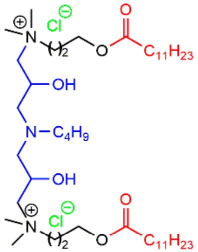

14

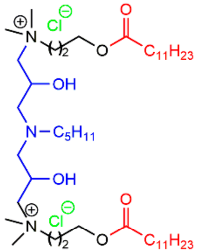

15

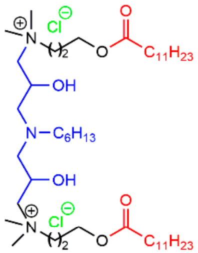

16

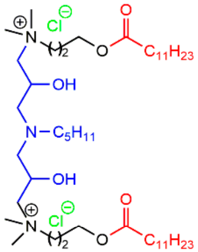

17

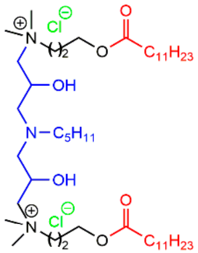

18

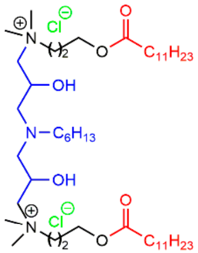

19

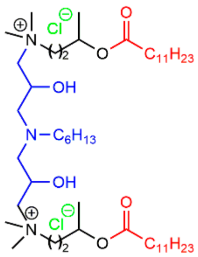

20

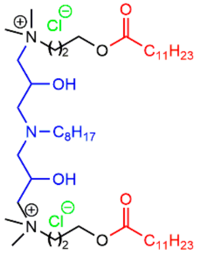

21

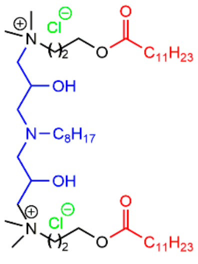

22

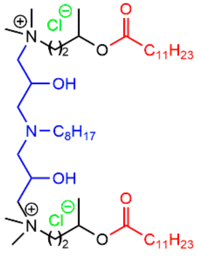

23

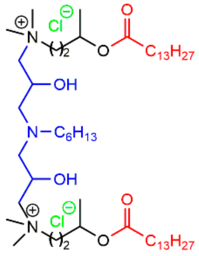

24

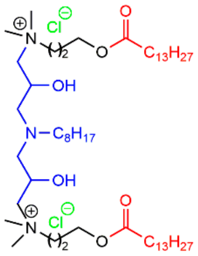

25

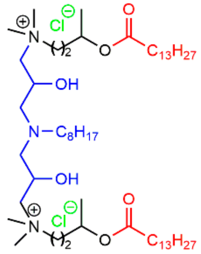

26

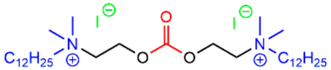
27
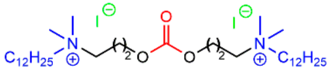
28
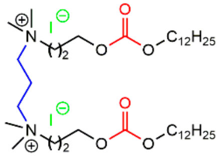
29
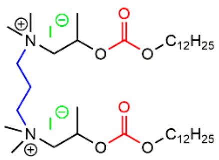
30
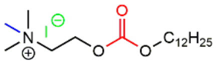
31
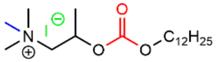
32
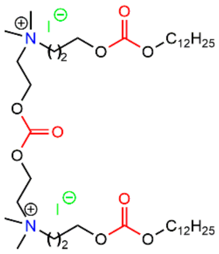
33
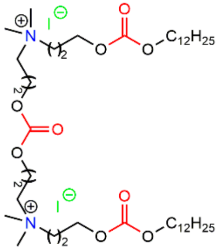
34
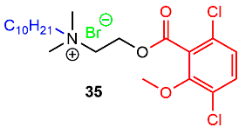
35
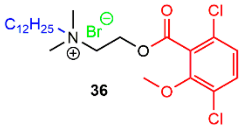
36
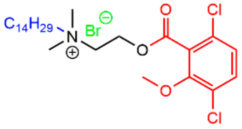
37
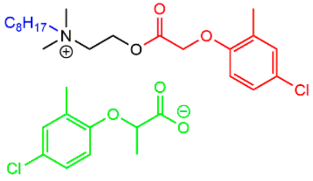
38
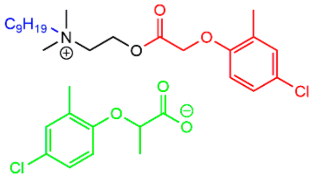
39
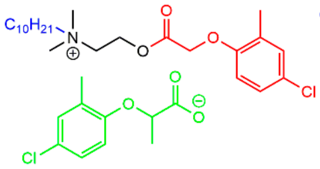
40
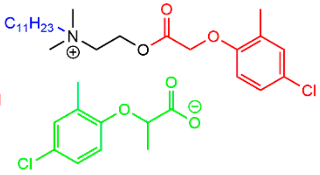
41
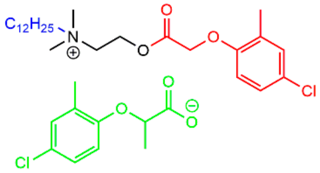
42
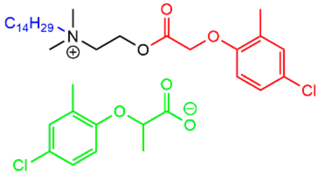
43
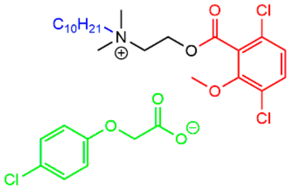
44
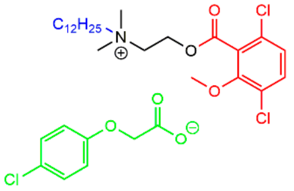
45
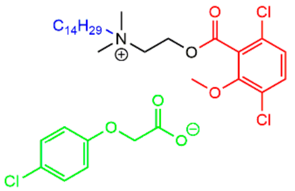
46
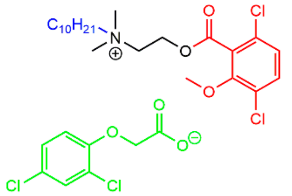
47
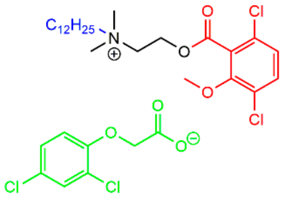
48
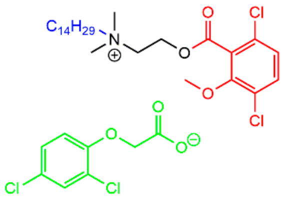
49
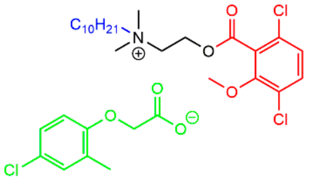
50
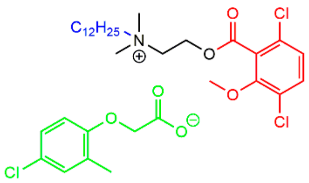
51
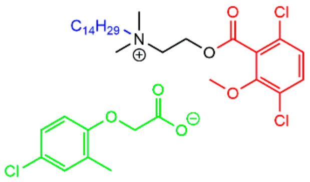
52

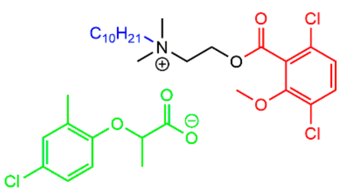

53

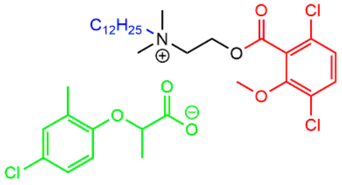

54

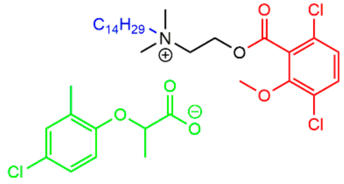

55

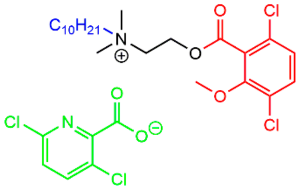

56

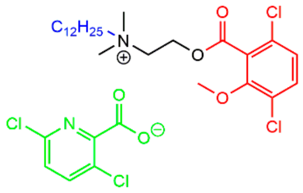

57

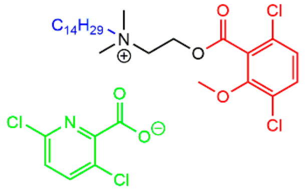

58

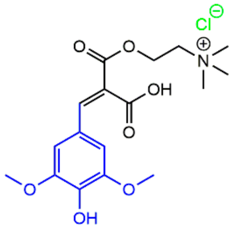

59

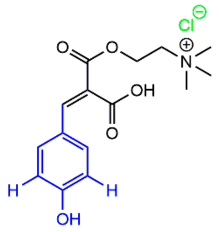

60

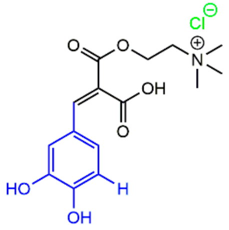

61

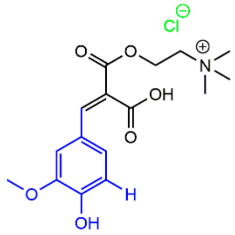

62

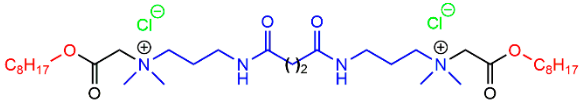

63

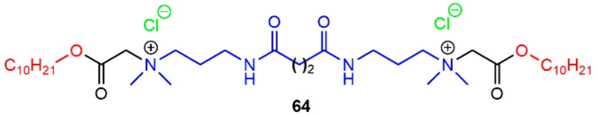

64

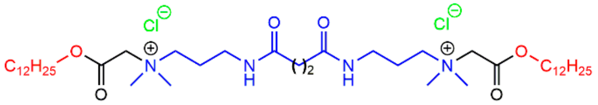

65

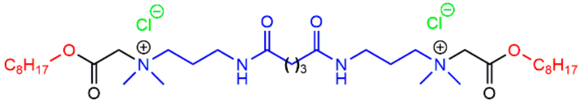

66

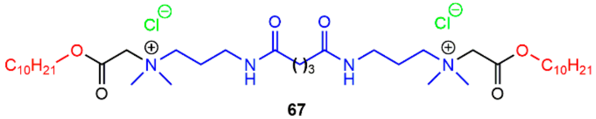

67

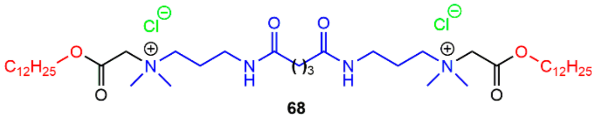

68

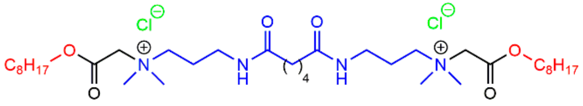

69

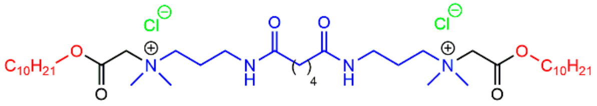

70

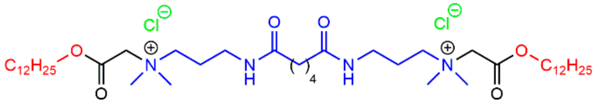

71

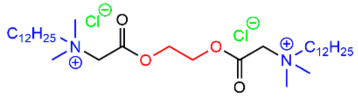

72

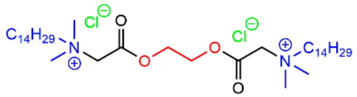

73

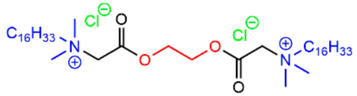

74

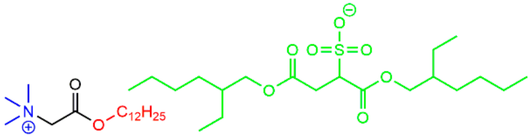

75

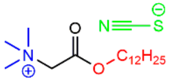

76

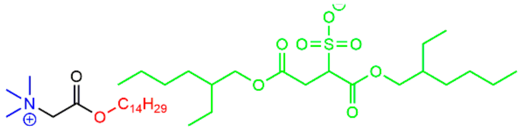

77

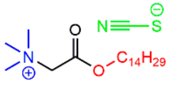

78

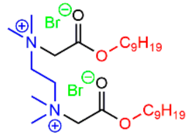

79

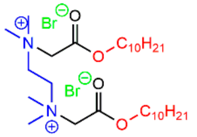

80

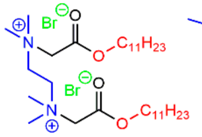

81

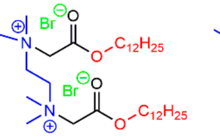

82

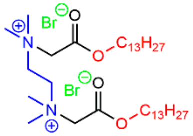

83

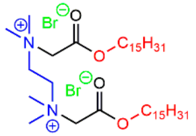

84

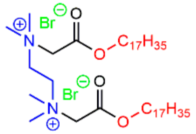

85

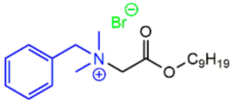

86

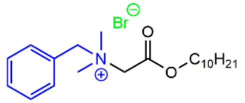

87

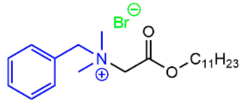

88

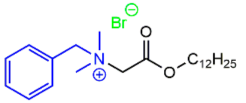

89

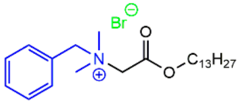

90

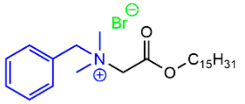

91

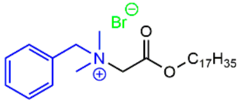

92

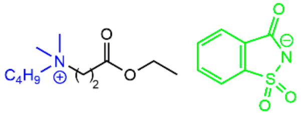

93

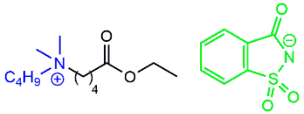

94

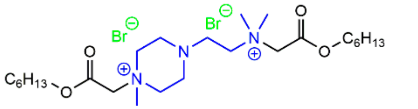

95

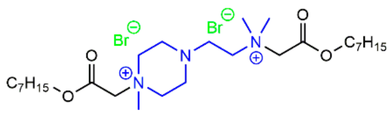

96

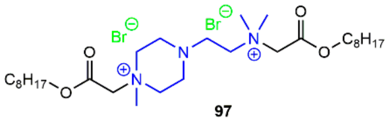

97

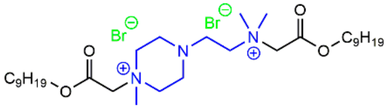

98

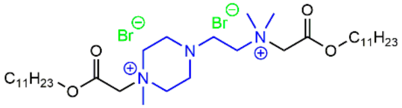

99

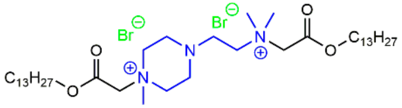

100

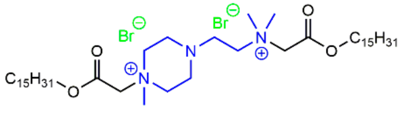

101

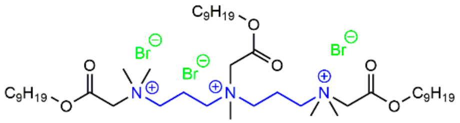

102

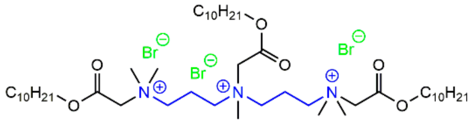

103

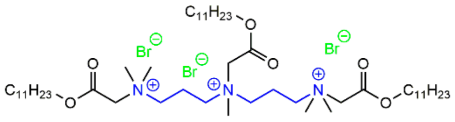

104

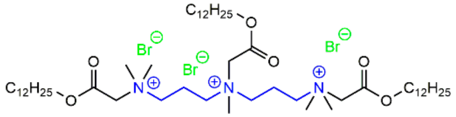

105

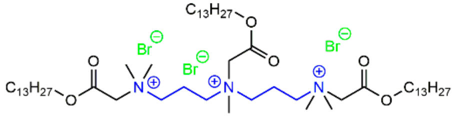

106

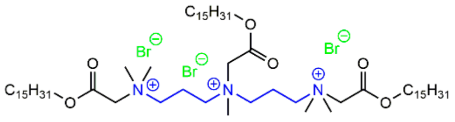

107

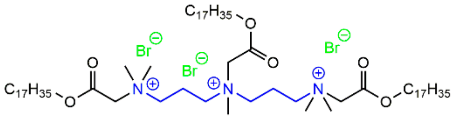

108

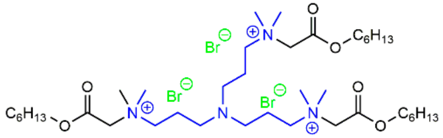

109

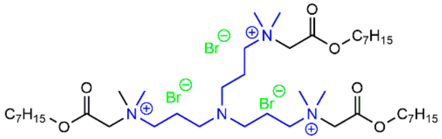

110

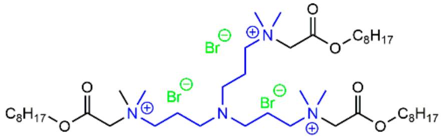

111

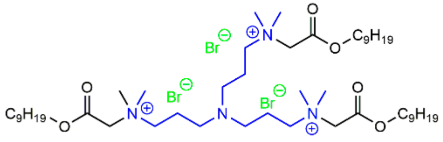

112

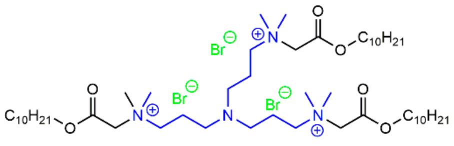

113

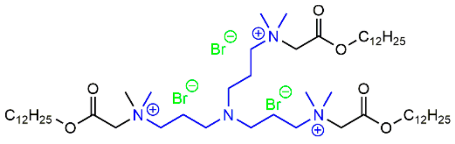

114

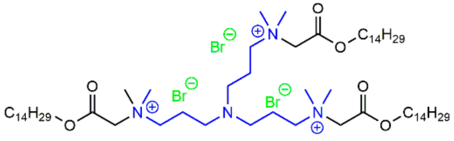

115

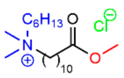

116

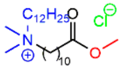

117

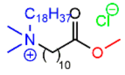

118

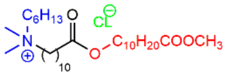

119

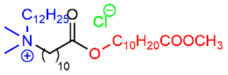

120

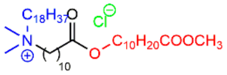

121

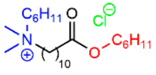

122

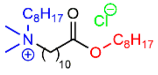

123

4.4. Structures of compounds (toxicity)

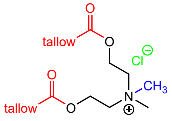

1

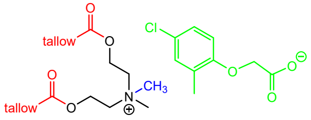

2

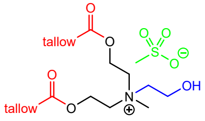

3

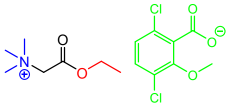

4

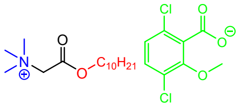

5

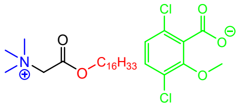

6

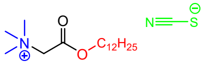

7

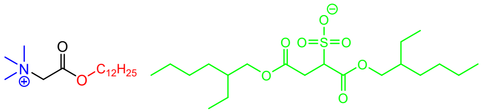

8

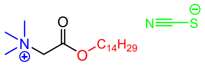

9

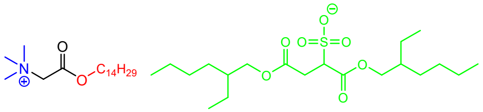

10

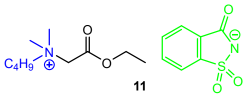

11

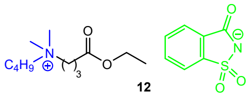

12

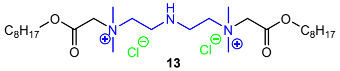

13

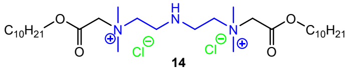

14

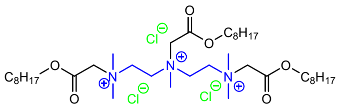

15

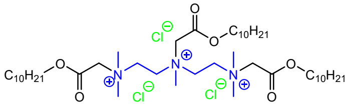

16

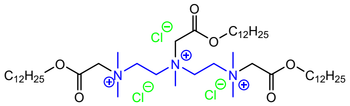

17

4.5. Structures of compounds (surface properties)

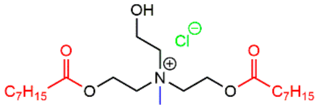
1

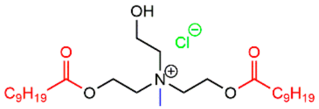
2

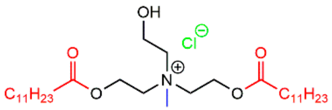
3

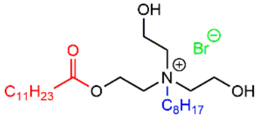
4

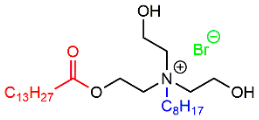
5

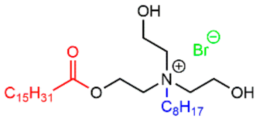
6

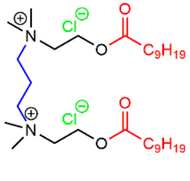
7

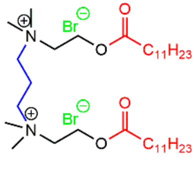
8

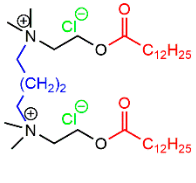
9

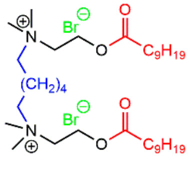
10

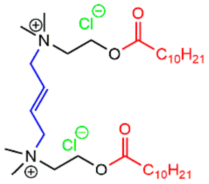
11

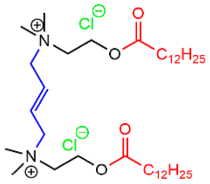
12

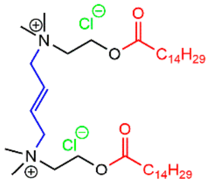
13

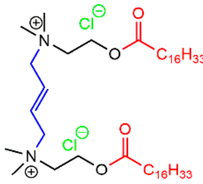
14

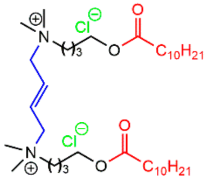
15

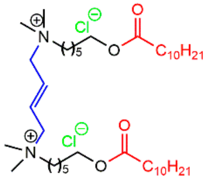
16

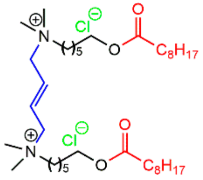
17

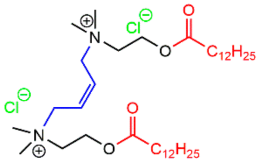
18

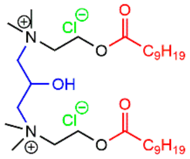
19

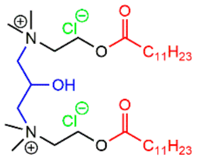
20

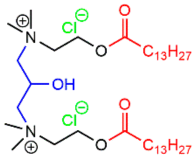
21

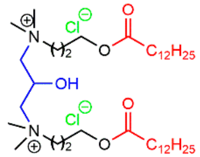
22

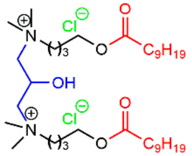
23

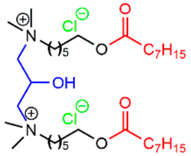
24

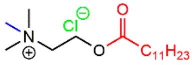
25

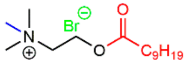
26

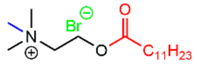
27

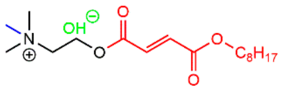
28

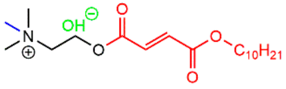
29

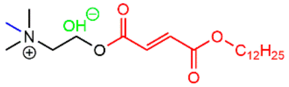
30

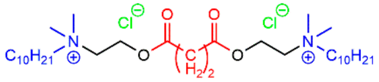
31

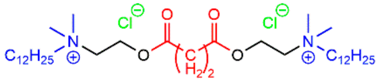
32

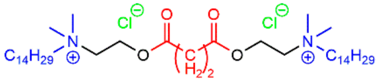
33

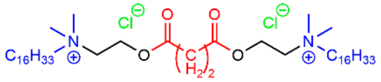
34

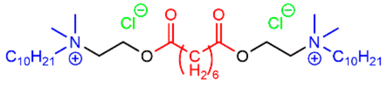
35

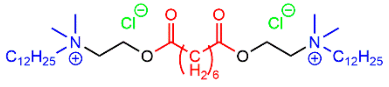
3
6

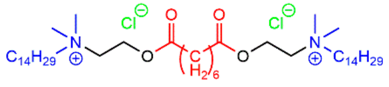
37

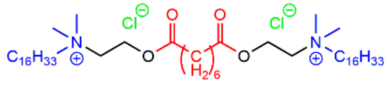
38

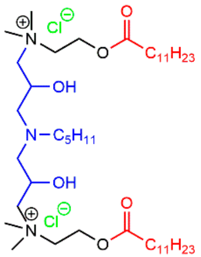
39

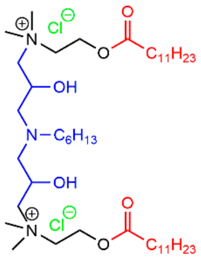
40

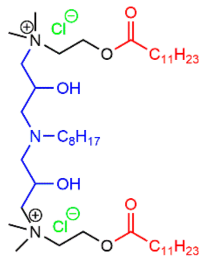
41

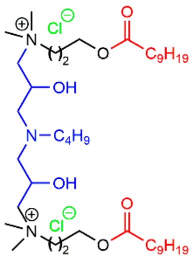
42

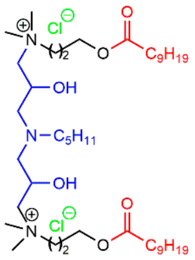
43

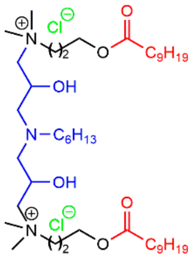
44

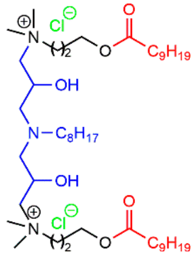
45

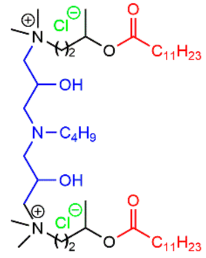
46

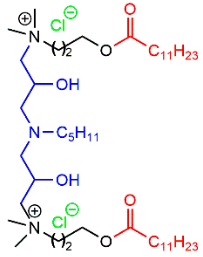
47

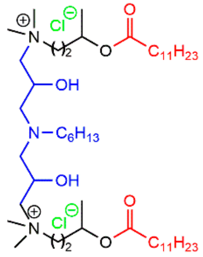
48

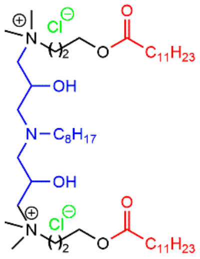
49

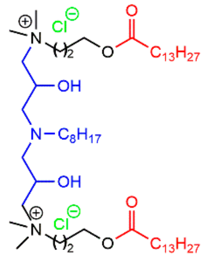
50

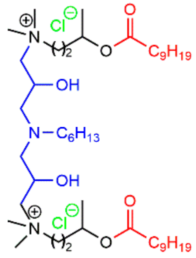
51

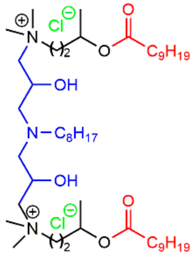
52

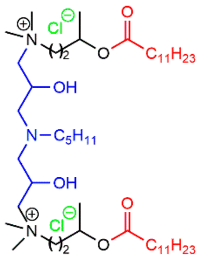
53

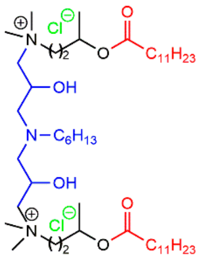
54

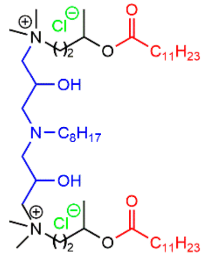
55

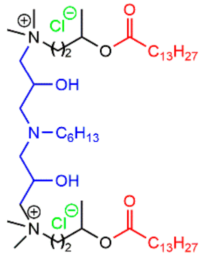
56

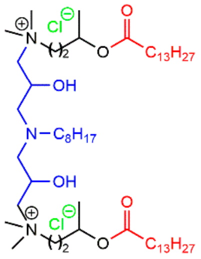
57

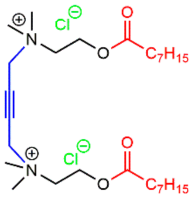
58

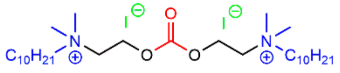
59

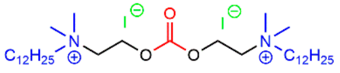
60

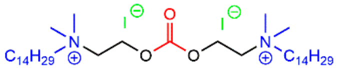
61

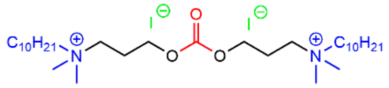
62

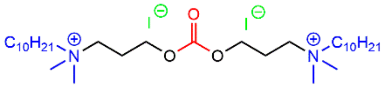
63

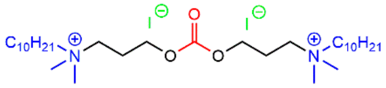
64

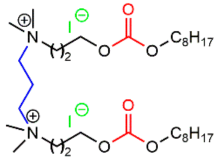
65

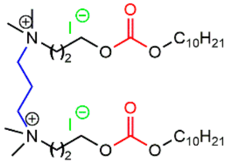
66

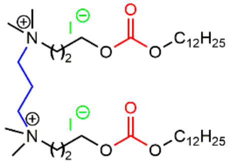
67

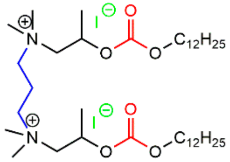
68

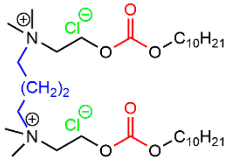
69

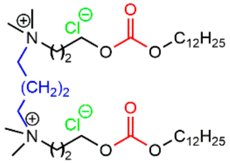
70

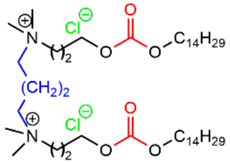
71

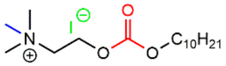
72

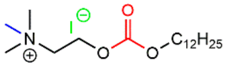
73

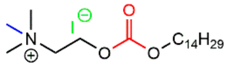
74

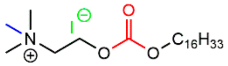
75

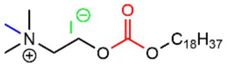
76

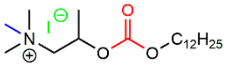
77

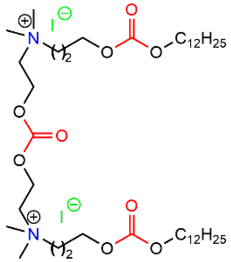
78

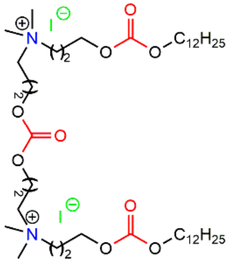
79

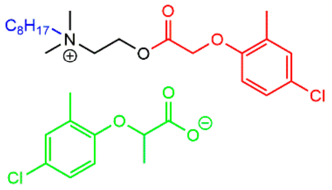
80

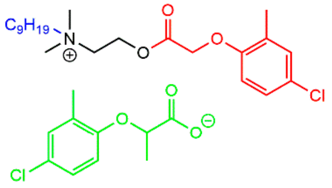
81

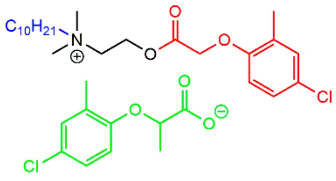
82

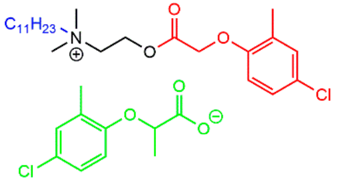
83

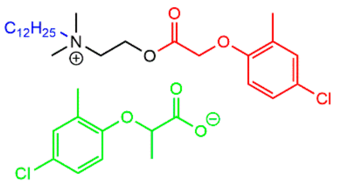
84

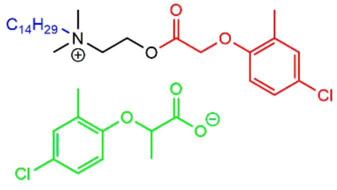
85

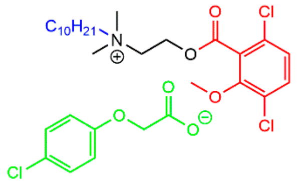
86

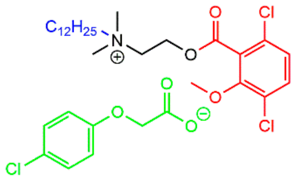
87

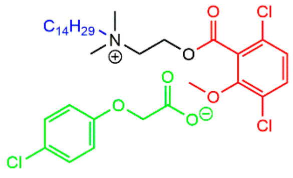
88

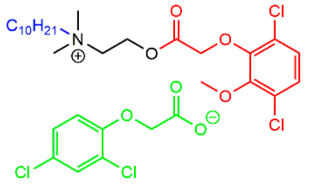
89

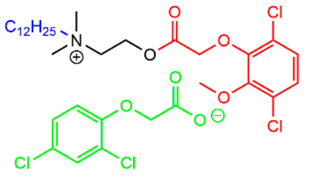
90

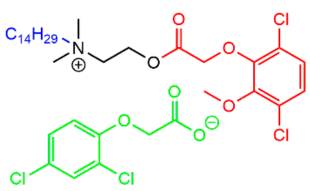

91

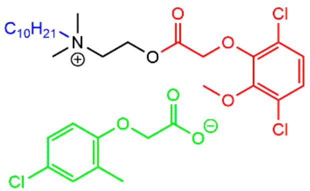

92

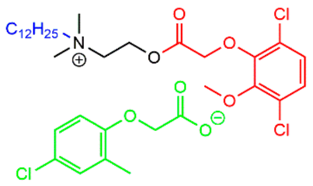

93

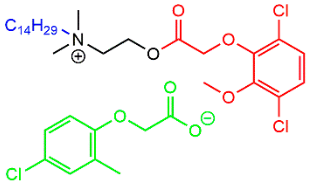

94

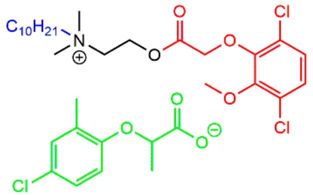

95

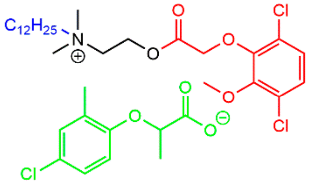

96

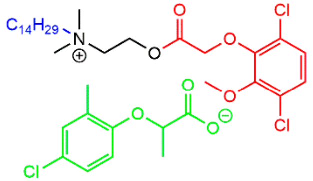

97

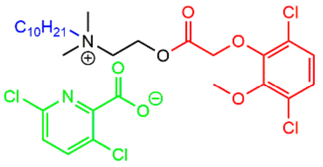

98

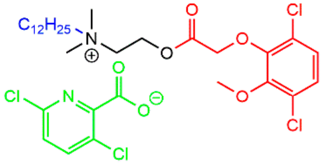

99

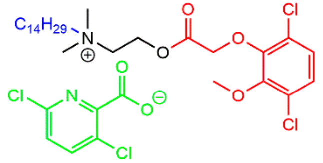

100

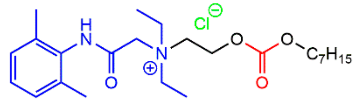

101

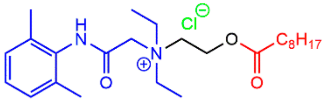

102

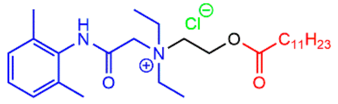

103

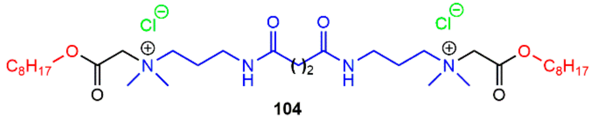

104

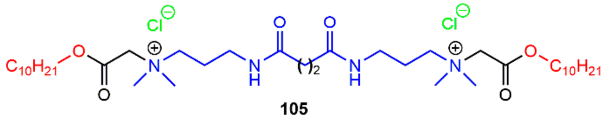

105

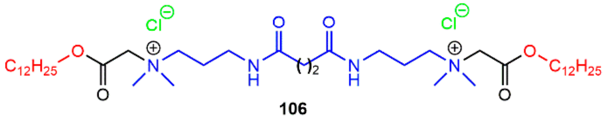

106

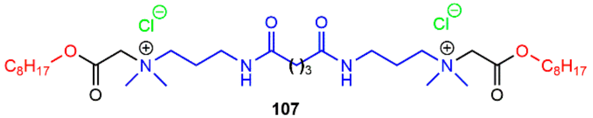

107

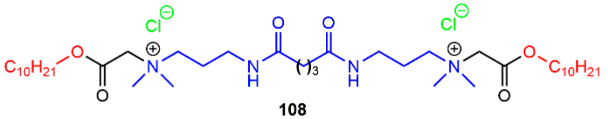

108

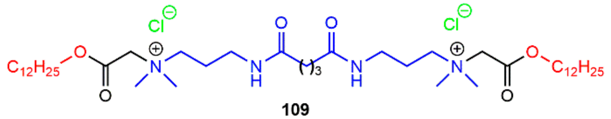

109

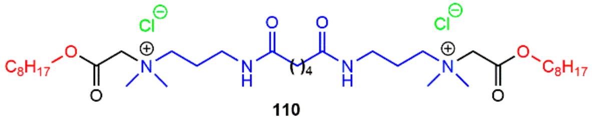

110

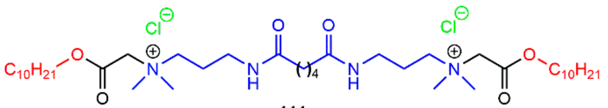

111

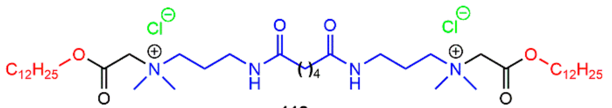

112

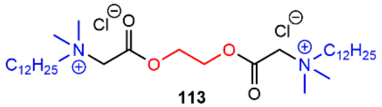

113

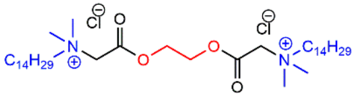
114

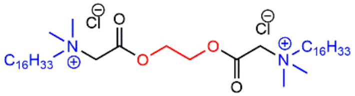
115

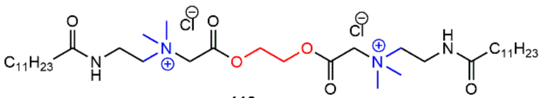
116

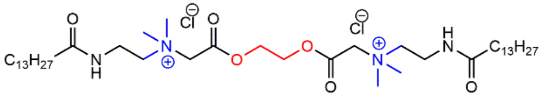
117

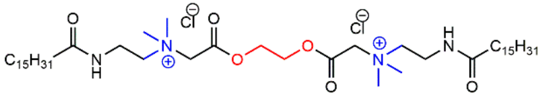
118

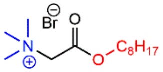
119

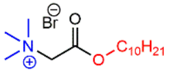
120

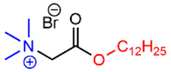
121

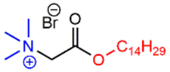
122

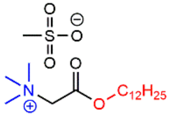
123

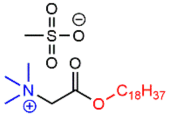
124

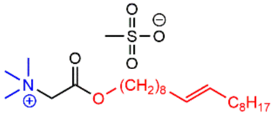
125

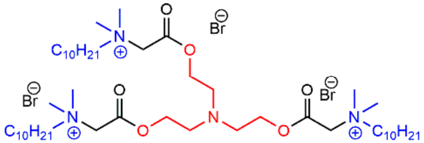
126

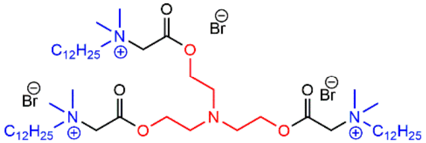
127

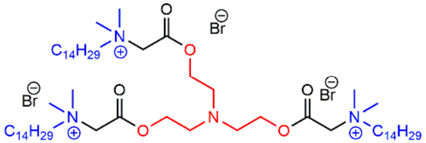
128

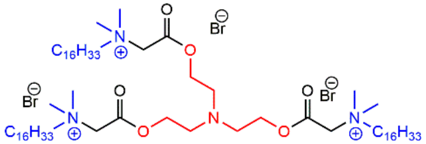
129

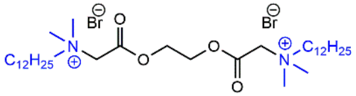
130

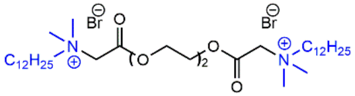
131

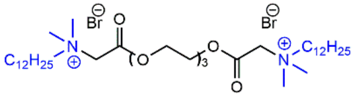
132

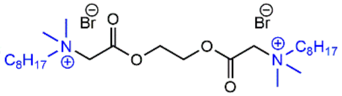
133

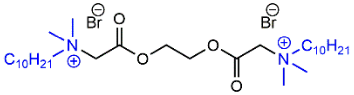
134

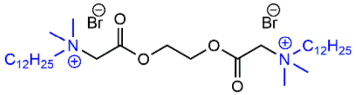
135

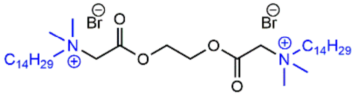
136

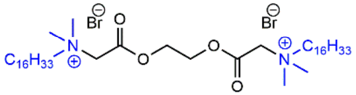
137

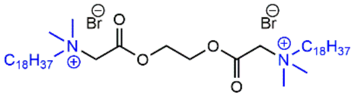
138

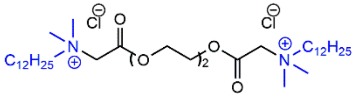
139

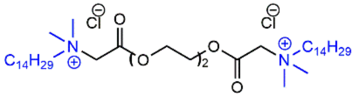
140

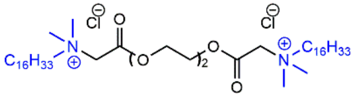
141

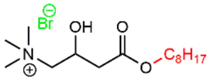
142

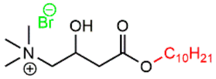
143

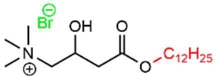
144

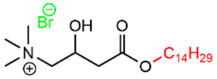
145

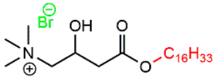
146

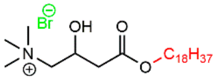
147

Table S3. Surface properties of esterquats.

No.	Cation	Anion	CMC [mmol/L] at 25°C	M [g/mol]	CMC [g/L]	Surface tension at CMC [mN·m^{-1}]	Ref.
**1**	**C2**; R_1=C_7H_{15}	Cl	3.94	452	1.78	24	[9]
**2**	**C2**; R_1=C_9H_{19}	Cl	0.197	508	0.100	25	[9]
**3**	**C2**; R_1=$C_{11}H_{23}$	Cl	0.0692	453	0.0314	28	[9]
**4**	**C4**; R_1=$C_{11}H_{23}$, R_2=C_8H_{17}	Br	0.06	525	0.0315	36	[4]
**5**	**C4**; R_1=$C_{13}H_{27}$, R_2=C_8H_{17}	Br	0.04	553	0.0221	32	[4]
**6**	**C4**; R_1=$C_{15}H_{31}$, R_2=C_8H_{17}	Br	0.03	581	0.0174	29	[4]
**7**	**C6**; n=1, R_1=C_9H_{19}, R_2=CH_2	2Br	2.06	689	1.42	---	[66]
**8**	**C6**; n=1, R_1=$C_{11}H_{23}$, R_2=CH_2	2Br	0.31	717	0.222	---	[13]
**9**	**C6**; n=1, R_1=$C_{12}H_{25}$, R_2=C_2H_4	2Cl	0.11	698	0.0768	34	[7]
**10**	**C6**; n=1, R_1=C_9H_{19}, R_2=C_4H_8	2Br	2.6	731	1.90	---	[66]
**11**	**C6**; n=1, R_1=$C_{10}H_{21}$, R_2=trans-C_2H_2	2Cl	0.79	668	0.528	35	[7]
**12**	**C6**; n=1, R_1=$C_{12}H_{25}$, R_2=trans-C_2H_2	2Cl	0.13	696	0.090	33	[7]
**13**	**C6**; n=1, R_1=$C_{14}H_{29}$, R_2=trans-C_2H_2	2Cl	0.025	724	0.0181	31	[7]
**14**	**C6**; n=1, R_1=$C_{16}H_{33}$, R_2=trans-C_2H_2	2Cl	0.0052	752	0.00391	35	[7]
**15**	**C6**; n=3, R_1=$C_{10}H_{21}$, R_2=trans-C_2H_2	2Cl	0.4	696	0.278	34	[7]
**16**	**C6**; n=5, R_1=$C_{10}H_{21}$, R_2=trans-C_2H_2	2Cl	0.27	724	0.195	39	[7]
**17**	**C6**; n=5, R_1=C_8H_{17}, R_2=trans-C_2H_2	2Cl	0.42	696	0.292	35	[7]
**18**	**C6**; n=1, R_1=$C_{12}H_{25}$, R_2=cis-C_2H_2	2Cl	0.14	696	0.0974	36	[7]
**19**	**C6**; n=1, R_1=C_9H_{19}, R_2=CH_2OH	2Cl	1.09	616	0.671	30	[6]
**20**	**C6**; n=1, R_1=$C_{11}H_{23}$, R_2=CH_2OH	2Cl	0.082	644	0.0528	32	[6]
**21**	**C6**; n=1, R_1=$C_{13}H_{27}$, R_2=CH_2OH	2Cl	0.021	672	0.0141	30	[6]

**22**	**C6**; n=2, $R_1=C_{12}H_{25}$, $R_2=CH_2OH$	2Cl	0.082	728	0.0597	32	[7]
**23**	**C6**; n=3, $R_1=C_9H_{19}$, $R_2=CH_2OH$	2Cl	0.17	644	0.109	34	[6]
**24**	**C6**; n=5, $R_1=C_7H_{15}$, $R_2=CH_2OH$	2Cl	0.79	644	0.509	34	[6]
**25**	**C7**; n=1, $R_1=C_{11}H_{23}$	Cl	0.69	322	0.222	30	[6]
**26**	**C7**; n=1, $R_1=C_9H_{19}$	Br	29.53	338	10.0	---	[66]
**27**	**C7**; n=1, $R_1=C_{11}H_{23}$	Br	7.2	366	2.64	---	[13]
**28**	**C7**; n=1, $R_1=CHCHCOOC_8H_{17}$	OH	3.2	331	1.06	32	[67]
**29**	**C7**; n=1, $R_1=CHCHCOOC_{10}H_{21}$	OH	1.1	359	0.395	32	[67]
**30**	**C7**; n=1, $R_1=CHCHCOOC_{12}H_{25}$	OH	0.36	387	0.139	31	[67]
**31**	**C11**; $R_1=C_2H_4$, $R_2=C_{10}H_{21}$	2Cl	1.514	614	0.929	39	[10]
**32**	**C11**; $R_1=C_2H_4$, $R_2=C_{12}H_{25}$	2Cl	0.238	670	0.159	35	[10]
**33**	**C11**; $R_1=C_2H_4$, $R_2=C_{14}H_{29}$	2Cl	0.211	726	0.153	36	[10]
**34**	**C11**; $R_1=C_2H_4$, $R_2=C_{16}H_{33}$	2Cl	0.024	782	0.0188	37	[10]
**35**	**C11**; $R_1=C_6H_{12}$, $R_2=C_{10}H_{21}$	2Cl	3.981	670	2.67	32	[10]
**36**	**C11**; $R_1=C_6H_{12}$, $R_2=C_{12}H_{25}$	2Cl	1.486	726	1.08	39	[10]
**37**	**C11**; $R_1=C_6H_{12}$, $R_2=C_{14}H_{29}$	2Cl	0.236	782	0.185	33	[10]
**38**	**C11**; $R_1=C_6H_{12}$, $R_2=C_{16}H_{33}$	2Cl	0.032	838	0.0268	31	[10]
**39**	**C14**; n=1, Y=H $R_1=C_{11}H_{23}$, $R_2=C_5H_{11}$	2Cl	0.624	815	0.5	30	[16]
**40**	**C14**; n=1, Y=H $R_1=C_{11}H_{23}$, $R_2=C_6H_{13}$	2Cl	0.561	829	0.465	30	[16]
**41**	**C14**; n=1, Y=H $R_1=C_{11}H_{23}$, $R_2=C_8H_{17}$	2Cl	0.393	857	0.337	30	[16]
**42**	**C14**; n=2, Y=H, $R_1=C_9H_{19}$, $R_2=C_4H_9$	2Cl	0.708	745	0.528	38	[18]
**43**	**C14**; n=2, Y=H, $R_1=C_9H_{19}$, $R_2=C_5H_{11}$	2Cl	0.282	759	0.214	36	[18]
**44**	**C14**; n=2, Y=H $R_1=C_9H_{19}$, $R_2=C_6H_{13}$	2Cl	0.0478; 0.0596	773	0.0370; 0.0461	44; 39	[16,18]

**45**	**C14**; n=2, Y=H R_1=C_9H_{19}, R_2=C_8H_{17}	2Cl	0.0282; 0.0436	829	0.0234; 0.0361	44; 37	[16,18]
**46**	**C14**; n=2, Y=H, R_1=$C_{11}H_{23}$, R_2=C_4H_9	2Cl	0.447	829	0.371	37	[18]
**47**	**C14**; n=2, Y=H R_1=$C_{11}H_{23}$, R_2=C_5H_{11}	2Cl	0.0488; 0.15	843	0.0411; 0.126	40; 35	[16,18]
**48**	**C14**; n=2, Y=H R_1=$C_{11}H_{23}$, R_2=C_6H_{13}	2Cl	0.0447	857	0.0383	38	[16,18]
**49**	**C14**; n=2, Y=H R_1=$C_{11}H_{23}$, R_2=C_8H_{17}	2Cl	0.0178; 0.0407	885	0.0158; 0.0360	37; 36	[16,18]
**50**	**C14**; n=2, Y=H R_1=$C_{13}H_{27}$, R_2=C_8H_{17}	2Cl	0.0436	941	0.0410	31	[16]
**51**	**C14**; n=2, Y=CH_3 R_1=C_9H_{19}, R_2=C_6H_{13}	2Cl	0.0842	801	0.0675	44	[16]
**52**	**C14**; n=2, Y=CH_3 R_1=C_9H_{19}, R_2=C_8H_{17}	2Cl	0.0784	829	0.0650	44	[16]
**53**	**C14**; n=2, Y=CH_3 R_1=$C_{11}H_{23}$, R_2=C_5H_{11}	2Cl	0.0628	871	0.0547	40	[16]
**54**	**C14**; n=2, Y=CH_3 R_1=$C_{11}H_{23}$, R_2=C_6H_{13}	2Cl	0.0591	885	0.0523	40	[16]
**55**	**C14**; n=2, Y=CH_3 R_1=$C_{11}H_{23}$, R_2=C_8H_{17}	2Cl	0.0541	913	0.0494	39	[16]
**56**	**C14**; n=2, Y=CH_3 R_1=$C_{13}H_{27}$, R_2=C_6H_{13}	2Cl	0.0576	941	0.0542	35	[16]
**57**	**C14**; n=2, Y=CH_3 R_1=$C_{13}H_{27}$, R_2=C_8H_{17}	2Cl	0.057	969	0.0552	35	[16]
**58**	**C15**; n=1, R_1=$C_{12}H_{25}$	2Cl	0.4	694	0.278	35	[7]
**59**	**C19**; n=1, R_1=$C_{10}H_{21}$	2I	0.747	741	0.553	37	[30]
**60**	**C19**; n=1, R_1=$C_{12}H_{25}$	2I	0.29	797	0.231	37	[30]
**61**	**C19**; n=1, R_1=$C_{14}H_{25}$	2I	0.0466	853	0.0397	39	[30]
**62**	**C19**; n=2, R_1=$C_{10}H_{21}$	2I	0.71	769	0.546	42	[30]
**63**	**C19**; n=2, R_1=$C_{12}H_{25}$	2I	0.172	825	0.142	43	[30]
**64**	**C19**; n=2, R_1=$C_{14}H_{25}$	2I	0.0431	881	0.0380	43	[30]
**65**	**C20**; n=2, Y=H, R_1=C_8H_{27} R_2=CH_2	2I	0.948	871	0.826	32	[31]

**66**	**C20**; n=2, Y=H, R_1=$C_{10}H_{31}$ R_2=CH_2	2I	0.146	899	0.131	30	[31]
**67**	**C20**; n=2, Y=H, R_1=$C_{12}H_{35}$ R_2=CH_2	2I	0.0578	927	0.0536	31	[31]
**68**	**C20**; n=1, Y=CH_3, R_1=$C_{12}H_{35}$ R_2=CH_2	2I	0.0254	947	0.0241	33	[31]
**69**	**C20**; n=1, Y=H, R_1=$C_{10}H_{21}$, R_2=C_2	2Cl	4.63	599	2.77	39	[29]
**70**	**C20**; n=1, Y=H, R_1=$C_{12}H_{25}$, R_2=C_2	2Cl	0.69	655	0.452	39	[29]
**71**	**C20**; n=1, Y=H, R_1=$C_{14}H_{29}$, R_2=C_2	2Cl	0.092	711	0.0654	37	[29]
**72**	**C21**; n=1, Y=H, R_1=$C_{10}H_{21}$	I	11.82	415	4.91	30	[29]
**73**	**C21**; n=1, Y=H, R_1=$C_{12}H_{25}$	I	3.27; 0.431	443	1.45; 0.191	29	[29,31]
**74**	**C21**; n=1, Y=H, R_1=$C_{14}H_{29}$	I	1.78	471	0.839	29	[29]
**75**	**C21**; n=1, Y=H, R_1=$C_{16}H_{33}$	I	0.37	499	0.185	30	[29]
**76**	**C21**; n=1, Y=H, R_1=$C_{18}H_{37}$	I	0.31	527	0.163	30	[29]
**77**	**C21**; n=1, Y=CH_3, R_1=$C_{12}H_{25}$	I	1.29	457	0.590	35	[31]
**78**	**C22**; n=2, m=1, R_1=$C_{12}H_{35}$	2I	0.0401	1001	0.0401	34	[31]
**79**	**C22**; n=2, m=2, R_1=$C_{12}H_{35}$	2I	0.0571	1029	0.0588	37	[31]
**80**	**C25'**; R_2=C_8H_{17}	MCPP	0.98	599	0.587	33	[68]
**81**	**C25'**; R_2=C_9H_{19}	MCPP	0.82	613	0.502	33	[68]
**82**	**C25'**; R_2=$C_{10}H_{21}$	MCPP	0.53	627	0.332	31	[68]
**83**	**C25'**; R_2=$C_{11}H_{23}$	MCPP	0.56	627	0.351	30	[68]
**84**	**C25'**; R_2=$C_{12}H_{25}$	MCPP	0.45	655	0.295	27	[68]
**85**	**C25'**; R_2=$C_{14}H_{29}$	MCPP	0.41	683	0.280	27	[68]
**86**	**C25''**; R_2=$C_{10}H_{21}$	4-CPA	1.42	619	0.879	25	[20]
**87**	**C25''**; R_2=$C_{12}H_{25}$	4-CPA	4.64	647	3.00	26	[20]
**88**	**C25''**; R_2=$C_{14}H_{29}$	4-CPA	2.12	675	1.43	31	[20]

**89**	**C25''**; R_2=$C_{10}H_{21}$	2,4-D	2.86	653	1.87	27	[20]
**90**	**C25''**; R_2=$C_{12}H_{25}$	2,4-D	6.83	682	4.65	28	[20]
**91**	**C25''**; R_2=$C_{14}H_{29}$	2,4-D	7.1	710	5.04	32	[20]
**92**	**C25''**; R_2=$C_{10}H_{21}$	MCPA	1.19	633	0.753	24	[20]
**93**	**C25''**; R_2=$C_{12}H_{25}$	MCPA	1.53	661	1.01	29	[20]
**94**	**C25''**; R_2=$C_{14}H_{29}$	MCPA	2.24	689	1.54	33	[20]
**95**	**C25''**; R_2=$C_{10}H_{21}$	MCPP	0.29	647	0.188	34	[20]
**96**	**C25''**; R_2=$C_{12}H_{25}$	MCPP	0.71	675	0.479	30	[20]
**97**	**C25''**; R_2=$C_{14}H_{29}$	MCPP	0.68	703	0.478	36	[20]
**98**	**C25''**; R_2=$C_{10}H_{21}$	clopyralid	0.52	624	0.325	32	[20]
**99**	**C25''**; R_2=$C_{12}H_{25}$	clopyralid	0.14	652	0.0913	32	[20]
**100**	**C25''**; R_2=$C_{14}H_{29}$	clopyralid	0.06	681	0.0408	35	[20]
**101**	**C26**, n=1, R_1=C_7H_{15}	Cl	2.51	457	1.15	33	[33]
**102**	**C27**, R_1=C_8H_{17}	Cl	1.27	455	0.578	22	[33]
**103**	**C27**, R_1=$C_{11}H_{23}$	Cl	1.27	497	0.631	24	[33]
**104**	**B4**; n=1, m=2, R_1=C_8H_{17}	2Cl	2.26	700	1.58	---	[42]
**105**	**B4**; n=1, m=2, R_1=$C_{10}H_{21}$	2Cl	2.11	756	1.59	---	[42]
**106**	**B4**; n=1, m=2, R_1=$C_{12}H_{25}$	2Cl	1.55	812	1.26	---	[42]
**107**	**B4**; n=1, m=3, R_1=C_8H_{17}	2Cl	2.56	714	1.83	---	[42]
**108**	**B4**; n=1, m=3, R_1=$C_{10}H_{21}$	2Cl	2.27	770	1.75	---	[42]
**109**	**B4**; n=1, m=3, R_1=$C_{12}H_{25}$	2Cl	1.37	826	1.13	---	[42]
**110**	**B4**; n=1, m=4, R_1=C_8H_{17}	2Cl	2.62	728	1.91	---	[42]
**111**	**B4**; n=1, m=4, R_1=$C_{10}H_{21}$	2Cl	2.48	784	1.94	---	[42]

**112**	**B4**; n=1, m=4, $R_1=C_{12}H_{25}$	2Cl	1.5	840	1.26	---	[42]
**113**	**B5**; n=1, $R_1=C_{12}H_{25}$	2Cl	0.0016	642	0.00103	---	[69]
**114**	**B5**; n=1, $R_1=C_{14}H_{29}$	2Cl	0.0014	698	0.00098	---	[69]
**115**	**B5**; n=1, $R_1=C_{16}H_{33}$	2Cl	0.0013	754	0.00098	---	[69]
**116**	**B5**; n=1, $R_1=C_{12}H_{25}CONHC_2H_4$	2Cl	2.6	784	2.04	27	[70]
**117**	**B5**; n=1, $R_1=C_{14}H_{29}CONHC_2H_4$	2Cl	1.7	840	1.43	26	[70]
**118**	**B5**; n=1, $R_1=C_{16}H_{33}CONHC_2H_4$	2Cl	1.1	896	0.986	25	[70]
**119**	**B6**; n=1; $R_1=C_8H_{17}$	Br	80.38	230	18.5	38	[71]
**120**	**B6**; n=1; $R_1=C_{10}H_{21}$	Br	16.94	258	4.38	30	[71]
**121**	**B6**; n=1; $R_1=C_{12}H_{25}$	Br	4.9; 5	286	1.40; 1.43	27	[14,71]
**122**	**B6**; n=1; $R_1=C_{14}H_{29}$	Br	1.36	314	0.428	32	[71]
**123**	**B6**; n=1; $R_1=C_{12}H_{25}$	CH_3SO_3	1.2	382	0.458	32	[55]
**124**	**B6**; n=1; $R_1=C_{18}H_{37}$	CH_3SO_3	0.794	466	0.370	37	[55]
**125**	**B6**; n=1; $R_1=(CH_2)_8CHCHC_8H_{17}$	CH_3SO_3	0.1	464	0.0464	37	[55]
**126**	**B9**; n=1; $R_1=C_{10}H_{21}$	3Br	1.73	1068	1.85	38	[40]
**127**	**B9**; n=1; $R_1=C_{12}H_{25}$	3Br	0.124	1152	0.143	42	[40]
**128**	**B9**; n=1; $R_1=C_{14}H_{29}$	3Br	0.0508	1236	0.0628	38	[40]
**129**	**B9**; n=1; $R_1=C_{16}H_{32}$	3Br	0.0062	1320	0.00818	39	[40]
**130**	**B19**; m=1; $R_1=C_{12}H_{25}$	2Br	0.55	731	0.402	29	[48]
**131**	**B19**; m=2; $R_1=C_{12}H_{25}$	2Br	0.61	776	0.473	31	[48]
**132**	**B19**; m=3; $R_1=C_{12}H_{25}$	2Br	0.67	821	0.550	31	[48]
**133**	**B19**; m=1; $R_1=C_8H_{17}$	2Br	11.03	619	6.82	40	[49]
**134**	**B19**; m=1; $R_1=C_{10}H_{21}$	2Br	2.33	675	1.57	39	[49]

**135**	**B19**; m=1; $R_1=C_{12}H_{25}$	2Br	1.06	731	0.774	36	[49]
**136**	**B19**; m=1; $R_1=C_{14}H_{29}$	2Br	0.15	787	0.118	34	[49]
**137**	**B19**; m=1; $R_1=C_{16}H_{33}$	2Br	0.07	843	0.0590	36	[49]
**138**	**B19**; m=1; $R_1=C_{18}H_{37}$	2Br	0.02	899	0.0180	38	[49]
**139**	**B19**; m=2; $R_1=C_{12}H_{25}$	2Cl	0.075	685	0.0514	54	[72]
**140**	**B19**; m=2; $R_1=C_{14}H_{29}$	2Cl	0.025	741	0.0185	47	[72]
**141**	**B19**; m=2; $R_1=C_{16}H_{33}$	2Cl	0.011	797	0.00877	39	[72]
**142**	**B31**; $R_1=C_8H_{17}$	Br	48.59	354	17.2	38	[71]
**143**	**B31**; $R_1=C_{10}H_{21}$	Br	13.12	382	5.02	38	[71]
**144**	**B31**; $R_1=C_{12}H_{25}$	Br	2.51	410	1.03	36	[71]
**145**	**B31**; $R_1=C_{14}H_{29}$	Br	0.76	438	0.333	39	[71]
**146**	**B31**; $R_1=C_{16}H_{33}$	Br	0.42	466	0.196	39	[71]
**147**	**B31**; $R_1=C_{18}H_{37}$	Br	0.16	494	0.0791	51	[71]

Acknowledgments: We want to thank Aleksandra Nowacka for help with drawing structures of compounds in chapter "4. Structures".

6. Bibliography

(1) Chung, Y.; Park, S. Reversed Phase High Performance Liquid Chromatographic Separation of Esterquats with Indirect Spectrophotometric Detection. *Microchemical Journal* **1998**, *60* (1), 42–50. https://doi.org/10.1006/mchj.1998.1635.

(2) Steiger, P.; Friedli, F. E. A Study on the Long-Term Stability and Removal of Quats on Treated Fabric. *J Surfact Deterg* **2002**, *5* (3), 207–210. https://doi.org/10.1007/s11743-002-0219-8.

(3) Gunjan; Tyagi, V. K. Synthesis of Rice Bran Fatty Acids (RBFAs) Based Cationic Surfactants and Evaluation of Their Performance Properties in Combination with Nonionic Surfactant. *Tenside Surfactants Detergents* **2014**, *51* (6), 497–505. https://doi.org/10.3139/113.110334.

(4) Narayanan, V. L.; Umapathy, M. J. Synthesis, Characterization and Antimicrobial Evaluation of Three New Cationic Surfactants. *Tenside Surfactants Detergents* **2015**, *52* (2), 170–178. https://doi.org/10.3139/113.110363.

(5) Umapathy, M. J.; Narayanan, V. L.; Magesan, P.; Chiranjeevi, P.; Jemima, S. W. Synthesis and Characterization of Biodegradable Cationic Esterquat Surfactants and the Evaluation of Its Physico-Chemical Properties. *Tenside Surfactants Detergents* **2016**, *53* (3), 249–258. https://doi.org/10.3139/113.110430.

(6) Tatsumi, T.; Zhang, W.; Kida, T.; Nakatsuji, Y.; Ono, D.; Takeda, T.; Ikeda, I. Novel Hydrolyzable and Biodegradable Cationic Gemini Surfactants: 1,3-Bis[(Acyloxyalkyl)-Dimethylammonio]-2-Hydroxypropane Dichloride. *J Surfact Deterg* **2000**, *3* (2), 167–172. https://doi.org/10.1007/s11743-000-0121-4.

(7) Tatsumi, T.; Zhang, W.; Kida, T.; Nakatsuji, Y.; Ono, D.; Takeda, T.; Ikeda, I. Novel Hydrolyzable and Biodegradable Cationic Gemini Surfactants: Bis(Ester-Ammonium) Dichloride Having a Butenylene or a Butynylene Spacer. *J Surfact Deterg* **2001**, *4* (3), 279–285. https://doi.org/10.1007/s11743-001-0180-6.

(8) Xu, Q.; Wang, L.; Xing, F. Synthesis and Properties of Dissymmetric Gemini Surfactants. *J Surfact Deterg* **2011**, *14* (1), 85–90. https://doi.org/10.1007/s11743-010-1207-6.

(9) Han, B.; Geng, T.; Jiang, Y.; Ju, H. Synthesis and Properties of Di-Chain Esterquat Surfactants. *J Surfact Deterg* **2015**, *18* (1), 91–95. https://doi.org/10.1007/s11743-014-1597-y.

(10) Gao, X.; Wang, Y.; Zhao, X.; Wei, W.; Chang, H. Equilibrium and Dynamic Surface Tension Properties of Gemini Quaternary Ammonium Salt Surfactants with Ester Groups. *Colloids and Surfaces A: Physicochemical and Engineering Aspects* **2016**, *509*, 130–139. https://doi.org/10.1016/j.colsurfa.2016.08.089.

(11) HERA. *Esterquats Environmental Risk Assessment Report*; 2008; p 42.

(12) Fahri, F.; Bacha, K.; Chiki, F. F.; Mbakidi, J.-P.; Panda, S.; Bouquillon, S.; Fourmentin, S. Air Pollution: New Bio-Based Ionic Liquids Absorb Both Hydrophobic and Hydrophilic Volatile Organic Compounds with High Efficiency. *Environ Chem Lett* **2020**, *18* (4), 1403–1411. https://doi.org/10.1007/s10311-020-01007-8.

(13) Tehrani-Bagha, A. R.; Oskarsson, H.; van Ginkel, C. G.; Holmberg, K. Cationic Ester-Containing Gemini Surfactants: Chemical Hydrolysis and Biodegradation. *Journal of Colloid and Interface Science* **2007**, *312* (2), 444–452. https://doi.org/10.1016/j.jcis.2007.03.044.

(14) Para, G.; Łuczyński, J.; Palus, J.; Jarek, E.; Wilk, K. A.; Warszyński, P. Hydrolysis Driven Surface Activity of Esterquat Surfactants. *Journal of Colloid and Interface Science* **2016**, *465*, 174–182. https://doi.org/10.1016/j.jcis.2015.11.056.

(15) Bregadze, V. I.; Ermanson, L. V.; Godovikov, N. N. Synthesis of Choline Esters of Carboranecarboxylic Acids. *Russ Chem Bull* **1976**, *25* (11), 2425–2427. https://doi.org/10.1007/BF00973414.
(16) Węgrzyńska, J.; Chlebicki, J.; Maliszewska, I. Preparation, Surface-Active Properties and Antimicrobial Activities of Bis(Ester Quaternary Ammonium) Salts. *J Surfact Deterg* **2007**, *10* (2), 109–116. https://doi.org/10.1007/s11743-007-1020-z.
(17) Pernak, J.; Syguda, A.; Janiszewska, D.; Materna, K.; Praczyk, T. Ionic Liquids with Herbicidal Anions. *Tetrahedron* **2011**, *67* (26), 4838–4844. https://doi.org/10.1016/j.tet.2011.05.016.
(18) Migahed, M. A.; Negm, N. A.; Shaban, M. M.; Ali, T. A.; Fadda, A. A. Synthesis, Characterization, Surface and Biological Activity of Diquaternary Cationic Surfactants Containing Ester Linkage. *J Surfact Deterg* **2016**, *19* (1), 119–128. https://doi.org/10.1007/s11743-015-1749-8.
(19) Syguda, A.; Gielnik, A.; Borkowski, A.; Woźniak-Karczewska, M.; Parus, A.; Piechalak, A.; Olejnik, A.; Marecik, R.; Ławniczak, Ł.; Chrzanowski, Ł. Esterquat Herbicidal Ionic Liquids (HILs) with Two Different Herbicides: Evaluation of Activity and Phytotoxicity. *New J. Chem.* **2018**, *42* (12), 9819–9827. https://doi.org/10.1039/C8NJ01239C.
(20) Syguda, A.; Wojcieszak, M.; Materna, K.; Woźniak-Karczewska, M.; Parus, A.; Ławniczak, Ł.; Chrzanowski, Ł. Double-Action Herbicidal Ionic Liquids Based on Dicamba Esterquats with 4-CPA, 2,4-D, MCPA, MCPP, and Clopyralid Anions. *ACS Sustainable Chem. Eng.* **2020**, *8* (38), 14584–14594. https://doi.org/10.1021/acssuschemeng.0c05603.
(21) Liu, D.; Yang, X.; Liu, P.; Mao, T.; Shang, X.; Wang, L. Synthesis and Characterization of Gemini Ester Surfactant and Its Application in Efficient Fabric Softening. *Journal of Molecular Liquids* **2020**, *299*, 112236. https://doi.org/10.1016/j.molliq.2019.112236.
(22) Lozano, P.; Daz, M.; De Diego, T.; Iborra, J. L. Ester Synthesis from Trimethylammonium Alcohols in Dry Organic Media Catalyzed by immobilizedCandida Antarctica Lipase B. *Biotechnol. Bioeng.* **2003**, *82* (3), 352–358. https://doi.org/10.1002/bit.10580.
(23) Mouterde, L. M. M.; Peru, A. A. M.; Mention, M. M.; Brunissen, F.; Allais, F. Sustainable Straightforward Synthesis and Evaluation of the Antioxidant and Antimicrobial Activity of Sinapine and Analogues. *J. Agric. Food Chem.* **2020**, *68* (26), 6998–7004. https://doi.org/10.1021/acs.jafc.0c02183.
(24) Tammelin, L.-E.; Gmelin, R.; Jensen, R. B.; Stenhagen, E.; Thorell, B. Syntheses of Choline Esters of Monobasic Carbonic Acids. *Acta Chem. Scand.* **1956**, *10*, 145–146. https://doi.org/10.3891/acta.chem.scand.10-0145.
(25) Topuzyan, V. O.; Gerasimyan, D. A.; Bagdasaryan, A. L.; Mndzhoyan, O. L. Synthesis and Biological Properties of Choline Esters of Amino Acids. *Pharm Chem J* **1983**, *17* (2), 87–91. https://doi.org/10.1007/BF00765209.
(26) Hellberg, P.-E. Ortho Ester-Based Cleavable Cationic Surfactants. *J Surfact Deterg* **2002**, *5* (3), 217–227. https://doi.org/10.1007/s11743-002-0221-1.
(27) Banno, T.; Toshima, K.; Kawada, K.; Matsumura, S. Synthesis and Properties of Biodegradable and Chemically Recyclable Cationic Surfactants Containing Carbonate Linkages. *J. Oleo Sci.* **2007**, *56* (9), 493–499. https://doi.org/10.5650/jos.56.493.
(28) Topuzyan, V. O.; Karapetyan, I. R.; Alebyan, G. P. Synthesis and Anticholinesterase Properties of Choline Esters of α-Amino Acids. *Pharm Chem J* **2014**, *48* (3), 163–165. https://doi.org/10.1007/s11094-014-1070-2.
(29) Gilbert, E. A.; Guastavino, J. F.; Murguía, M. C. Synthesis and Surface-active Properties of Novel Cleavable Gemini Surfactants. *J Surfact Deterg* **2021**, jsde.12547. https://doi.org/10.1002/jsde.12547.
(30) Banno, T.; Toshima, K.; Kawada, K.; Matsumura, S. Synthesis and Properties of Gemini-Type Cationic Surfactants Containing Carbonate Linkages in the Linker Moiety Directed Toward Green and Sustainable Chemistry. *J Surfact Deterg* **2009**, *12* (3), 249–259. https://doi.org/10.1007/s11743-009-1119-5.

(31) Banno, T.; Kawada, K.; Matsumura, S. Creation of Novel Green and Sustainable Gemini-Type Cationics Containing Carbonate Linkages. *J Surfact Deterg* **2010**, *13* (4), 387–398. https://doi.org/10.1007/s11743-010-1224-5.
(32) Stachowiak, W.; Wysocki, M.; Niemczak, M. “Bitter” Results: Toward Sustainable Synthesis of the Most Bitter Substances, Denatonium Saccharinate and Denatonium Benzoate, Starting from a Popular Anesthetic, Lidocaine. *J. Chem. Educ.* **2022**, *99* (4), 1604–1611. https://doi.org/10.1021/acs.jchemed.1c00995.
(33) Tang, L.; Yang, J.; Yin, Q.; Yang, L.; Gong, D.; Qin, F.; Liu, J.; Fan, Q.; Li, J.; Zhao, W.; Zhang, W.; Wang, J.; Zhu, T.; Zhang, W.; Liu, J. Janus Particles Self-Assembled from a Small Organic Atypical Asymmetric Gemini Surfactant. *Chem. Commun.* **2017**, *53* (62), 8675–8678. https://doi.org/10.1039/C7CC03973E.
(34) Lindstedt, M.; Allenmark, S.; Thompson, R. A.; Edebo, L. Antimicrobial Activity of Betaine Esters, Quaternary Ammonium Amphiphiles Which Spontaneously Hydrolyze into Nontoxic Components. *Antimicrob Agents Chemother* **1990**, *34* (10), 1949–1954. https://doi.org/10.1128/AAC.34.10.1949.
(35) Holden, D. A. Synthesis and Spreading Behaviour of Some Reactive Derivatives of Long-Chain Alcohols and Carboxylic Acids. *Can. J. Chem.* **1984**, *62* (3), 574–579. https://doi.org/10.1139/v84-096.
(36) Nederberg, F.; Bowden, T.; Hilborn, J. Synthesis, Characterization, and Properties of Phosphoryl Choline Functionalized Poly ε-Caprolactone and Charged Phospholipid Analogues. *Macromolecules* **2004**, *37* (3), 954–965. https://doi.org/10.1021/ma035433b.
(37) Lundberg, D.; Holmberg, K. Nuclear Magnetic Resonance Studies on Hydrolysis Kinetics and Micellar Growth in Solutions of Surface-Active Betaine Esters. *J Surfact Deterg* **2004**, *7* (3), 239–246. https://doi.org/10.1007/s11743-004-0307-9.
(38) Fatma, N.; Ansari, W. H.; Panda, M.; ud-Din, K. Mixed Micellization Behavior of Gemini (Cationic Ester-Bonded) Surfactants with Conventional (Cationic, Anionic and Nonionic) Surfactants in Aqueous Medium. *Zeitschrift für Physikalische Chemie* **2013**, *227* (1). https://doi.org/10.1524/zpch.2013.0288.
(39) Zhou, X.; Chen, Y.; Han, J.; Wu, X.; Wang, G.; Jiang, D. Betaine Ester-Shell Functionalized Hyperbranched Polymers for Potential Antimicrobial Usage: Guest Loading Capability, pH Controlled Release and Adjustable Compatibility. *Polymer* **2014**, *55* (24), 6261–6270. https://doi.org/10.1016/j.polymer.2014.10.020.
(40) Liu, X.-G.; Xing, X.-J.; Gao, Z.-N. Synthesis and Physicochemical Properties of Star-like Cationic Trimeric Surfactants. *Colloids and Surfaces A: Physicochemical and Engineering Aspects* **2014**, *457*, 374–381. https://doi.org/10.1016/j.colsurfa.2014.06.019.
(41) Thompson, R. A.; Allenmark, S.; Larsen, S.; Teuber, L.; Lucanska, B.; Krätsmar-Smogrovic, J.; Valent, A.; Alminger, T.; Erickson, M.; Grundevik, I.; Hagin, I.; Hoffman, K.-J.; Johansson, S.; Larsson, S.; Löfberg, I.; Ohlson, K.; Persson, B.; Skånberg, I.; Tekenbergs-Hjelte, L. Effects of Molecular Association on the Rates of Hydrolysis of Long-Chain Alkyl Betainates (Alkoxycarbonyl-N,N,N-Trialkylmethanaminium Halides). *Acta Chem. Scand.* **1989**, *43*, 690–693. https://doi.org/10.3891/acta.chem.scand.43-0690.
(42) Wu, J.; Gao, H.; Shi, D.; Yang, Y.; Zhang, Y.; Zhu, W. Cationic Gemini Surfactants Containing Both Amide and Ester Groups: Synthesis, Surface Properties and Antibacterial Activity. *Journal of Molecular Liquids* **2020**, *299*, 112248. https://doi.org/10.1016/j.molliq.2019.112248.
(43) Zhang, Z.; Cheng, G.; Carr, L. R.; Vaisocherová, H.; Chen, S.; Jiang, S. The Hydrolysis of Cationic Polycarboxybetaine Esters to Zwitterionic Polycarboxybetaines with Controlled Properties. *Biomaterials* **2008**, *29* (36), 4719–4725. https://doi.org/10.1016/j.biomaterials.2008.08.030.

(44) Messadi, A.; Mohamadou, A.; Boudesocque, S.; Dupont, L.; Fricoteaux, P.; Nguyen-Van-Nhien, A.; Courty, M. Syntheses and Characterisation of Hydrophobic Ionic Liquids Containing Trialkyl(2-Ethoxy-2-Oxoethyl)Ammonium or N-(1-Methylpyrrolidyl-2-Ethoxy-2-Oxoethyl)Ammonium Cations. *Journal of Molecular Liquids* **2013**, *184*, 68–72. https://doi.org/10.1016/j.molliq.2013.04.023.

(45) Kim, J.-H.; Woo, H.-S.; Jin, S.-J.; Lee, J. S.; Kim, W.; Ryu, K.; Kim, D.-W. Lithium–Oxygen Batteries with Ester-Functionalized Ionic Liquid-Based Electrolytes. *RSC Adv.* **2015**, *5* (97), 80014–80021. https://doi.org/10.1039/C5RA13682B.

(46) Allen, R. A.; Jennings, M. C.; Mitchell, M. A.; Al-Khalifa, S. E.; Wuest, W. M.; Minbiole, K. P. C. Ester- and Amide-Containing multiQACs: Exploring Multicationic Soft Antimicrobial Agents. *Bioorganic & Medicinal Chemistry Letters* **2017**, *27* (10), 2107–2112. https://doi.org/10.1016/j.bmcl.2017.03.077.

(47) Yasa, S. R.; Poornachandra, Y.; Kumar, C. G.; Penumarthy, V. Synthesis, Characterization, Antimicrobial and Anti-Biofilm Activity of a New Class of 11-Bromoundecanoic Acid-Based Betaines. *Med Chem Res* **2017**, *26* (10), 2592–2601. https://doi.org/10.1007/s00044-017-1958-y.

(48) Bhadani, A.; Endo, T.; Sakai, K.; Sakai, H.; Abe, M. Synthesis and Dilute Aqueous Solution Properties of Ester Functionalized Cationic Gemini Surfactants Having Different Ethylene Oxide Units as Spacer. *Colloid Polym Sci* **2014**, *292* (7), 1685–1692. https://doi.org/10.1007/s00396-014-3233-9.

(49) Wen, Y.; Ge, X.; Gao, W.; Wei, W.; Qiao, Y.; Chang, H. Synthesis and Aggregation Properties of Ethylene Glycol Ester-Based Cationic Gemini Surfactants. *Colloid and Interface Science Communications* **2020**, *37*, 100274. https://doi.org/10.1016/j.colcom.2020.100274.

(50) Garcia, M. T.; Ribosa, I.; Kowalczyk, I.; Pakiet, M.; Brycki, B. Biodegradability and Aquatic Toxicity of New Cleavable Betainate Cationic Oligomeric Surfactants. *Journal of Hazardous Materials* **2019**, *371*, 108–114. https://doi.org/10.1016/j.jhazmat.2019.03.005.

(51) Yasa, S. R.; Kaki, S. S.; Poornachandra, Y.; Kumar, C. G.; Penumarthy, V. Synthesis, Characterization, Antimicrobial and Biofilm Inhibitory Studies of New Esterquats. *Bioorganic & Medicinal Chemistry Letters* **2016**, *26* (8), 1978–1982. https://doi.org/10.1016/j.bmcl.2016.03.002.

(52) Granö, H.; Yli-Kauhaluoma, J.; Suortti, T.; Käki, J.; Nurmi, K. Preparation of Starch Betainate: A Novel Cationic Starch Derivative. *Carbohydrate Polymers* **2000**, *41* (3), 277–283. https://doi.org/10.1016/S0144-8617(99)00146-0.

(53) Sievänen, K.; Kavakka, J.; Hirsilä, P.; Vainio, P.; Karisalmi, K.; Fiskari, J.; Kilpeläinen, I. Cationic Cellulose Betainate for Wastewater Treatment. *Cellulose* **2015**, *22* (3), 1861–1872. https://doi.org/10.1007/s10570-015-0578-2.

(54) Vassel, B.; Skelly, W. G. N-Chlorobetainyl Chloride: Ammonium Chloride, [(Chloroformyl)Methyl]Trimethyl-. In *Organic Syntheses*; John Wiley & Sons, Inc., Ed.; John Wiley & Sons, Inc.: Hoboken, NJ, USA, 2003; pp 28–28. https://doi.org/10.1002/0471264180.os035.09.

(55) Goursaud, F.; Berchel, M.; Guilbot, J.; Legros, N.; Lemiègre, L.; Marcilloux, J.; Plusquellec, D.; Benvegnu, T. Glycine Betaine as a Renewable Raw Material to “Greener” New Cationic Surfactants. *Green Chem.* **2008**, *10* (3), 310. https://doi.org/10.1039/b713429k.

(56) De Gaetano, Y.; Mohamadou, A.; Boudesocque, S.; Hubert, J.; Plantier-Royon, R.; Dupont, L. Ionic Liquids Derived from Esters of Glycine Betaine: Synthesis and Characterization. *Journal of Molecular Liquids* **2015**, *207*, 60–66. https://doi.org/10.1016/j.molliq.2015.03.016.

(57) Pérusse, D.; Guégan, J. P.; Rolland, H.; Guilbot, J.; Benvegnu, T. Efficient Solvent-Free Cationization of Alkylpolyglycoside Based Surfactant Compositions Using Natural Glycine Betaine. *Green Chem.* **2016**, *18* (6), 1664–1673. https://doi.org/10.1039/C5GC02214B.

(58) Journoux-Lapp, C.; De Oliveira Vigier, K.; Bachmann, C.; Marinkovic, S.; Estrine, B.; Frapper, G.; Jérôme, F. Elucidation of the Role of Betaine Hydrochloride in Glycerol Esterification: Towards Bio-Based Ionic Building Blocks. *Green Chem.* **2017**, *19* (23), 5647–5652. https://doi.org/10.1039/C7GC02767B.

(59) Sharma, M.; Aguado, R.; Murtinho, D.; Valente, A. J. M.; Ferreira, P. J. T. Novel Approach on the Synthesis of Starch Betainate by Transesterification. *International Journal of Biological Macromolecules* **2021**, *182*, 1681–1689. https://doi.org/10.1016/j.ijbiomac.2021.05.175.
(60) Duan, P.; Xu, Q.; Zhang, X.; Chen, J.; Zheng, W.; Li, L.; Yang, J.; Fu, F.; Diao, H.; Liu, X. Naturally Occurring Betaine Grafted on Cotton Fabric for Achieving Antibacterial and Anti-Protein Adsorption Functions. *Cellulose* **2020**, *27* (11), 6603–6615. https://doi.org/10.1007/s10570-020-03228-0.
(61) Webb, R. G.; Haskell, M. W.; Stammer, C. H. A Nuclear Magnetic Resonance Method for Distinguishing .Alpha.-Amino Acids from .Beta. and .Gamma. Isomers. *J. Org. Chem.* **1969**, *34* (3), 576–580. https://doi.org/10.1021/jo01255a020.
(62) Häckl, K.; Mühlbauer, A.; Ontiveros, J. F.; Marinkovic, S.; Estrine, B.; Kunz, W.; Nardello-Rataj, V. Carnitine Alkyl Ester Bromides as Novel Biosourced Ionic Liquids, Cationic Hydrotropes and Surfactants. *Journal of Colloid and Interface Science* **2018**, *511*, 165–173. https://doi.org/10.1016/j.jcis.2017.09.096.
(63) Niemczak, M.; Sobiech, Ł.; Grzanka, M. Iodosulfuron-Methyl-Based Herbicidal Ionic Liquids Comprising Alkyl Betainate Cation as Novel Active Ingredients with Reduced Environmental Impact and Excellent Efficacy. *J. Agric. Food Chem.* **2020**, *68* (47), 13661–13671. https://doi.org/10.1021/acs.jafc.0c05850.
(64) Stachowiak, W.; Smolibowski, M.; Kaczmarek, D. K.; Rzemieniecki, T.; Niemczak, M. Toward Revealing the Role of the Cation in the Phytotoxicity of the Betaine-Based Esterquats Comprising Dicamba Herbicide. *Science of The Total Environment* **2022**, *845*, 157181. https://doi.org/10.1016/j.scitotenv.2022.157181.
(65) Duan, B.; Shao, X.; Han, Y.; Li, Y.; Zhao, Y. Mechanism and Application of Ultrasound-Enhanced Bacteriostasis. *Journal of Cleaner Production* **2021**, *290*, 125750. https://doi.org/10.1016/j.jclepro.2020.125750.
(66) Tehrani-Bagha, A. R.; Holmberg, K. Cationic Ester-Containing Gemini Surfactants: Physical−Chemical Properties. *Langmuir* **2010**, *26* (12), 9276–9282. https://doi.org/10.1021/la1001336.
(67) Satam, R. M.; Sawant, M. R. Synthesis of Amphoteric Surfactants via Esterification Process. *Journal of Dispersion Science and Technology* **2006**, *27* (3), 407–411. https://doi.org/10.1080/01932690500359749.
(68) Parus, A.; Homa, J.; Radoński, D.; Framski, G.; Woźniak-Karczewska, M.; Syguda, A.; Ławniczak, Ł.; Chrzanowski, Ł. Novel Esterquat-Based Herbicidal Ionic Liquids Incorporating MCPA and MCPP for Simultaneous Stimulation of Maize Growth and Fighting Cornflower. *Ecotoxicology and Environmental Safety* **2021**, *208*, 111595. https://doi.org/10.1016/j.ecoenv.2020.111595.
(69) Fatma, N.; Panda, M.; Kabir-ud-Din; Beg, M. Ester-Bonded Cationic Gemini Surfactants: Assessment of Their Cytotoxicity and Antimicrobial Activity. *Journal of Molecular Liquids* **2016**, *222*, 390–394. https://doi.org/10.1016/j.molliq.2016.07.044.
(70) Bin-Hudayb, N. S.; Badr, E. E.; Hegazy, M. A. Adsorption and Corrosion Performance of New Cationic Gemini Surfactants Derivatives of Fatty Amido Ethyl Aminium Chloride with Ester Spacer for Mild Steel in Acidic Solutions. *Materials* **2020**, *13* (12), 2790. https://doi.org/10.3390/ma13122790.
(71) Mero, A.; Mezzetta, A.; Nowicki, J.; Łuczak, J.; Guazzelli, L. Betaine and L-Carnitine Ester Bromides: Synthesis and Comparative Study of Their Thermal Behaviour and Surface Activity. *Journal of Molecular Liquids* **2021**, *334*, 115988. https://doi.org/10.1016/j.molliq.2021.115988.
(72) Panda, M.; Fatma, N.; Kamil, M. Synthesis, Characterization and Solution Properties of Novel Cationic Ester-Based Gemini Surfactants. *Zeitschrift für Physikalische Chemie* **2019**, *233* (5), 707–720. https://doi.org/10.1515/zpch-2017-1000.
